# Age Distribution of Multiple Functionally Relevant Subsets of CD4+ T Cells in Human Blood Using a Standardized and Validated 14-Color EuroFlow Immune Monitoring Tube

**DOI:** 10.3389/fimmu.2020.00166

**Published:** 2020-02-27

**Authors:** Vitor Botafogo, Martín Pérez-Andres, María Jara-Acevedo, Paloma Bárcena, Georgiana Grigore, Alejandro Hernández-Delgado, Daniela Damasceno, Suzanne Comans, Elena Blanco, Alfonso Romero, Sonia Arriba-Méndez, Irene Gastaca-Abasolo, Carlos Eduardo Pedreira, Jacqueline A. M. van Gaans-van den Brink, Véronique Corbiere, Françoise Mascart, Cécile A. C. M. van Els, Alex-Mikael Barkoff, Andrea Mayado, Jacques J. M. van Dongen, Julia Almeida, Alberto Orfao

**Affiliations:** ^1^Translational and Clinical Research Program, Centro de Investigación del Cáncer (CIC) and Instituto de Biología Molecular y Celular del Cancer (IBMCC), CSIC-University of Salamanca (USAL), Salamanca, Spain; ^2^Cytometry Service, NUCLEUS, Department of Medicine, University of Salamanca (USAL) and Institute of Biomedical Research of Salamanca (IBSAL), Salamanca, Spain; ^3^Biomedical Research Networking Centre Consortium of Oncology (CIBERONC) (CB16/12/00400), Instituto de Salud Carlos III, Madrid, Spain; ^4^Clinical Medicine Postgraduate Program, Faculty of Medicine, Federal University of Rio de Janeiro, Rio de Janeiro, Brazil; ^5^Sequencing Service, NUCLEUS, University of Salamanca (USAL) and Institute of Biomedical Research of Salamanca (IBSAL), Salamanca, Spain; ^6^Cytognos SL, Salamanca, Spain; ^7^Department of Immunohematology and Blood Transfusion (IHB), Leiden University Medical Center (LUMC), Leiden, Netherlands; ^8^Miguel Armijo Primary Health Care Centre, Sanidad de Castilla y León (SACYL), Salamanca, Spain; ^9^Pediatrics Service, University Hospital of Salamanca, Salamanca, Spain; ^10^Gynecology and Obstetrics Service, University Hospital of Salamanca, Salamanca, Spain; ^11^Systems and Computing Department (PESC), COPPE, Federal University of Rio de Janeiro (UFRJ), Rio de Janeiro, Brazil; ^12^Centre for Infectious Disease Control, National Institute for Public Health and the Environment (RIVM), Bilthoven, Netherlands; ^13^Laboratory of Vaccinology and Mucosal Immunity, Université libre de Bruxelles (ULB), Brussels, Belgium; ^14^Immunobiology Clinic, Hôpital Erasme, Brussels, Belgium; ^15^Institute of Biomedicine, Department of Microbiology, Virology and Immunology, University of Turku (UTU), Turku, Finland

**Keywords:** CD4+ T-cell subsets, flow cytometry, immune monitoring, Tregs, TFH, Th-cell subsets, age-related values

## Abstract

CD4+ T cells comprise multiple functionally distinct cell populations that play a key role in immunity. Despite blood monitoring of CD4+ T-cell subsets is of potential clinical utility, no standardized and validated approaches have been proposed so far. The aim of this study was to design and validate a single 14-color antibody combination for sensitive and reproducible flow cytometry monitoring of CD4+ T-cell populations in human blood to establish normal age-related reference values and evaluate the presence of potentially altered profiles in three distinct disease models—monoclonal B-cell lymphocytosis (MBL), systemic mastocytosis (SM), and common variable immunodeficiency (CVID). Overall, 145 blood samples from healthy donors were used to design and validate a 14-color antibody combination based on extensive reagent testing in multiple cycles of design–testing–evaluation–redesign, combined with *in vitro* functional studies, gene expression profiling, and multicentric evaluation of manual vs. automated gating. Fifteen cord blood and 98 blood samples from healthy donors (aged 0–89 years) were used to establish reference values, and another 25 blood samples were evaluated for detecting potentially altered CD4 T-cell subset profiles in MBL (*n* = 8), SM (*n* = 7), and CVID (*n* = 10). The 14-color tube can identify ≥89 different CD4+ T-cell populations in blood, as validated with high multicenter reproducibility, particularly when software-guided automated (vs. manual expert-based) gating was used. Furthermore, age-related reference values were established, which reflect different kinetics for distinct subsets: progressive increase of naïve T cells, T-helper (Th)1, Th17, follicular helper T (TFH) cells, and regulatory T cells (Tregs) from birth until 2 years, followed by a decrease of naïve T cells, Th2, and Tregs in older children and a subsequent increase in multiple Th-cell subsets toward late adulthood. Altered and unique CD4+ T-cell subset profiles were detected in two of the three disease models evaluated (SM and CVID). In summary, the EuroFlow immune monitoring TCD4 tube allows fast, automated, and reproducible identification of ≥89 subsets of CD4+ blood T cells, with different kinetics throughout life. These results set the basis for in-depth T-cell monitoring in different disease and therapeutic conditions.

## Introduction

The heterogeneous CD4+ T cells coordinate adaptive immune responses via secretion of cytokines and direct cell-to-cell contact (1). Once primed, CD4+ T cells migrate via blood both to lymphoid tissues to help B cells produce antibodies and to peripheral sites of antigen exposure to eliminate incoming pathogens by delivering the appropriate effector response(s) through recruitment/activation of a wide variety of antigen-specific and innate cells (1, 2). CD4+ T cells comprise multiple functional subsets, including different subpopulations of T-helper (Th) cells [i.e., both classical Th cells, such as the Th1, Th2, and Th17 subsets (1, 3, 4), and non-classical Th1/Th17, Th22, Th9, or Th25 cells (1, 5)] each orchestrating different immune responses (2), regulatory T cells (Tregs) (1, 2), and follicular helper T (TFH) cells (1, 6). Since these multiple functionally distinct populations of CD4+ T cells play a critical role in coordinating immune responses, monitoring of their kinetics has become relevant in distinct conditions such as autoimmune and inflammatory diseases (7), allergy (8, 9), organ/tissue transplantation (10, 11), classical and novel targeted therapies (e.g., antitumor immunotherapy) (12), and vaccination (13–15).

The great majority of the CD4+ T-cell subsets are predominantly located in lymphoid and peripheral tissues, where they exert their effector functions, but many of them recirculate in blood prior and after reaching their targeted tissue, which makes them detectable in blood at variable (usually low) frequencies (16). Consequently, minimally invasive blood monitoring might still be possible to dissect the composition of the CD4+ T-cell compartment in clinical settings, if sufficient cells are acquired.

The diverse functional subsets of CD4+ T cells, particularly Th cells, were first identified based on their unique *in vitro* profiles of cytokine secretion (1, 17, 18). However, this requires *in vitro* culture for variable periods of time (19, 20), which is time-consuming and very difficult to standardize for the clinical settings (20). To overcome these limitations, identification of the major subsets of CD4+ T cells has also been performed in the last decades based on their surrogate cell surface membrane phenotypes, by both multiparameter flow cytometry (MFC) (21–25) and mass cytometry (26–30). Thus, different panels of monoclonal antibodies (mAbs) directed against several cell surface chemokine receptors, intracellular transcription factors, and other markers have been proposed (10, 21, 31–33) for the identification of the main CD4+ T-cell subsets. However, the specific link between many CD4+ T-cell phenotypes and their corresponding genomic/functional profiles still remains to be confirmed in humans. In turn, almost every strategy proposed so far for antibody panel design and data analysis strongly relies on subjective “expert-shared” consensus, in the absence of standardized and validated methods that would guarantee multicentric reproducibility of CD4+ T-cell subset monitoring in clinical settings.

Here, we designed and validated a single 14-color antibody combination for automated standardized and reproducible identification and monitoring of ≥89 distinct (e.g., functionally relevant) CD4+ T-cell populations in human blood, established age-related reference values, and investigated the presence of altered CD4+ T-cell subset profiles in three disease conditions—monoclonal B-cell lymphocytosis (MBL), systemic mastocytosis (SM), and common variable immunodeficiency (CVID)—setting the basis for application in routine clinical practice.

## Methods

### Samples

Overall, 268 peripheral blood (PB) samples from an identical number of different donors −113 females (f) and 155 males (m) with median age of 42 years (range: 2 months to 89 years)—and 15 cord blood (CB) samples were studied. All samples were obtained from European Caucasian donors. For antibody panel design, 89 (78 EDTA-anticoagulated and 11 heparin-anticoagulated) PB samples from nine children [3 f/6 m with a median age of 5 years (range: 5 days to 11 years)] and 80 healthy adults [38 f/42 m; median age of 30 years (range: 25–84 years)] were used. To evaluate reproducibility of expert-based manual gating, five additional PB samples were used. In turn, for *in vitro* stimulation and gene expression profiling (GEP) assays, another 11 and 6 healthy adult PB samples were used, respectively. For multicenter testing of the final version of the EuroFlow immune monitoring (IMM) TCD4 tube and construction of the reference database for automated gating (34), an additional set of 34 EDTA-anticoagulated adult PB samples −16 f/18 m with a median age of 45 years (range: 22–65 years)—was used. Normal age-related reference values for the distinct populations of CD4+ T cells identified in blood were defined based on a group of 15 EDTA-anticoagulated CB and 98 PB samples −34 f/64 m with a median age of 45 years (range: 2 months to 89 years)—from a total of 113 healthy donors. The distribution of CD4+ T cells was also evaluated in PB samples from eight patients with MBL [1 f/7 m with a median age of 69 years (range: 52–89 years)], seven patients with SM [4 f/3 m with a median age of 59 years (range: 48–75 years)], and 10 patients with CVID [6 f/4 m with a median age of 44 years (range: 24–67 years)]. Prior to sample collection, written informed consent was given by each donor and/or his/her legal representative(s) according to the Declaration of Helsinki; the study was approved by the local ethics committees of all participating PERISCOPE centers.

### Design of the EuroFlow-IMM TCD4 Antibody Combination

A total of 73 distinct (commercially available) fluorochrome-conjugated mAbs were evaluated in multiple consecutive rounds of design–testing–evaluation–redesign (Supplementary Table 1), aiming at unequivocal and reproducible identification in a single tube of the most relevant functional subsets of CD4+ T cells and their maturation stages. Fresh (<24 h) PB samples were stained using EuroFlow standard operating procedures (SOP) (35) for staining of surface markers only or for combined staining of intracellular and surface membrane markers, as detailed in the Supplementary Methods and in the EuroFlow website (SOPs are freely available in full at www.EuroFlow.org).

First, markers for general identification of CD4+ T cells and their maturation stages were tested in 23 PB samples (9 children and 14 adults). Principal component analysis (PCA) based on the automatic population separator (APS) tool of Infinicyt™ software (Cytognos S.L., Salamanca, Spain) (36) was used to identify those markers that provided independent (non-redundant) information for identification of CD4+ T-cell maturation stages. Maturation diagrams were then generated with the Infinicyt™ software based on multiparametric analysis of CD4+ T cells (PCA) visualized in APS plots, as previously described (37). Twenty maturation stages were defined by default for smooth graphical representation along the maturation pathway of each cell population. Subsequently, markers for classical Th cells, Tregs, and TFH cells were tested in 37 adult PB samples, and the phenotypes they provided were directly correlated with their cytokine production profiles obtained through *in vitro* stimulation of whole heparin-anticoagulated PB samples (*n* = 9). Based on these tests, a first version of the EuroFlow-IMM TCD4 tube was designed (Table 1). Subsequent versions included addition of a T-cell activation-associated marker (version 2) and of CD45 (version 3) (Table 1). These latter two markers were selected based on (i) parallel testing for CD69, CD154, and HLADR in two heparin-anticoagulated PB samples (version 2) and (ii) comparison of gating results with and without CD45 (version 3). Subsequent validation of the new antibody combinations (versions 2 and 3) was performed in 6 and 12 EDTA-anticoagulated adult PB samples, respectively.

**Table 1:** Antibody combinations tested during the “design–test–evaluation–redesign” cycles and the resulting combinations of markers sequentially tested from the first version (version 1) to the final version (version 3) of the complete EuroFlow immune monitoring (IMM) TCD4 tube.

**Fluorochrome conjugate**														
***Antibody combinations used and resulting versions (V)***	**BV421**	**BV510**	**BV605**	**BV650**	**BV711**	**BV786**	**VioBright-FITC**	**PerCPcy5.5**	**PE**	**PE-CF594**	**PE-Cy7**	**APC**	**AF700**	**APCH7**
*First design–test–evaluation–redesign cycle for selection of maturation markers*	**CD27**	**CD45RA**	–	**CD62L**	**CD4**	**CD3**	**CD57**	**CD28**	**CD197**	–	–	**CD95**	–	**CD45RO**
*Second design–test–evaluation–redesign cycle for selection of Th-associated markers, Tregs, and TFH*	**CD127**	–	–	–	**CD4**	**CD3**	**CD25**	–	**cyTbet**	–	**cyGATA3**	**cyRORγt**	–	–
	–	–	–	–	**CD4**	**CD3**	**CD195**	**CD196**	**CD183**	–	**CD194**	**CD161**	–	**CD45RO**
	–	–	–	–	**CD4**	**CD3**	**CD294**	**CCR10**	**CD183**	–	**CD194**	**CD161**	–	**CD45RO**
	–	**CD127**	–	–	**CD4**	**CD3**	–	–	**CD39**	–	**CD25**	**CD15s**	–	–
	–	**CD127**	–	**CD278**	**CD4**	**CD3**	**CD25**	–	–	–	**CD279**	–	–	–
	–	**CD127**	–	–	–	–	**CD25**	**CD3**	**cyFoxp3**	–	–	–	–	**CD4**
	**CD84**	**CD7**	–	**CD278**	**CD4**	**CD3**	–	**CD5**	**CD279**	**CD272**	**CD10**	**CD185**	–	–
*V1*	**CD27** M-T271 BD	**CD45RA** HI100 BD	–	**CD62L** DREG-56 BL	**CD127** HIL7RM21 BD	**CD3** SK7 BD	**CD25** 4E3 Miltenyi	**CCR10** 1B5 BD	**CD183** 1C6/CXCR3 BD	**CD196** 11A9 BD	**CD194** L291H4 BL	**CD185** REA103 Miltenyi	–	**CD4** SK3 BD
*V2*	**CD27** M-T271 BD	**CD45RA** HI100 BD	**cyCD154** 24-31 BL	**CD62L** DREG-56 BL	**CD127** HIL7RM21 BD	**CD3** SK7 BD	**CD25** 4E3 Miltenyi	**CCR10** 1B5 BD	**CD183** 1C6/CXCR3 BD	**CD196** 11A9 BD	**CD194** L291H4 BL	**CD185** REA103 Miltenyi	–	**CD4** SK3 BD
*V3*	**CD27** M-T271 BD	**CD45RA** HI100 BD	**cyCD154** 24-31 BL	**CD62L** DREG-56 BL	**CD127** HIL7RM21 BD	**CD3** SK7 BD	**CD25** 4E3 Miltenyi	**CCR10** 1B5 BD	**CD183** 1C6/CXCR3 BD	**CD196** 11A9 BD	**CD194** L291H4 BL	**CD185** REA103 Miltenyi	**CD45** HI30 BD	**CD4** SK3 BD

*AF, Alexa Fluor; APC, allophycocyanin; APCCy7, allophycocyanin–cyanin 7; APCH7, allophycocyanin–hilite 7; BD, Becton/Dickinson Biosciences; BL, BioLegend; BV, Brilliant Violet; cy, intracellular; eBio, eBioscience; FITC, fluorescein isothiocyanate; PerCPCy5.5, peridinin–chlorophyll protein–complex cyanin 5.5; PE, phycoerythrin; PE-Cy7, PE–cyanin 7. Below the specificity of each monoclonal antibody, the clone and the source are indicated for versions 1, 2, and 3 (for all the other antibodies used in the design test–evaluation–redesign cycles, detailed information for each reagent is provided in Supplementary Table 1). Fluorescent molecules and monoclonal antibodies conjugated to these molecules are in bold*.

For flow cytometric design and evaluation studies, medians of 221,871 total live leucocytes (range: 118,068–732,395 cells) and 59,748 live T cells (range: 25,228–177,058 cells) were measured per tube in LSRFortessa X-20 [Becton/Dickinson Biosciences (BD) San Jose, CA] instruments, using the FACSDiva software (BD). For instrument setup and data acquisition, the EuroFlow SOP for instrument setup was used (35) (SOPs are freely available in full at www.EuroFlow.org). The Infinicyt™ software was employed for data analysis. Comparison between antibody reagents directed against the same molecule were based on their staining profiles on CD4+ T cells (vs. other PB non-T lymphocytes) as defined by median fluorescence intensity (MFI) and stain index values, aiming at optimal separation of the target populations, as previously described (38).

### *In vitro* T-Cell Stimulation Assays

Well-established short-term *in vitro* cell culture assays were used to directly evaluate the correlation between specific CD4+ Th-cell phenotypes (e.g., chemokine receptor-based) and intracellular cytokine production profiles and to select an optimal T-cell activation-associated marker (Supplementary Methods; Supplementary Table 2) (39).

### GEP Studies

To confirm the association between phenotypic and genotypic profiles of normal adult blood CD4+ T cells, 22 different well-defined CD4+ T-cell subsets were sorted through FACS (FACSAria III, BD) from Biocoll-enriched PB-derived (*n* = 6) mononuclear cells (final purity >97%) as described in detail in the Supplementary Methods, Supplementary Table 3, and Supplementary Figure 1. Total RNA was extracted from each purified CD4+ T-cell population using the NucleoSpin® RNA XS kit (Macherey-Nagel, Düren, Germany), transcribed into cDNA and amplified using a quantitative real-time polymerase chain reaction (qPCR). Selection of conventional qPCR (vs. RNA-seq) was based on the following criteria: (i) most RNA-seq protocols recommend an input of 1 μg of RNA (40), therefore requiring the collection of significantly higher numbers of cells, which could not be easily obtained in our settings for each of the cell populations investigated (median of 15,000 cells per sorted CD4+ T-cell subset; range: 3,000–50,000), considering that the RNA concentration is around 2.5 pg/cell in human PB (41), and (ii) for the validation purposes of our study, evaluation of a limited set of well-established genes related with specific Th, Treg, and TFH patterns should be sufficient. The Biomark HD Platform (Fluidigm, San Francisco, CA) was used to asses GEP for a panel of 85 genes (Supplementary Table 4) including GAPDH and KIT as positive and negative controls, respectively. For data analysis, GEP raw data were normalized by dividing each gene expression value by their corresponding GAPDH expression value (positive control) in all technical replicates (*n* = 5) obtained for each sample (*n* = 6 PB samples). Average expression values for each of the 85 genes investigated in the technical replicates measured for each sample were calculated, and data were analyzed and represented in a heatmap graphic with the corresponding hierarchical clustering diagram. R-package gplot (R Development Core Team, Vienna, Austria) was used to generate the cluster heatmap (**Figure 2**) in which averaged gene expression values, standardized as *Z*-values, are shown in a color code; one-tailed *p*-values were calculated using multiscale bootstrap resampling. Hierarchical trees were generated with the R-package pvclust algorithm (R Development Core Team v.3.2.3, Vienna, Austria). Data analysis and data representation were performed at the Bioinformatics Service (NUCLEUS) of USAL.

### Reproducibility of Expert-Based Manual Data Analysis

Reproducibility of expert-based manual gating of flow cytometry standard (FCS) data files was evaluated in five adult PB samples stained with the EuroFlow IMM TCD4 panel (version 3), based on (independent) analysis by an experienced (E1) flow cytometrist and a less experienced (E2) flow cytometrist. To establish intra-operator and inter-operator variability, analyses of the same FCS files (by both experts) were repeated twice within a 6-months interval.

### Database Construction, Automated Gating and Multicentric Validation of the EuroFlow IMM TCD4 Tube

To build a database and evaluate the reproducibility of results obtained with the EuroFlow IMM TCD4 tube in four PERISCOPE centers, 34 PB samples from healthy adults were stained using version 3 of the antibody panel and measured in LSRFortessa X-20 instruments, in the framework of a Horizon 2020/Innovative Medicines Initiative (IMI) multisite consortium (PERISCOPE; http://periscope-project.eu/) at four centers: Universidad de Salamanca (USAL) (*n* = 18), Leiden University Medical Center (LUMC) (*n* = 5), Rijksinstituut voor Volksgezondheid en Milieu (RIVM) (*n* = 8), and University of Turku (UTU) (*n* = 3). To evaluate intra- and inter-center variability, PCA and canonical multivariate analyses (CA) were used with Infinicyt™. Based on the same data files, a database was built with the data (.FCS files) from the four centers; inclusion criteria for valid FCS files are detailed in the Supplementary Methods. Afterward, automated gating using the database and the automated gating tool of Infinicyt™ were prospectively validated against expert E1-based manual gating in five out-of-sample blood specimens stained with the EuroFlow IMM TCD4 tube. Database-guided automated gating results obtained at two different time points set 6 months apart were also compared. For automated gating, ≥10 events were required by the software to define a cell cluster.

### Distribution of CD4+ T-Cell Subsets in Healthy Donors Grouped by Age and in Pathological Conditions

Age-related reference values for absolute CD4+ T-cell subset counts in human blood were established for each CD4+ T-cell population identified with the EuroFlow IMM TCD4 tube, based on 10th–90th percentile values (R statistical v.3.2.3) obtained for a total of 113 (15 CB and 98 PB) samples. To compare the distribution of CD4+ T-cell subsets in blood of healthy donors and subjects with MBL, SM, and CVID, the GraphPad Prism 5 (San Diego, CA) software package was used. The Mann–Whitney *U* test was employed to investigate the statistical significance (set at *p* < 0.05) of differences in the distribution of CD4+ T-cell populations in blood of the patients vs. age-matched healthy donors.

## Results

### Selection of Maturation-Associated Markers for CD4+ T-Cell Subsetting

**Figure 1:**
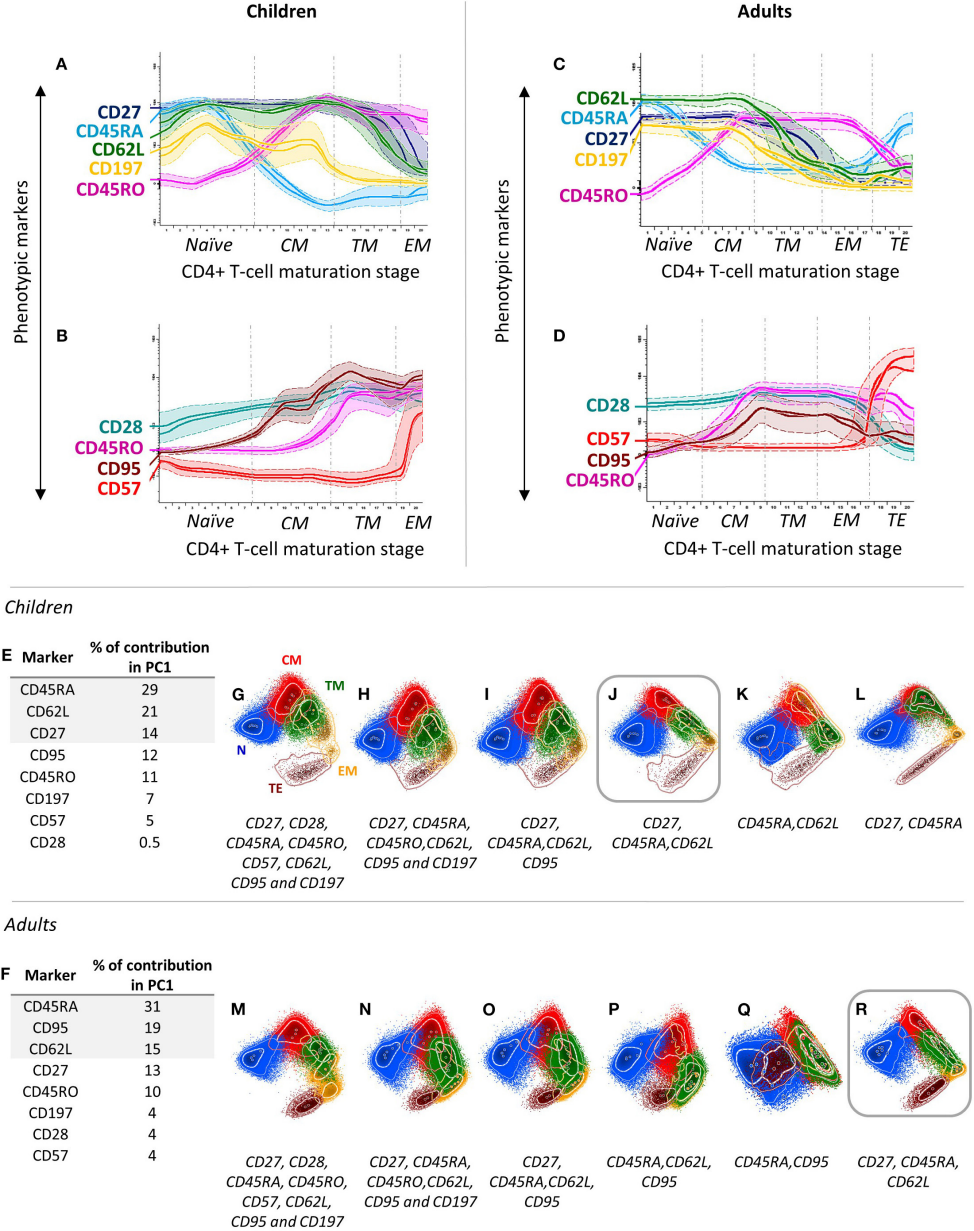
Stepwise process used for selection and evaluation of non-redundant maturation-associated markers for inclusion in the EuroFlow immune monitoring (IMM) TCD4 tube. Redundant maturation markers (i.e., CD45RO, CD95, and CD197) were identified based on analysis of blood CD4+ T cells from two healthy children (aged 2 and 9 years) **(A,B)** and two healthy adults (two 40-year-old donors) **(C,D)**. In the maturation diagrams **(A–D)**, colored (solid) lines represent the level of expression of markers per maturation stage corresponding to each donor, while dotted lines represent one standard deviation (SD). The CD4+ T-cell maturation pathway was automatically divided into 20 stages, ranging from the less mature (1) to the most mature (20) immunophenotype based on the *Maturational Pathway Tool* of the Infinicyt software. By combining stages with comparable immunophenotypes for CD27, CD45RA, and CD62L, the arbitrary 20 stages were reduced to five distinct maturation stages: (i) naïve (N) cells, CD27+CD45RA+CD62L+; (ii) central memory (CM), CD27+CD45RA–CD62L+; (iii) transitional memory (TM), CD27+CD45RA–CD62L–; (iv) effector memory (EM), CD27–CD45RA–CD62L±; and (v) terminal effector (TE), CD27–CD45RA+CD62L–. Additionally, a combination of eight maturation-associated markers was evaluated in PB samples from healthy children (*n* = 5) **(E)** and adults (*n* = 3) **(F)** using principal component (PC) analysis. Different combinations of these markers were tested using PC1 vs. PC2 automatic population separator (APS1) plots to select the markers providing the best resolution (separation) between the different maturation stages of CD4+ T cells **(G–R)**. According to their percentage contribution in PC1 **(E,F)**, markers were sequentially removed from APS1 plots (from lower to higher), as indicated **(G–R)**, to determine the minimum combination of markers required for reliable identification of all five major stages of maturation of CD4+ T cells (please see above: N, CM, TM, EM, and TE cells). APS1 plots with the best resolution power are highlighted with squares. In all PC1-vs.-PC2 plots, solid circles represent median values for the parameters evaluated; inner (dotted) and outer (solid) lines represent the first and second SDs for each population identified, color-coded as follows: N, blue; CM, red; TM, green; EM, yellow; and TE, brown, CD4+ T cells.

For identification of total CD4+ T cells, well-established CD3 and CD4 antibody clones were first selected (Table 1). In addition, 10 distinct maturation-associated markers (Figures 1A–D; Supplementary Figure 2) were also selected from the literature (4, 22, 33) for further testing in children and in adult PB. Subsequently, all 12 markers mentioned above were stained together, and the profiles for the 10 CD4+ T-cell maturation-associated markers were compared via multivariate analysis (PCA) of single cells to identify the optimal combination of markers for maturation-related subsetting of blood CD4+ T cells (Figures 1E–R). A minimum combination of three non-redundant maturation-related markers (CD27, CD45RA, and CD62L) was identified and selected, which allowed full delineation of the CD4+ T-cell maturation stages including naïve (N), central memory (CM), transitional memory (TM), effector memory (EM), and terminal effector (TE) T cells (Table 1 and Figure 1). In fact, CD27 provided clear discrimination between TM and EM cells, as revealed via PCA (Figures 1J,R), with higher contribution (Figures 1E,F) for this marker in PC1 than for that CD28 and CD95, both in children and in adults (Figures 1H,N,J,R). CD45RA and CD45RO showed a typical mirror image (Figures 1A,C), the former having a greater contribution in PC1 for identification of naïve CD4+ T cells, both in children and in adults (Figures 1E,F). Finally, CD62L and CD197 showed similar (parallel) expression profiles (Figures 1A,C), the former displaying a higher contribution for discrimination of the major maturation subsets of CD4+ T cells (Figures 1J,R), with less lot-to-lot reagent variability (Supplementary Table 1). CD31, cyTCL1, and CD57 were not selected because their expression was restricted to subsets of naïve T cells [e.g., recent thymic emigrants (RTEs)] (42) (Supplementary Figure 2) and a fraction of TE CD4+ T cells (Figures 1B,D), and they did not add significantly to the discrimination among the major CD4+ T-cell maturation stages already achieved with CD27, CD45RA, and CD62L (Figures 1J,R). Therefore, these latter three maturation-associated markers were selected for the EuroFlow IMM TCD4 tube.

### Selection of Markers for Identification of CD4+ Th-Cell Subsets

Subsequently, markers for identification of classical Th cells were tested, including (i) chemokine receptors previously found to identify the major Th subsets (1, 21) and (ii) Th subset-associated transcriptional factors (1, 2). Thus, staining for cell surface CD183 (CXCR3), CD194 (CCR4), CD196 (CCR6), CD195 (CCR5), CD294 (CRTH2), CD161, and CCR10 and intracellular Tbet, GATA3, and RORγt was tested (Table 1). The first three membrane markers proved accurate to identify cells producing IFNγ (Th1), IL4+IL5 (Th2), and IL17A (Th17) producing cells, respectively (Supplementary Figure 3), while CCR10 was chosen for identification of Th22-cells (5, 22) (data not shown). The other surface markers (CD195, CD294, and CD161) and intracellular transcription factors (Tbet, GATA3, and RORγt) were of no additional value for identification of the above Th subsets of CD4+ T cells, as they were only partially positive in Th1 (CD195 and Tbet), Th2 (CD294 and GATA3), and Th17 (CD161 and RORγt) cells, respectively (Supplementary Figure 3). Based on the four markers selected, the phenotypic profiles of classical Th-cell subsets were as follows: (i) Th1 cells were CD183+CD194–CD196–CCR10–; (ii) Th2, CD183–CD194+CD196–CCR10–; (iii) Th17, CD183–CD194+CD196+CCR10–; (iv) Th1/Th17, CD183+CD194–CD196+CCR10–; and (v) Th22, CD183–CD194+CD196+CCR10+.

For Tregs, CD127 and CD25 were chosen based on the high correlation observed with cyFoxP3+ cells in children and in adult PB and a more reproducible staining profile, in line with previous findings (43–45). Other Treg-associated markers tested (Supplementary Table 1), such as CD39 and CD15s, discriminated memory Tregs and small subsets of mature Tregs (Supplementary Figure 4). However, the information they provided was redundant. In turn, CD185 (CXCR5) emerged as the most specific TFH-related marker (Supplementary Figure 5), as previously indicated (1, 6). Four different allophycocyanin (APC)-conjugated CD185 antibody clones were tested; from them, the REA103 clone showed the highest stain index (Supplementary Table 5), which should be used strictly following recommended storage conditions (i.e., temperature) for optimal results. Based on the staining profile observed for the 12 markers selected above, a first version of the EuroFlow IMM TCD4 tube was validated (Table 1).

In the following versions, cyCD154 (version 2) and CD45 (version 3) were added based on the close association found between cyCD154 expression (vs. CD69 and HLADR) and *in vitro* activated cytokine-secreting CD4+ T cells (Supplementary Figure 6, Table 1) and the utility of CD45 for identification of all blood leukocytes and their major myeloid vs. lymphoid subsets, respectively. Overall, the final version (version 3) of the EuroFlow IMM TCD4 tube (Table 1) allowed identification of ≥89 CD4+ T-cell subsets, including previously defined and newly described cell populations (Supplementary Table 6). The number of populations identified may reach up to 161 different CD4+ T-cell subsets depending on the sample volume and the number of cells evaluated (data not shown).

### Cytokine Secretion Profiles for the Major Th1, Th2, and Th17 CD4+ T-Cell Subsets Identified in Blood

**Figure 2:**
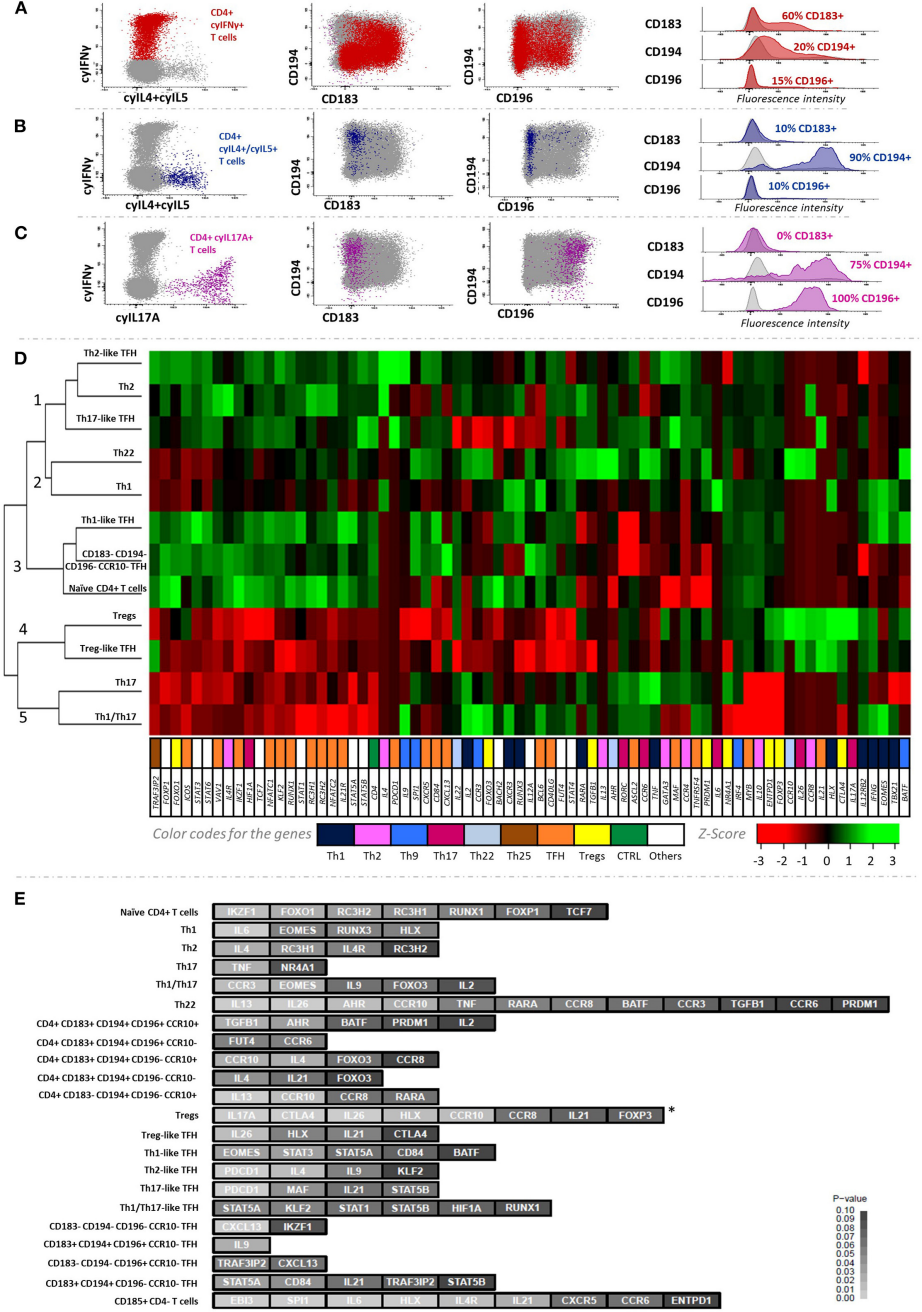
Cytokine secretion profile of the major Th-cell subsets identified in blood and their corresponding gene expression profiles. The association between IFNγ, IL4/IL5, and IL17 production with surface expression of chemokine receptors is depicted in **(A–C)**, respectively. The cluster heatmap in **(D)** shows the expression levels of each gene normalized as *Z*-values corresponding to the 12 main CD4+ T-cell subsets sorted. **(E)** illustrates the discriminatory ability of different groups of genes among the 22 major CD4+ T-cell subsets. *Z*-test was used to calculate the *Z*-scores and one-tailed *p*-values for all genes studied, based on multivariate comparison of their expression among the different CD4+ T-cell subsets (*p* < 0.05; depicted in light gray). *FOXP3 showed significantly (*p* = 0.005) higher expression levels in regulatory T-cell subsets (Tregs + Treg-like TFH cells) vs. non-regulatory classical Th cells (Th1 + Th2 + Th17 + Th1/Th17 + Th22).

The association between the profile of chemokine receptors postulated to identify classical Th1 cells (CD183+CD194–CD196–CCR10–) and the production of IFNγ after *in vitro* stimulation with both PMA+ionomycin and a CMV lysate was only partial, due to downregulation of CD183 expression (Figure 2A) potentially due to internalization of the receptor (46, 47). In turn, IL4+IL5-producing cells after *in vitro* stimulation with PMA+ionomycin showed a CD194^hi^ CD183–CD196– phenotype, as typically described for Th2 cells (Figure 2B), while IL17A-producing cells displayed a unique CD194+CD196+CD183– Th17-related phenotype (Figure 2C).

### Gene Expression Profile of PB CD4+ T-Cell Subsets

Unsupervised hierarchical clustering based on GEP of 12 FACS-sorted subsets of CD4+ T cells showed five main (population) clusters (Figure 2D). In cluster 1, Th2 and Th2-like TFH cells were grouped together mainly due to overexpression of the IL4 Th2-related gene (*p* = 0.03), in the absence of expression of Th1-related genes (i.e., EOMES, IFNG, or TBX21); as expected, Th2-like TFH cells also showed expression of TFH-related genes such as *PDCD1* (*p* = 0.0008). Of note, Th17-like TFH cells were also included in this cluster due to their similar GEP. In turn, cluster 2 contained Th1 and Th22 cells, presenting uncommon low expression of several non-Th1/Th22-related genes, while differing among them in genes overexpressed in Th1 [e.g., the RUNX3 (*p* = 0.05) and EOMES (*p* = 0.05) Th1-related genes] and Th22 cells [e.g., the AHR (*p* = 0.02) Th22-related gene], respectively. Interestingly, Th1-like TFH cells were placed in the neighborhood of Th1 cells, but in different clusters. Thus, the former clustered together with naïve cells with shared GEP for most genes analyzed, except typical Th1-related genes. Of note, Treg and Treg-like TFH subsets clustered in a separate and well-defined group (cluster 4), characterized by overexpression of Treg-related genes (e.g., CTLA4; *p* = 0.002 and *p* = 0.09 for Treg and Treg-like TFH, respectively). Despite FOXP3 being not found to have high discriminatory power in the multivariate comparison among all 22 CD4+ T-cell subsets, this gene was expressed at significantly higher levels (*p* = 0.005) in regulatory vs. non-regulatory CD4+ T-cell populations (Figure 2E). In turn, *IL17A* gene was also found to be significantly overexpressed in Tregs vs. all the other cell subsets here analyzed (*p* = 0.0003). Similarly, Th17 and Th1/Th17 cells were classified together in cluster 5. In common, both cell populations showed high expression of the RORC Th17-related gene in the cluster heatmap analysis (Figure 2D), while Th1/Th17 cells further displayed overexpression (*p* = 0.03) of the EOMES Th1-associated gene (Figures 2D–E; Supplementary Figure 7). Consequently, the classical Th subsets (Th1, Th2, Th17, Th1/Th17, and Th22) showed the expected gene expression profiles. Relative expression values observed for all 85 genes analyzed in all the FACS-sorted blood CD4+ T-cell populations (*n* = 22) from six healthy donors are shown in Supplementary Table 7.

### Reproducibility of Expert-Based (Manual) Data Analysis

Comparison of manual gating of flow cytometry data between experts E1 and E2 showed a high correlation (*r*^2^ ≥ 0.9; *p* ≤ 0.05) and degree of agreement (MNB with ±15%) for virtually all (11/13; 85%) major CD4+ T-cell populations identified with the EuroFlow IMM TCD4 tube (Supplementary Table 8). A high correlation was also observed between experts for most maturation subsets of classical Th cells (19/25; 76%), but with a lower degree of agreement (44%) due to systematic overestimation by E2 (vs. E1) of the percentage of these populations, mainly caused by less restrictive criteria in the gating strategy for the identification of the different cell subsets, because of inexperience. A few minor cell populations representing ≤0.01% of nucleated cells (e.g., TE Th cells or specific subsets of Tregs and TFH cells) were not identified in every sample by the two experts (Supplementary Table 8). Manual analysis performed by E1 at two different time points showed both a high degree of correlation and agreement for the major CD4+ T-cell subsets identified (12/13; 92%) and the great majority (49/76; 64%) of the other less represented CD4+ T-cell subsets (Supplementary Table 8).

### Multicenter Validation, Database Construction, and Automated Gating

**Figure 3:**
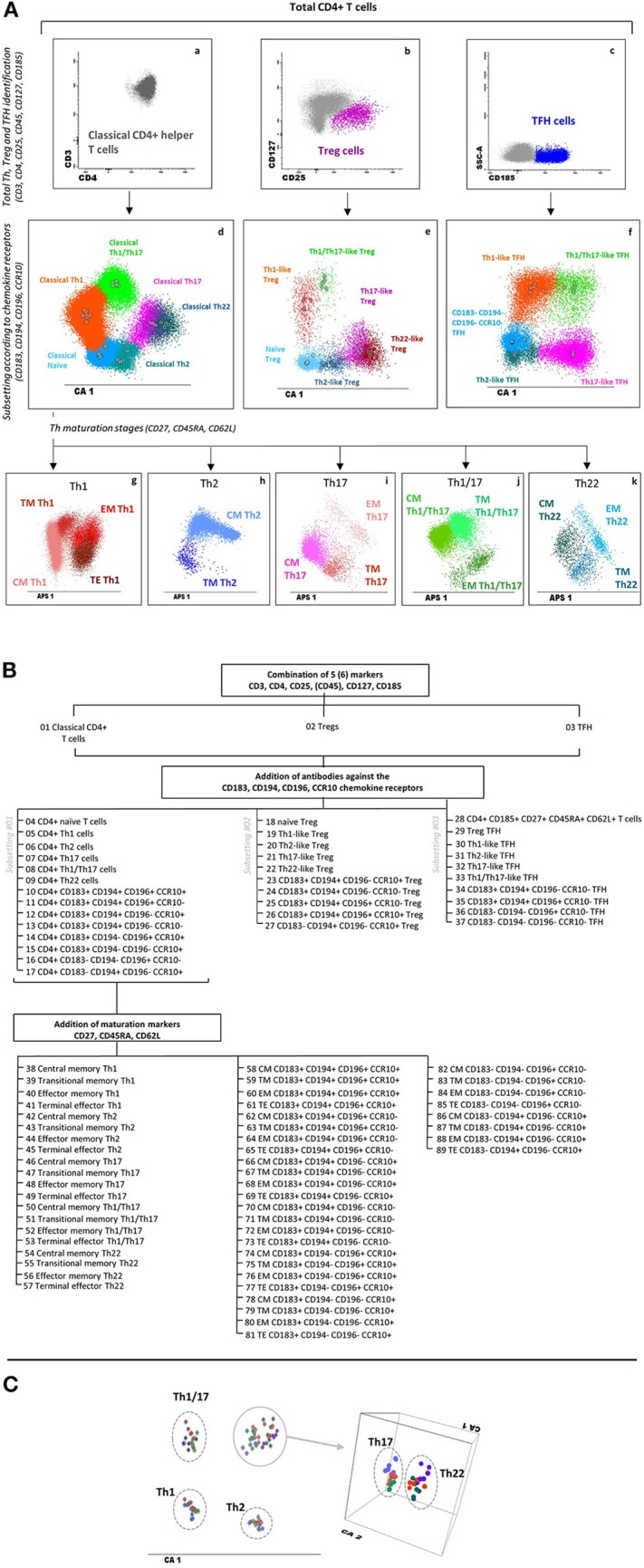
Major T helper, Treg, and TFH populations identified with the EuroFlow immune monitoring (IMM) TCD4 tube and reproducibility of phenotypic data obtained at different centers. **(A)** Sequential strategy used for the identification of total classical helper CD4+ T cells (a), Tregs (b), and TFH cells (c) using CD3, CD4, CD25, CD45, CD127, and CD185. Within each major population, different Th and Th-like subsets are identified based on the expression of CD183, CD194, CD196, and CCR10, as shown in panels d, e, and f for classical Th cells, Tregs, and TFH cells, respectively, via canonical multivariate analysis (CA). Finally, within each Th subset, distinct maturation stages are identified based on their expression profile for CD27, CD45RA, and CD62L, as displayed in two-dimensional automatic population separator (APS) views—principal component (PC)1 vs. PC2—in panels g–k for classical Th cells. **(B)** Dendrogram showing a detailed subsetting of a total of 89 different CD4+ T-cell populations identified with the EuroFlow IMM TCD4 tube. **(C)** CA showing 18 healthy adult blood samples stained at three different centers [Universidad de Salamanca (USAL), *n* = 6; Leiden University Medical Center (LUMC), *n* = 7; Rijksinstituut voor Volksgezondheid en Milieu (RIVM), *n* = 5] to evaluate data reproducibility. Th subsets from USAL, LUMC, and RIVM samples are presented in shades of blue, green, and red, respectively. Solid circles represent median values for the six parameters evaluated (CD3, CD45, CD183, CD194, CD196, and CCR10) in the CA1 diagram. Th17 and Th22 cells are presented in a three-dimensional CA diagram to clearly show separation between these two subsets. Markers contributing to CA1 (percentage contribution) were CD194 (37%), CD183 (32%), CCR10 (17%), CD3 (7%), CD45 (6%), and CD196 (1%). Treg, regulatory T cells; TFH, follicular helper T cells; Th, T helper cells; CM, central memory; TM, transitional memory; EM, effector memory; TE, terminal effector CD4+ T cells.

The EuroFlow IMM TCD4 tube was validated at three different PERISCOPE centers (USAL, LUMC, and RIVM), with fully comparable and reproducible results per center for classical CD4+ Th-cell subsets (Figures 3A–C). Thus, a total of 20/34 FCS data files corresponding to PB samples stained with the EuroFlow IMM TCD4 tube at the distinct centers were used to build a database for automated gating of blood CD4+ T-cell subsets by merging all data files into a single FCS file.

Prospective comparison of database-guided automated gating vs. expert-based manual gating showed a high degree of correlation and agreement for virtually all major CD4+ T-cell populations (11/13; 85%). However, lower levels of correlation and agreement were observed for other less represented cell subsets (Supplementary Table 8), mostly due to (i) heterogeneous marker expression (i.e., for CD25, CD45RA, CD62L, CD194, and CD196), with cross-contamination between different populations (i.e., Tregs and Th2 cells), and (ii) the low frequency (≤0.01% of nucleated cells) of some cell subsets. Comparison between database-guided automated gating performed at two different time points for the same samples showed a correlation and degree of agreement of 100% for all cell populations identified, supporting optimal reproducibility for the automated (vs. manual) gating approach.

### Distribution of CD4+ T-Cell Subsets in Normal Blood Through Life and in Different Disease Conditions

**Figure 4:**
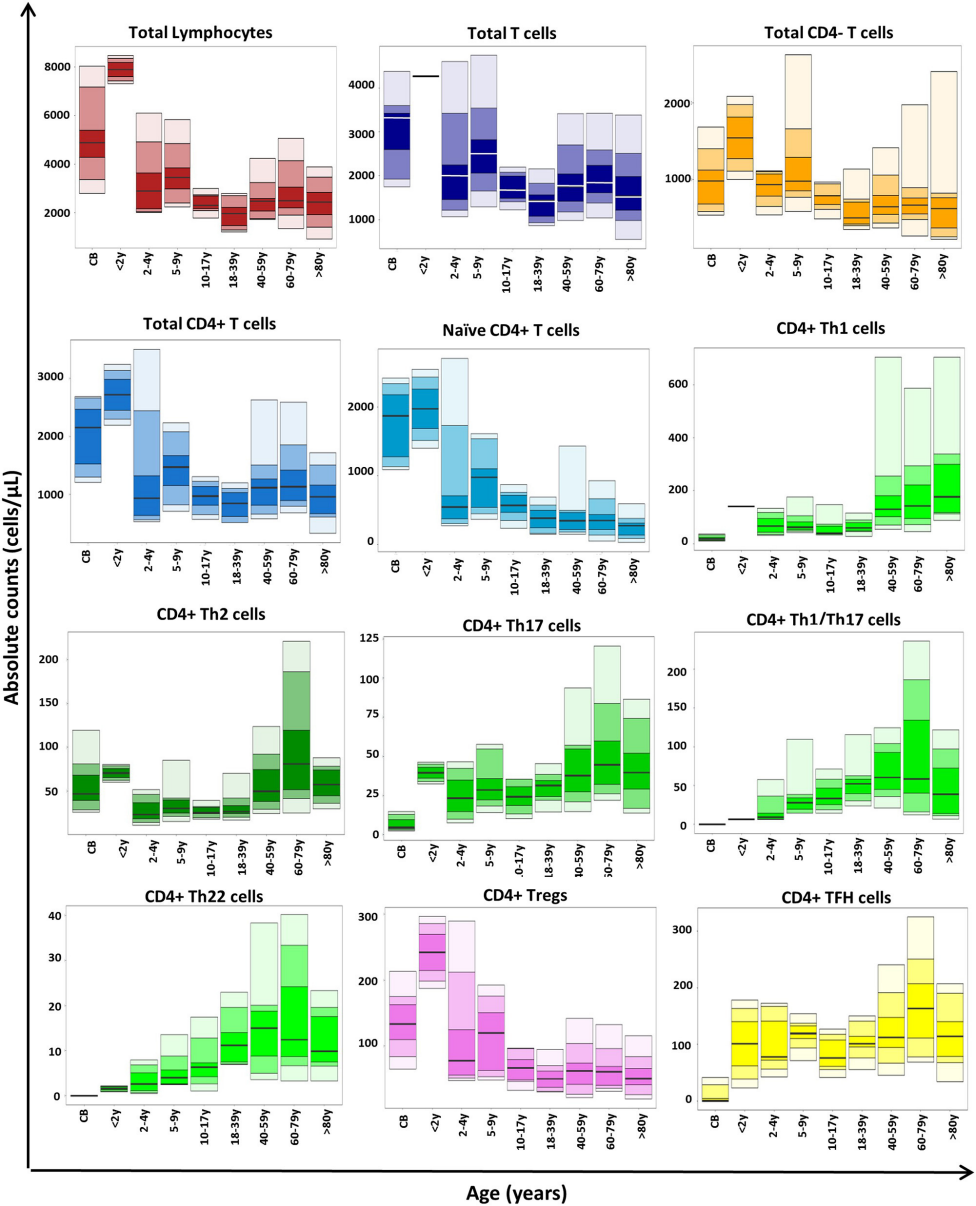
Age-related distribution of CD4+ T-cell subsets in blood as assessed by the EuroFlow immune monitoring (IMM) TCD4 tube in 113 healthy controls. Absolute cell counts are displayed as bars representing median (p50 percentile), minimum, maximum, and p10, p25, p75, and p90 percentile values. All age groups, except children <4 years included >10 samples.

Total T-cell and CD4+ T-cell counts reached maximum levels in infancy (2 months to 2 years), gradually decreased until adulthood (18 years), and remained relatively stable thereafter (see age-related percentile reference values in Figure 4). This pattern was mainly due to the kinetics of naïve CD4+ T cells, which represented the largest CD4+ T-cell compartment in blood early in life, including in CB. In contrast, all classical Th cells (but Th2) and TFH were found at very low numbers in CB. Subsequently, Th1/Th17 and Th22 cells progressively increased until they peaked between the age of 40 and 79 years, while Th1, Th17, and TFH-cell numbers remained stable until adulthood, when they reached their highest levels (at >80 years for Th1 and at 60–79 years for the other subsets). Of note, Th2 cells showed a uniquely distinct profile: higher numbers (vs. other Th cells) in CB with an increase up to 2 years, followed by a significant decrease until the age of 40 years, increasing thereafter (Figure 4; Supplementary Table 9). Regarding Tregs, a similar distribution to that of total (and naïve) CD4+ T cells was observed in blood, with maximum absolute counts during infancy, due to the expansion of the naïve Treg compartment (Figure 4; Supplementary Table 9).

When the EuroFlow IMM TCD4 tube was applied in PB samples from patients diagnosed with MBL, SM, and CVID, distinctly altered profiles were observed. Thus, a significant increase of circulating naïve CD4+ T cells was found in SM, while patients with CVID showed a significant decrease of all CD4+ T-cell subsets, except TFH cells, when compared to age-matched controls. No statistically significant differences were found between MBL and non-MBL subjects from the general population for any of the CD4+ T-cell subsets investigated (Supplementary Figure 8).

## Discussion

The here presented 14-color antibody combination is the first standardized and validated approach for fast, automated, and reproducible identification of between 89 and 161 different CD4+ T-cell subsets in human blood, depending on the sample volume and the number of CD4+ T-cells evaluated. The preliminary age-related reference values set the basis for future immune monitoring in the clinical settings. Previously, several other flow cytometric (10, 21, 31, 32) and mass cytometry (28, 29) multitube antibody panels have been proposed for identification of CD4+ T-cell subsets in the absence of *in vitro* culturing. These panels require multiple tubes/aliquots for identification of the major CD4+ classical Th, Treg, and TFH-cell subsets and their maturation stages, which translates to the identification of less CD4+ T-cell subsets. Thus, to the best of our knowledge, this is the first antibody combination for either flow or mass cytometry that provides detailed dissection of the CD4+ T-cell compartment in human blood in a single measurement, via a set of complementary markers for CD4+ T cells, Treg, TFH, and classical Th subsets and their maturation stages.

Based on simultaneous assessment of 10 maturation-associated markers (21, 23), single-cell-based multivariate data analysis demonstrated that the combination of CD27, CD45RA, and CD62L was sufficient for clear discrimination among naïve, CM, TM, EM, and TE CD4+ T lymphocytes, both in children and in adult blood. Although a more limited number of markers (i.e., CD45RA and CD197) have been proposed in some studies (21, 32) for identification of CD4+ T-cell maturation stages, these panels were based on expert consensus without (actual) prospective validation in children and adults, and they were found here to be insufficient. However, our combination of CD27, CD45RA, and CD62L did not allow identification of RTE due to absence of CD31; nevertheless, this latter marker might be included in extended versions of the tube whenever more detailed subsetting of recently produced/antigen-naive T cells is needed. Similarly, CD95^hi^ stem cell memory CD4+ T cells (48) cannot be identified within naïve-like cells with the proposed maturation markers. However, in line with previous findings (1, 21, 22) and *in vitro* cytokine secretion and GEP studies performed here, further staining for the CD183, CD194, CD196, and CCR10 chemokine receptors allowed reliable identification of (most) stem cell memory CD4+ T cells based on a CD183^lo^ and/or CD194^lo^ naïve CD4+ T-cell profile (48, 49), in addition to the main classical Th subsets (i.e., Th1, Th2, Th17, Th1/Th17, and Th22) and their maturation stages. In contrast, CD195, CD294, and CD161 expressions, as well as positivity for the Tbet, GATA3, and RORγt transcription factors, were restricted to specific subsets of Th1, Th2, and Th17 cells (50, 51), respectively. At this stage, our goal was to focus on the identification of total Th1, Th2, and Th17 CD4+ T cells, which explains why we excluded these additional (subsetting) markers from the final antibody combination. However, the three former markers (CD195, CD294, and CD161) might be added in an extended version of the EuroFlow IMM TCD4 tube for further subsetting of the Th1, Th2, and Th17 populations, respectively.

Overall, we observed a high association between the chemokine receptor-based Th-cell phenotypes and both the *in vitro* cytokine secretion and the GEP patterns. For GEP assays, conventional qPCR was used because of the relatively low numbers of cells available from several of the 22 (highly purified) blood CD4+ T-cell subsets. The use of a more unbiased approach with a higher resolution ability (i.e., RNA-seq) would have been more appropriate to fully analyze the GEP of each specific CD4+ T-cell subset. However, the selected qPCR approach was sufficient to confirm the functional profile of the sorted CD4+ T-cell subsets based on a limited set of well-defined genes, which have been previously demonstrated to be associated with specific Th, Treg, and TFH profiles (1, 2, 5, 6, 8).

Thus, well-established Th2 and Th17 genes such as *IL4* and *IL4R* (in the absence of Th1 genes) and *CCR6* and *RORC*, respectively (1, 2), were expressed by phenotypically defined Th2 or Th17 cells. Even though the T-cell subsets sorted for the GEP assay had not been stimulated *in vitro*, some of them showed high expression of cytokine-related genes (e.g., cells with a Th2 phenotype overexpressed the *IL4* gene). These findings were supported by further *in vitro* stimulation assays, which found IL4+IL5 and IL17A production by T cells to be closely associated with Th2 or Th17 phenotypes, respectively. Of note, overexpression of the TNF gene (coding TNF-α protein) was found among Th17 cells in the GEP assay, which might be related with the relevance of TNF-α for the Th17 function, as previously described by others (52). Despite the reported association between CD183 expression and *in vitro* secretion of IFNγ, we only observed a partial association between both markers of Th1 cells, which was caused by downregulation of CD183 expression after *in vitro* stimulation of Th1 cells (22), potentially due to internalization of the CD183 molecule into the cytoplasm (46, 47). However, GEP confirmed the accurate phenotypic identification of Th1 cells in steady-state (non-cultured) blood based on CD183, as reflected by the typical overexpression of Th1-related genes (e.g., *RUNX3, EOMES*, and *HLX*) (53), which is in line with recent mass cytometry findings (29). Interestingly, we also found IL6 overexpression by Th1 cells; despite IL6 being a cytokine classically associated with Th2 differentiation that inhibits Th1 polarization (54), it has been widely recognized that actually IL6 is a highly pleiotropic cytokine, which play many different functional roles, from which its pro-inflammatory function—parallel to that of TNF-α (a Th1 cytokine)—is one of the most relevant.

Moreover, similar transcriptomics/phenotypic associations were also confirmed for other minor Th-cell subsets, such as Th1/Th17 and Th22 cells: mixed expression of Th1- and Th17-related genes by Th1/Th17 cells and overexpression of the AHR transcription factor as well as IL26 in Th22 cells (1, 5, 55, 56). Of note, Th22 cells also showed overexpression of the *IL13* gene, *IL13* being a key effector cytokine in immune responses against intracellular parasites and in the modulation of tumor cell growth and apoptosis (57, 58). In addition to the “classical” Th-cell subpopulations, based on the here proposed antibody combination, new Th subsets with unique patterns of expression of chemokine receptors were identified, such as CD183+CD194+CD196–CCR10– and CD183–CD194+CD196–CCR10+ CD4+ T cells with Th1- and Th22-associated GEP, respectively. Further studies using higher-resolution techniques for GEP (i.e., RNA-seq) are needed to elucidate the specific functions of these newly identified CD4+ T-cell subsets.

Since a significant overlap exists between classical Th populations and Th-like subsets of Tregs and TFH cells, precise identification and discrimination of the latter two CD4+ T-cell populations from classical Th cells become essential. For this purpose, the three additional CD25, CD127, and CD185 markers were included in the EuroFlow IMM TCD4 antibody combination for more accurate identification of Tregs and TFH cells, based on their typical CD127^−/lo^CD25^hi^ and CD185+ profiles (1, 2, 6, 43–45), respectively. Treg and TFH signatures of CD127^−/lo^CD25^hi^ and CD185+ CD4+ T cells were further supported here by GEP (6, 59). Thus, the former cells expressed the CTLA4 Treg-related gene, while the latter expressed *PDCD1*, among other TFH-associated genes. In turn, Tregs could be discriminated from Treg-like TFH based on their distinct expression profiles for CTLA4 and their differential expression of some genes (i.e., *IL17A*), despite their being clustered together in the heatmap shown in Figure 2D. Overexpression of IL17A here found in Tregs (vs. both Treg-like TFH and non-regulatory CD4+ T cells) is supported by previous reports (60) that demonstrated the production of this Th17-associated cytokine by human Tregs. Actually, also in line with previous data (61) multiple subsets of Th-like Tregs and TFH cells (30, 62) were identified based on the heterogeneous phenotypic profiles of both CD4+ T-cell populations for other chemokine receptors included in the EuroFlow IMM TCD4 tube. Other surface markers that identify terminally differentiated and memory Tregs, such as CD15s and CD39 (63, 64) were not selected because the corresponding Treg subsets are already identified in the proposed antibody panel, based on the expression pattern of maturation-associated markers.

The EuroFlow IMM TCD4 tube design was concluded by inclusion of the CD154 activation-associated marker and CD45. Compared with other activation markers evaluated, CD154 showed the closest association with *in vitro* T-cell activation. These results support previous findings (22, 65) but require further confirmation of its utility for immune monitoring in fresh (unstimulated) human blood in clinical settings of T-cell activation (e.g., vaccination and infection) in parallel to other potentially informative activation markers, such as CD278, CD279, Ki-67, and HLADR.

Overall, with the proposed antibody combination, ≥89 clearly distinct CD4+ T-cell subsets were identified in human blood with a relatively high degree of correlation among experts and between automated and expert-based manual data analysis. Our findings also highlight the potential benefit of database-guided automated identification and quantitation of classical Th, Treg, and TFH-cell populations vs. expert-based manual analysis to reduce operator-related variability, in line with previous findings (36, 66). In addition, automated data analysis is faster and less labor-intensive. However, automated gating also showed some limitations, particularly with regard to the identification of minimally represented cell populations (<0.1%), subsets of TFH cells that displayed heterogeneous (from dim to strong) expression profiles for the gating markers (e.g., CD194 and CD196), and cross-contamination among phenotypically similar (minor) cell populations (e.g., Th2 cells and CD194+ Tregs). Further studies in which a higher number of events are measured, together with the use of automated gating on heterogeneously (continuous) expressed markers based on EuroFlow maturation tools (34) and a specific warning for critical cell populations that are more prone to cross-contamination, will probably overcome these limitations.

Altogether, these findings indicate that the EuroFlow IMM TCD4 tube provides the basis for robust and accurate identification and enumeration of many (≥89) different CD4+ T-cell populations in normal blood, as demonstrated here via highly reproducible multicenter results. For translation into clinical settings, age-related reference values are essential, because CD4+ T cells and their subsets in blood change throughout life: total T cells and CD4+ T cells reached their maximum levels during the first 2 years of life, gradually decreasing thereafter, until adulthood (18 years). These kinetics are mostly due to an early increase in naïve T cells caused by massive T-cell production during the first years of life (67, 68) while in adulthood, an overall increase in memory/effector cells (e.g., Th subsets) at the expense of a lower naïve CD4+ T-cell production was found. In line with previous studies (44), Th2 and Treg counts were higher in CB in the absence at birth of virtually all other Th and TFH cells. Subsequently, an increase of TFH and (mostly) Treg counts was observed up to 2 years of age, followed by higher memory B-cell counts in children aged 2–4 years (69). This probably reflects the need for sufficient numbers of TFH cells to enable immunoglobulin class switching (6) and the modulation of TFH-cell activity by increased Tregs. Similarly, Th2, Th17, and to a lesser extent Th1 cells were also increased at 2 years, probably reflecting priming of classical Th-cell responses conditioned by multiple encounters with pathogens and vaccines. Transition into infancy was associated with a dramatic decrease in naïve, Treg, and Th2 cells until adulthood, when Th1-, Th17-, and TFH-cell counts started to increase. Both Th1/Th17 and Th22 T cells (undetectable in CB) showed a progressive increase since infancy toward 40–60 years, slightly decreasing thereafter.

Interestingly, preliminary results based on the evaluation of EuroFlow IMM TCD4 tube in patients with MBL, SM (two early cancer models), and CVID showed distinct patterns of alteration of CD4 T-cell subsets in blood. Thus, a significant increase of naïve CD4+ T cells was found in patients with SM, while a generalized decrease of virtually all blood CD4+ T-cell subsets was found in CVID, with normal values among MBL subjects. Altogether, these findings support the potential utility of the here described EuroFlow IMM TCD4 tube for the identification and characterization of potentially altered immune profiles in blood of patients with different disease conditions. However, in such studies, variables such as age and the antigenic environment might play a critical role in selecting appropriate reference samples (e.g., from age-matched subjects living in the same geographic region of that of the patients under evaluation).

Altogether, these findings confirm the enormous kinetics observed in blood during life for most lymphoid subsets with unique profiles for the distinct memory CD4+ Th-cell populations, setting the basis for future immune monitoring in different disease and treatment conditions.

## Data Availability Statement

The raw data supporting the conclusions of this article are available here: https://biodata.usal.es/share.cgi?ssid=0286vNK.

## Ethics Statement

The studies involving human participants were reviewed and approved by the Ethics Committee of the University Hospital of Salamanca/IBSAL (Salamanca, Spain). Written informed consent to participate in this study was provided by the participants' legal guardian/next of kin.

## Author Contributions

JA, AO, JD, FM, CE, and VC contributed to the conception and design of the study. VB, JA, MP-A, PB, AH-D, DD, SC, EB, and AM performed the flow cytometry and/or the *in vitro* stimulation assays and the analysis of the obtained data. MJ-A, VB, and JA performed the experiments and data analysis for the gene expression profile assay. GG, AH-D, SC, JG, JA, CP, A-MB, and VB performed the construction and validation of the TCD4 tube database for automated analysis. AR, SA-M, IG-A, and AM collected samples from healthy donors for the study and provided their relevant clinical information. VB, JA, AO, and JD wrote the manuscript. All authors contributed to the manuscript revision and read and approved the submitted version.

### Conflict of Interest

JD and AO report to be chairmen of the EuroFlow scientific foundation, which receives royalties from licensed patents, which are collectively owned by the participants of the EuroFlow Foundation. These royalties are exclusively used for continuation of the EuroFlow collaboration and sustainability of the EuroFlow consortium. JD and AO report an Educational Services Agreement from BD Biosciences (San José, CA) and a Scientific Advisor Agreement with Cytognos; all related fees and honoraria are for the involved university departments at Leiden University Medical Center and University of Salamanca. GG and AH-D are employees of Cytognos (Salamanca, Spain). The remaining authors declare that the research was conducted in the absence of any commercial or financial relationships that could be construed as a potential conflict of interest.
