# Supplementary material for: Age Distribution of Multiple Functionally Relevant Subsets of CD4+ T Cells in Human Blood Using a Standardized and Validated 14-Color EuroFlow Immune Monitoring Tube
Source: Front Immunol. 2020 Feb 27;11:166. doi: 10.3389/fimmu.2020.00166 (PMC7056740; doi:10.3389/fimmu.2020.00166)
Supplement: Supplementary file 1 [file Presentation_1.PPTX]

## Slide 1
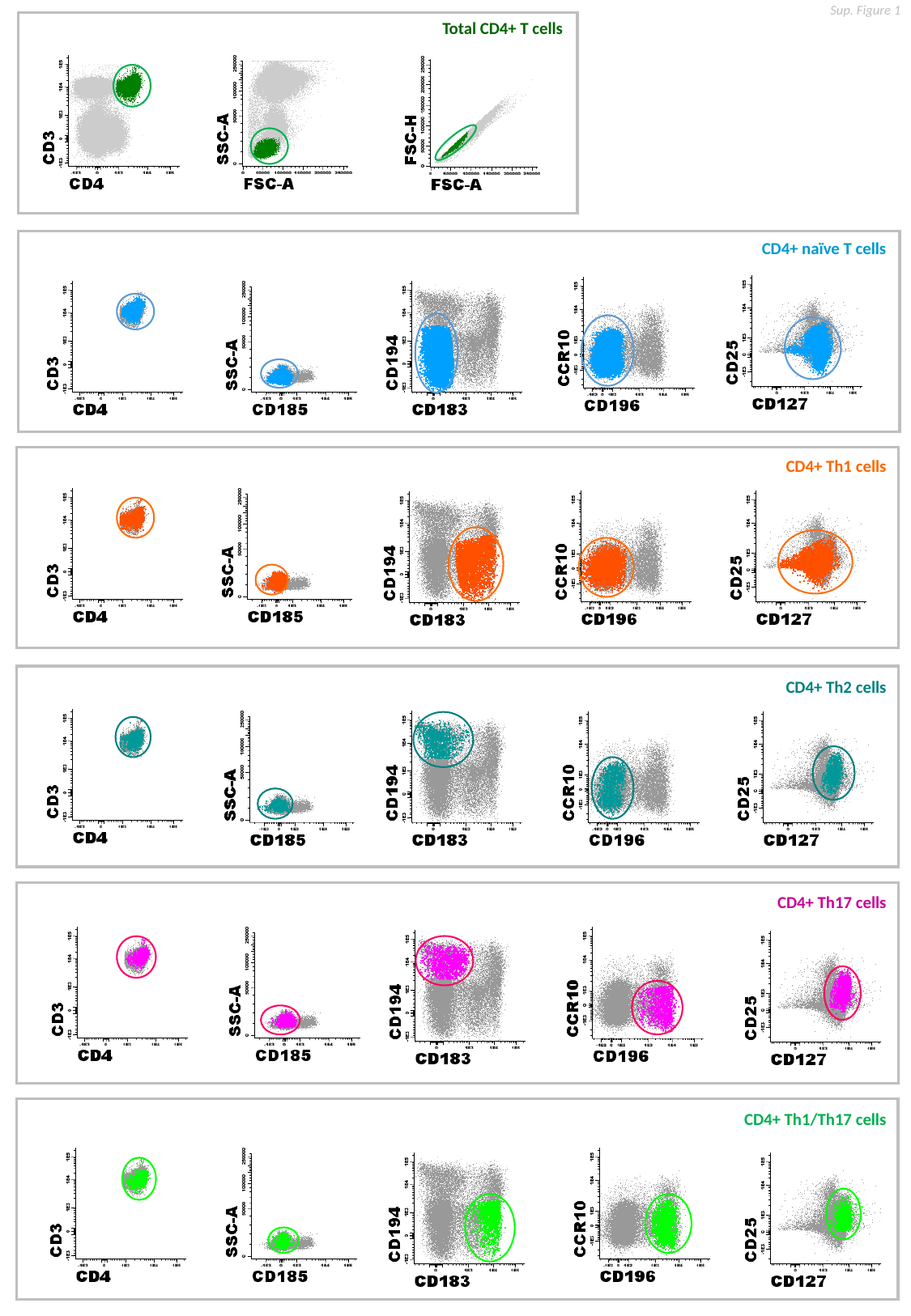

Sup. Figure 1
Total CD4+ T cells
CD4+ naïve T cells
CD4+ Th1 cells
CD4+ Th2 cells
CD4+ Th17 cells
CD4+ Th1/Th17 cells

## Slide 2
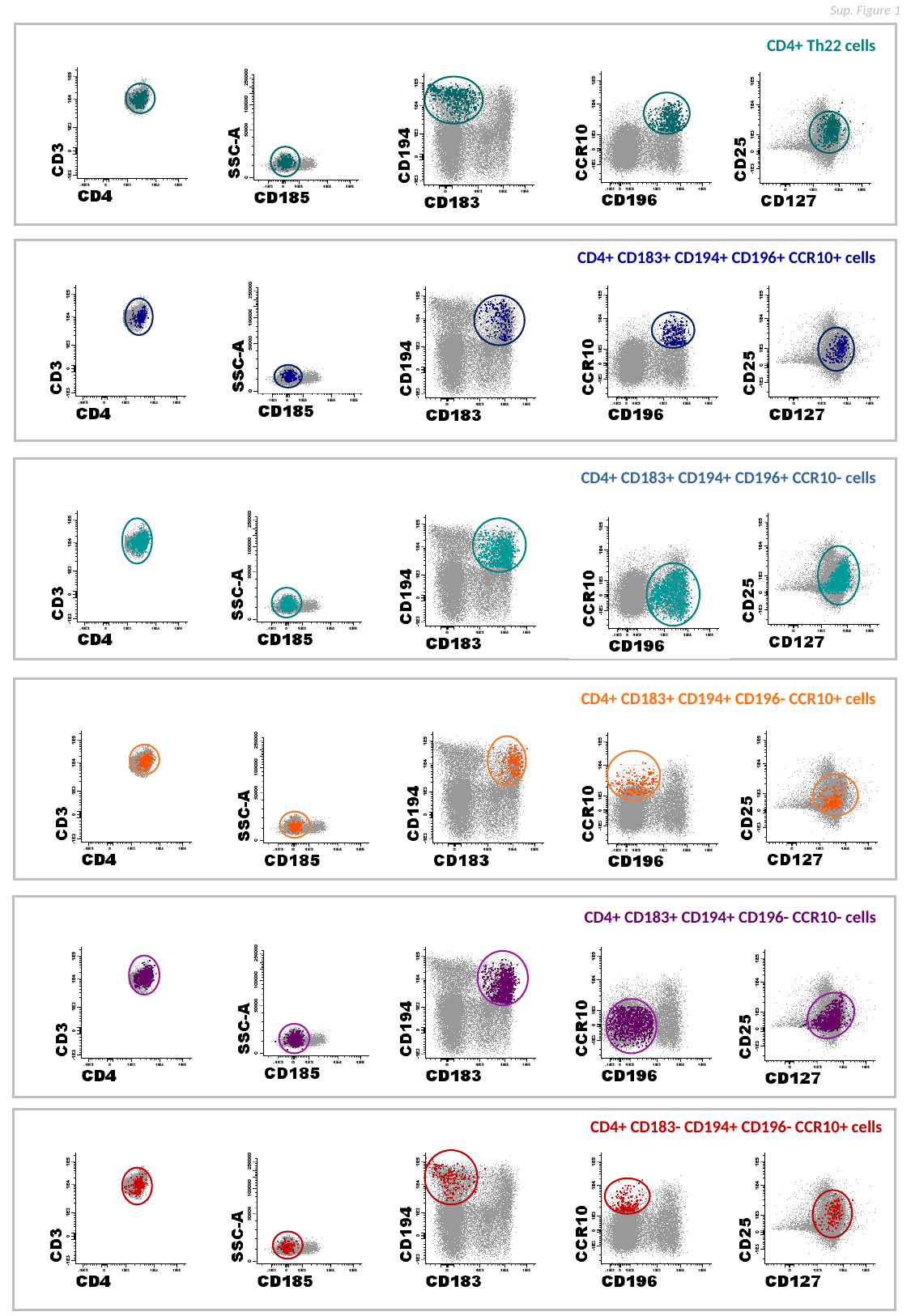

Sup. Figure 1
v
CD4+ Th22 cells
CD4+ CD183+ CD194+ CD196+ CCR10+ cells
CD4+ CD183+ CD194+ CD196+ CCR10- cells
CD4+ CD183+ CD194+ CD196- CCR10+ cells
CD4+ CD183+ CD194+ CD196- CCR10- cells
CD4+ CD183- CD194+ CD196- CCR10+ cells

## Slide 3
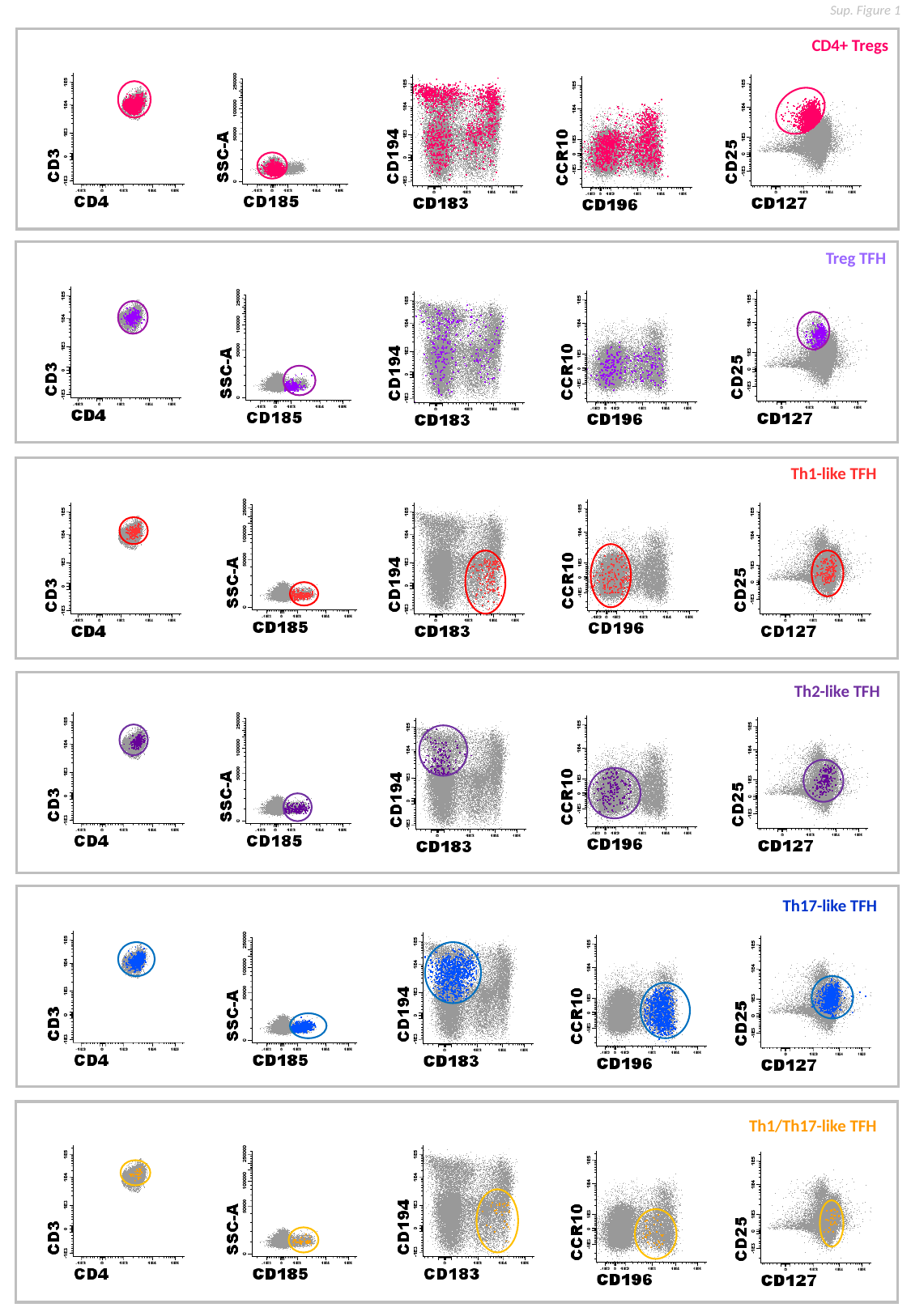

Sup. Figure 1
CD4+ Tregs
Treg TFH
Th1-like TFH
Th2-like TFH
Th17-like TFH
Th1/Th17-like TFH

## Slide 4
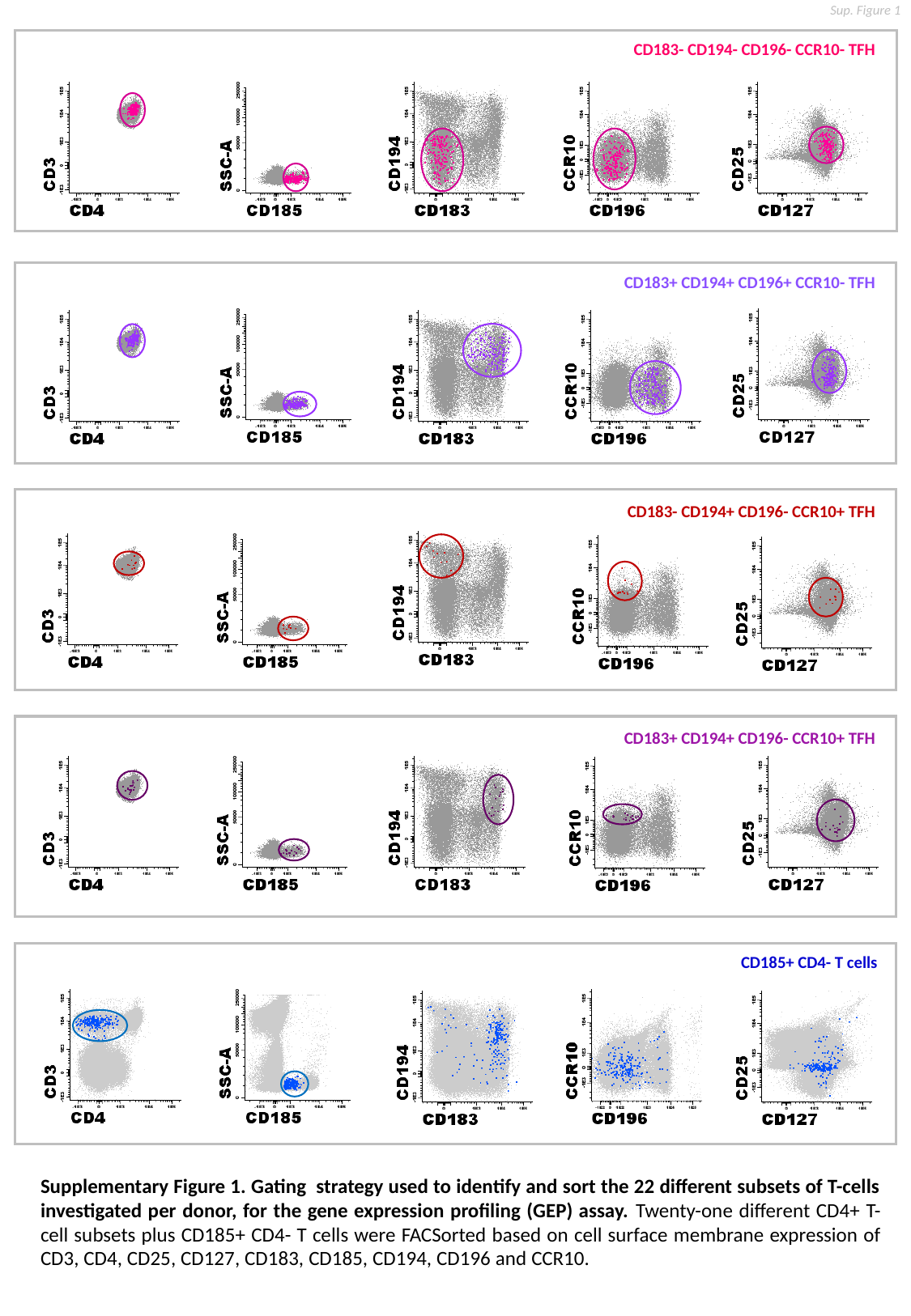

Sup. Figure 1
CD183- CD194- CD196- CCR10- TFH
CD183+ CD194+ CD196+ CCR10- TFH
CD183- CD194+ CD196- CCR10+ TFH
CD183+ CD194+ CD196- CCR10+ TFH
CD185+ CD4- T cells
Supplementary Figure 1. Gating  strategy used to identify and sort the 22 different subsets of T-cells investigated per donor, for the gene expression profiling (GEP) assay. Twenty-one different CD4+ T-cell subsets plus CD185+ CD4- T cells were FACSorted based on cell surface membrane expression of CD3, CD4, CD25, CD127, CD183, CD185, CD194, CD196 and CCR10.
