# Supplementary material for: Age Distribution of Multiple Functionally Relevant Subsets of CD4+ T Cells in Human Blood Using a Standardized and Validated 14-Color EuroFlow Immune Monitoring Tube
Source: Front Immunol. 2020 Feb 27;11:166. doi: 10.3389/fimmu.2020.00166 (PMC7056740; doi:10.3389/fimmu.2020.00166)
Supplement: Supplementary file 2 [file Presentation_2.PPTX]

## Slide 1
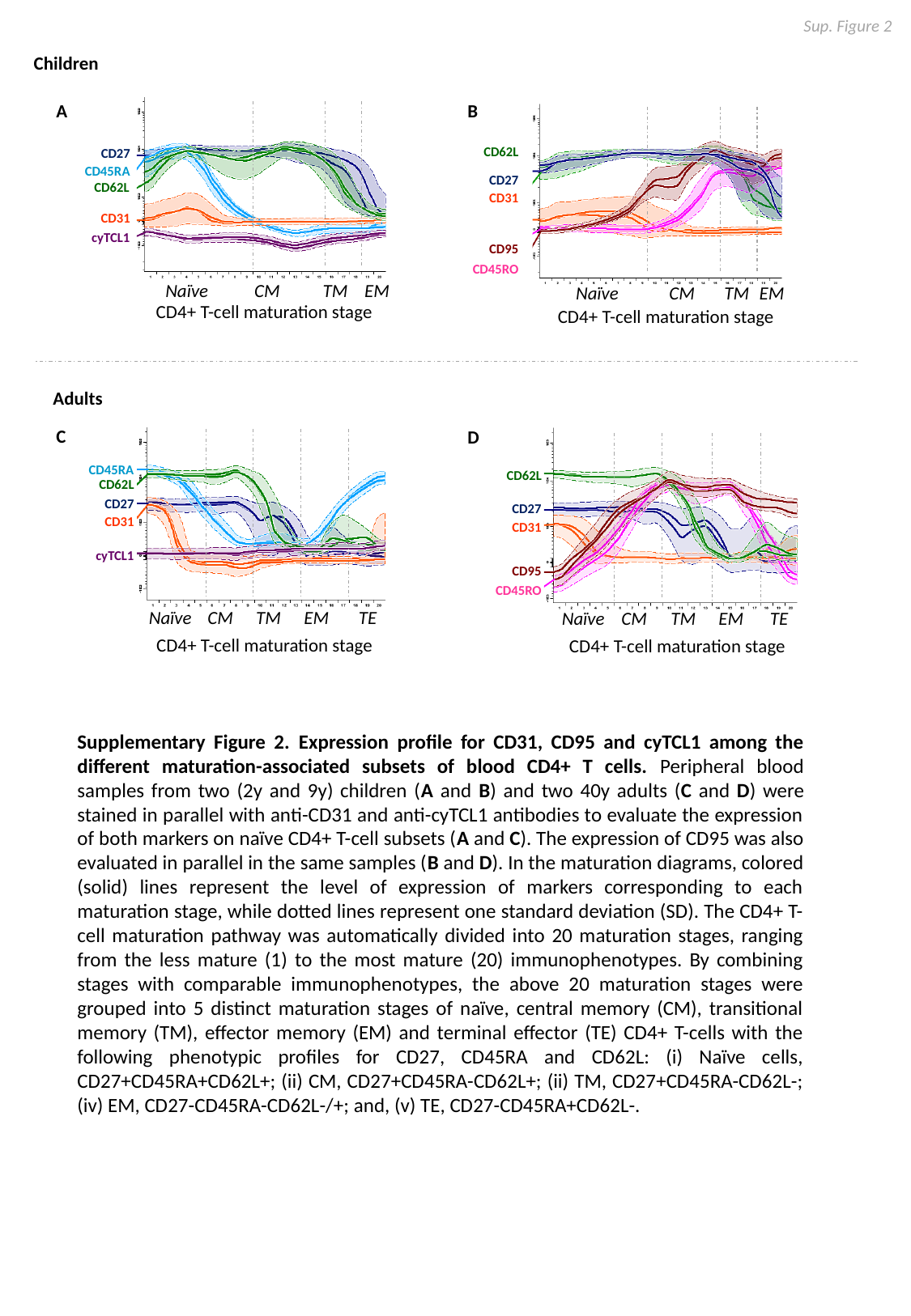

Sup. Figure 2
Children
A
B
CD62L
CD27
CD45RA
CD27
CD62L
CD31
CD31
cyTCL1
CD95
CD45RO
Naïve
CM
TM
EM
Naïve
CM
TM
EM
CD4+ T-cell maturation stage
CD4+ T-cell maturation stage
Adults
C
D
CD45RA
CD62L
CD62L
CD27
CD27
CD31
CD31
cyTCL1
CD95
CD45RO
Naïve
CM
TM
EM
TE
Naïve
CM
TM
EM
TE
CD4+ T-cell maturation stage
CD4+ T-cell maturation stage
Supplementary Figure 2. Expression profile for CD31, CD95 and cyTCL1 among the different maturation-associated subsets of blood CD4+ T cells. Peripheral blood samples from two (2y and 9y) children (A and B) and two 40y adults (C and D) were stained in parallel with anti-CD31 and anti-cyTCL1 antibodies to evaluate the expression of both markers on naïve CD4+ T-cell subsets (A and C). The expression of CD95 was also evaluated in parallel in the same samples (B and D). In the maturation diagrams, colored (solid) lines represent the level of expression of markers corresponding to each maturation stage, while dotted lines represent one standard deviation (SD). The CD4+ T-cell maturation pathway was automatically divided into 20 maturation stages, ranging from the less mature (1) to the most mature (20) immunophenotypes. By combining stages with comparable immunophenotypes, the above 20 maturation stages were grouped into 5 distinct maturation stages of naïve, central memory (CM), transitional memory (TM), effector memory (EM) and terminal effector (TE) CD4+ T-cells with the following phenotypic profiles for CD27, CD45RA and CD62L: (i) Naïve cells, CD27+CD45RA+CD62L+; (ii) CM, CD27+CD45RA-CD62L+; (ii) TM, CD27+CD45RA-CD62L-; (iv) EM, CD27-CD45RA-CD62L-/+; and, (v) TE, CD27-CD45RA+CD62L-.
