# Supplementary material for: Age Distribution of Multiple Functionally Relevant Subsets of CD4+ T Cells in Human Blood Using a Standardized and Validated 14-Color EuroFlow Immune Monitoring Tube
Source: Front Immunol. 2020 Feb 27;11:166. doi: 10.3389/fimmu.2020.00166 (PMC7056740; doi:10.3389/fimmu.2020.00166)
Supplement: Supplementary file 3 [file Presentation_3.PPTX]

## Slide 1
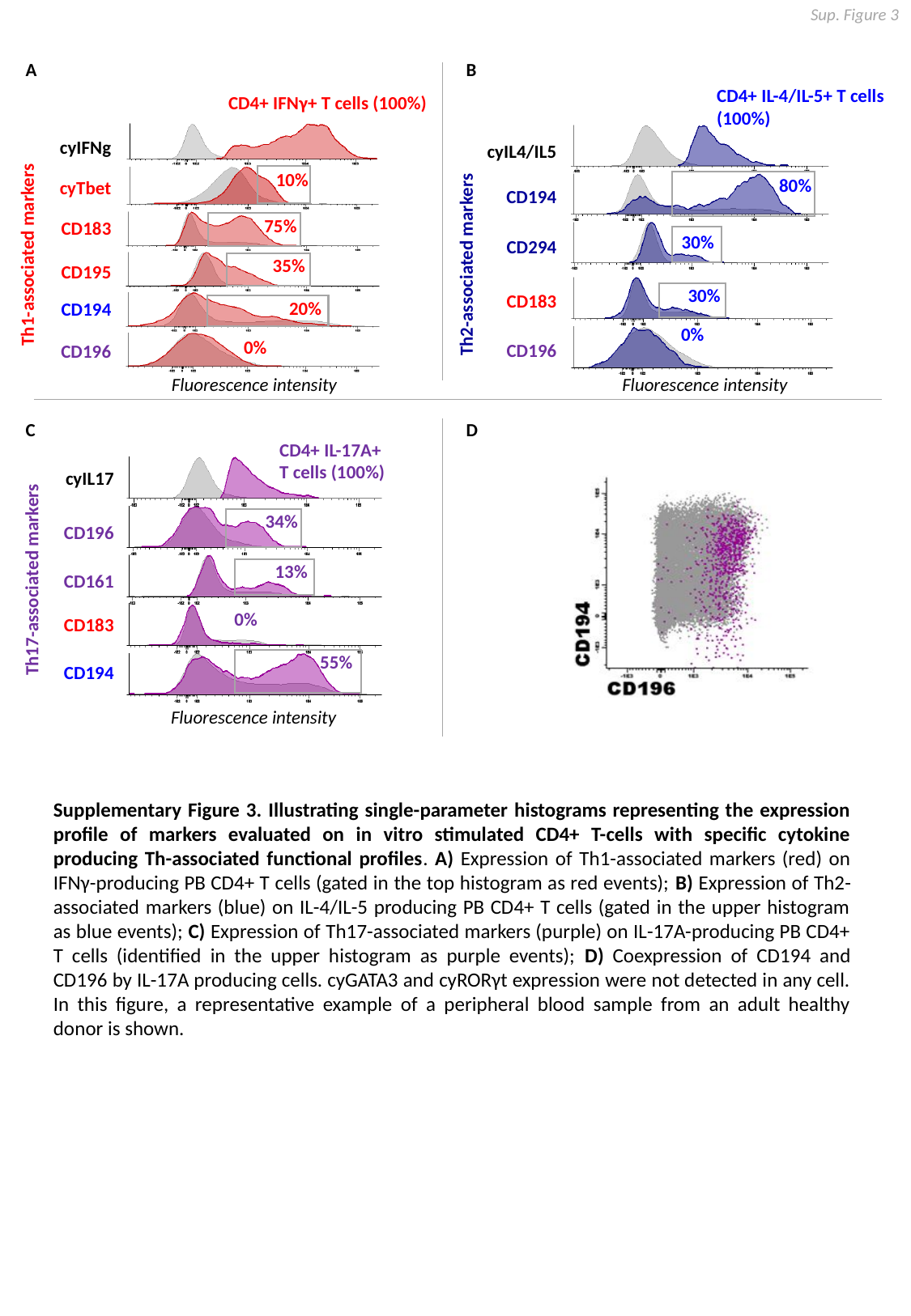

Sup. Figure 3
A
B
CD4+ IL-4/IL-5+ T cells
(100%)
cyIL4/IL5
80%
CD194
30%
CD294
Th2-associated markers
30%
CD183
0%
CD196
CD4+ IFNγ+ T cells (100%)
cyIFNg
10%
cyTbet
75%
CD183
Th1-associated markers
35%
CD195
20%
CD194
0%
CD196
Fluorescence intensity
Fluorescence intensity
C
D
CD4+ IL-17A+ T cells (100%)
cyIL17
34%
CD196
13%
Th17-associated markers
CD161
0%
CD183
55%
CD194
Fluorescence intensity
Supplementary Figure 3. Illustrating single-parameter histograms representing the expression profile of markers evaluated on in vitro stimulated CD4+ T-cells with specific cytokine producing Th-associated functional profiles. A) Expression of Th1-associated markers (red) on IFNγ-producing PB CD4+ T cells (gated in the top histogram as red events); B) Expression of Th2-associated markers (blue) on IL-4/IL-5 producing PB CD4+ T cells (gated in the upper histogram as blue events); C) Expression of Th17-associated markers (purple) on IL-17A-producing PB CD4+ T cells (identified in the upper histogram as purple events); D) Coexpression of CD194 and CD196 by IL-17A producing cells. cyGATA3 and cyRORγt expression were not detected in any cell. In this figure, a representative example of a peripheral blood sample from an adult healthy donor is shown.
