# Supplementary material for: Age Distribution of Multiple Functionally Relevant Subsets of CD4+ T Cells in Human Blood Using a Standardized and Validated 14-Color EuroFlow Immune Monitoring Tube
Source: Front Immunol. 2020 Feb 27;11:166. doi: 10.3389/fimmu.2020.00166 (PMC7056740; doi:10.3389/fimmu.2020.00166)
Supplement: Supplementary file 4 [file Presentation_4.PPTX]

## Slide 1
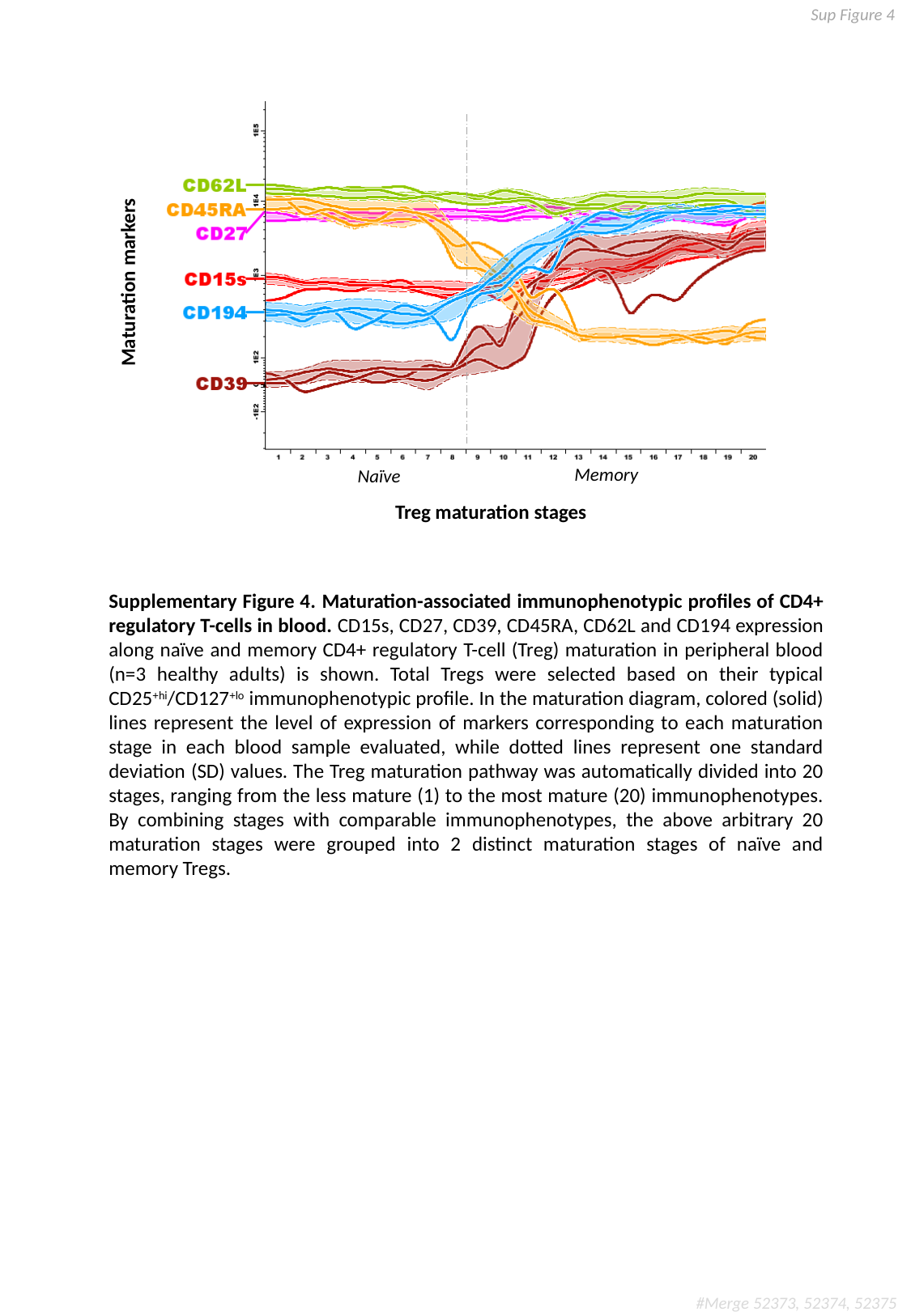

Sup Figure 4
Maturation markers
Memory
Naïve
Treg maturation stages
Supplementary Figure 4. Maturation-associated immunophenotypic profiles of CD4+ regulatory T-cells in blood. CD15s, CD27, CD39, CD45RA, CD62L and CD194 expression along naïve and memory CD4+ regulatory T-cell (Treg) maturation in peripheral blood (n=3 healthy adults) is shown. Total Tregs were selected based on their typical CD25+hi/CD127+lo immunophenotypic profile. In the maturation diagram, colored (solid) lines represent the level of expression of markers corresponding to each maturation stage in each blood sample evaluated, while dotted lines represent one standard deviation (SD) values. The Treg maturation pathway was automatically divided into 20 stages, ranging from the less mature (1) to the most mature (20) immunophenotypes. By combining stages with comparable immunophenotypes, the above arbitrary 20 maturation stages were grouped into 2 distinct maturation stages of naïve and memory Tregs.
#Merge 52373, 52374, 52375
