# Supplementary material for: Age Distribution of Multiple Functionally Relevant Subsets of CD4+ T Cells in Human Blood Using a Standardized and Validated 14-Color EuroFlow Immune Monitoring Tube
Source: Front Immunol. 2020 Feb 27;11:166. doi: 10.3389/fimmu.2020.00166 (PMC7056740; doi:10.3389/fimmu.2020.00166)
Supplement: Supplementary file 5 [file Presentation_5.PPTX]

## Slide 1
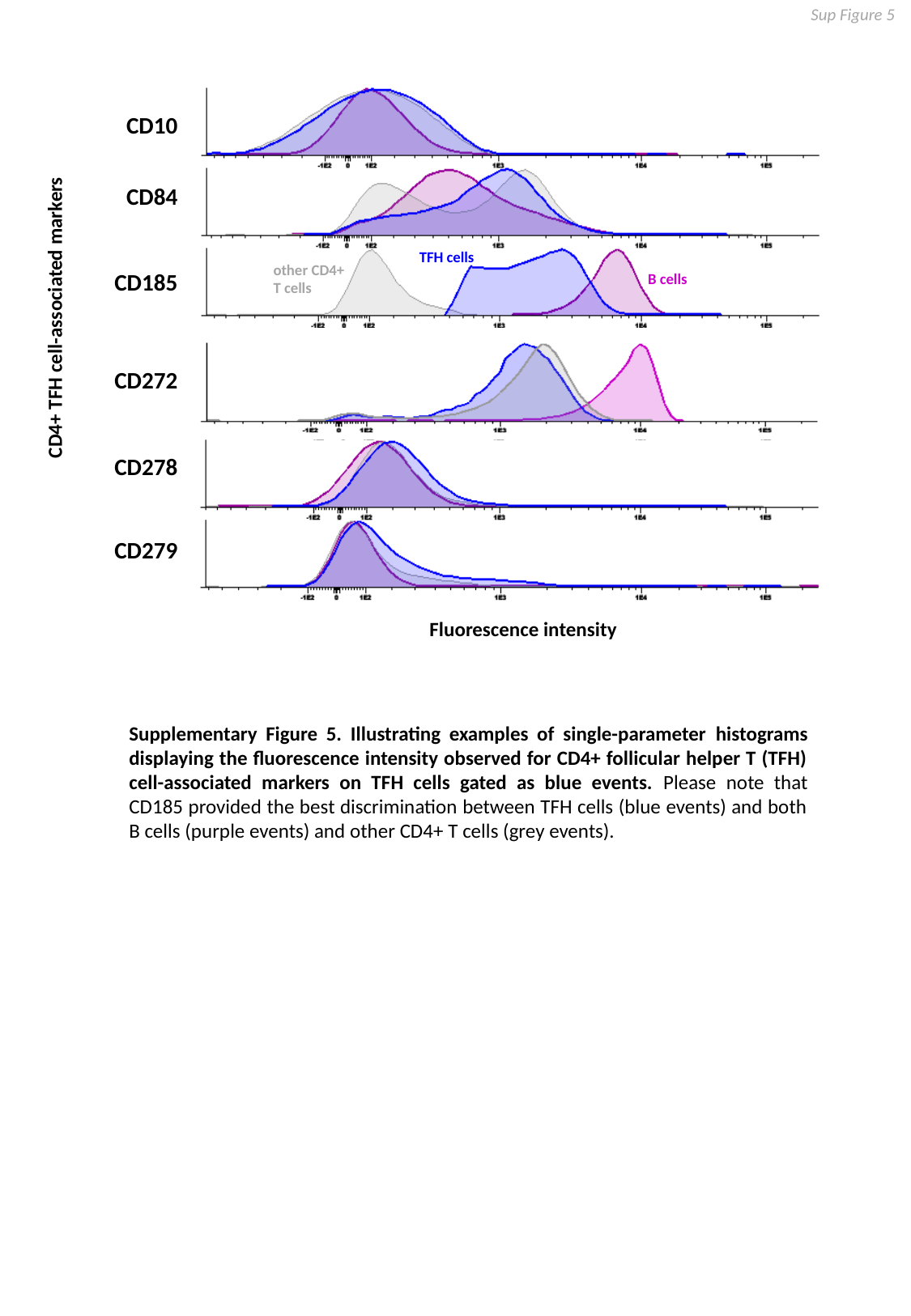

Sup Figure 5
CD10
CD84
CD185
CD272
CD278
CD279
TFH cells
other CD4+ T cells
B cells
CD4+ TFH cell-associated markers
Fluorescence intensity
Supplementary Figure 5. Illustrating examples of single-parameter histograms displaying the fluorescence intensity observed for CD4+ follicular helper T (TFH) cell-associated markers on TFH cells gated as blue events. Please note that CD185 provided the best discrimination between TFH cells (blue events) and both B cells (purple events) and other CD4+ T cells (grey events).
