# Supplementary material for: Age Distribution of Multiple Functionally Relevant Subsets of CD4+ T Cells in Human Blood Using a Standardized and Validated 14-Color EuroFlow Immune Monitoring Tube
Source: Front Immunol. 2020 Feb 27;11:166. doi: 10.3389/fimmu.2020.00166 (PMC7056740; doi:10.3389/fimmu.2020.00166)
Supplement: Supplementary file 6 [file Presentation_6.PPTX]

## Slide 1
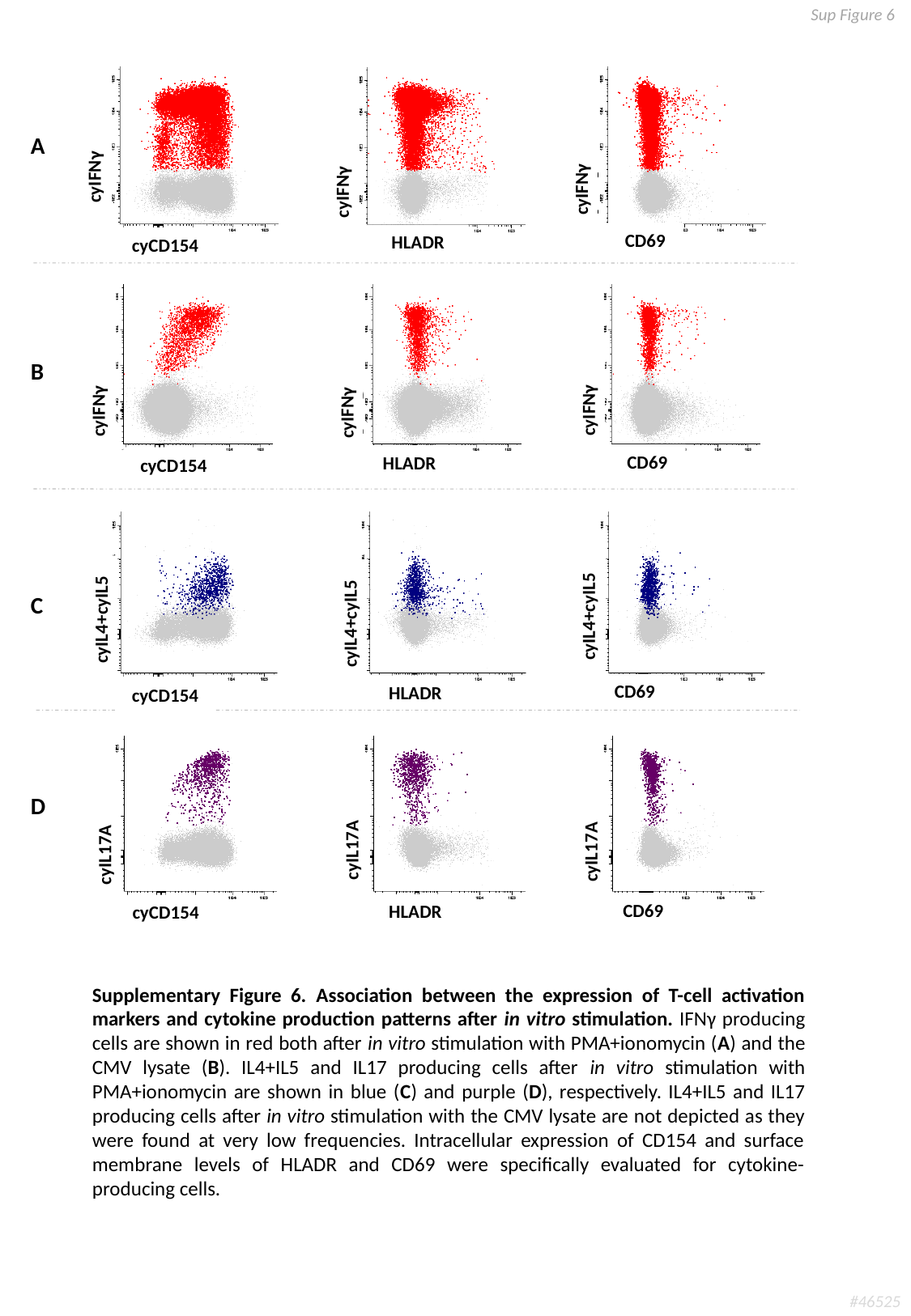

Sup Figure 6
A
cyIFNγ
cyIFNγ
cyIFNγ
CD69
HLADR
cyCD154
B
cyIFNγ
cyIFNγ
cyIFNγ
CD69
HLADR
cyCD154
C
cyIL4+cyIL5
cyIL4+cyIL5
cyIL4+cyIL5
CD69
cyCD154
HLADR
D
cyIL17A
cyIL17A
cyIL17A
cyCD154
CD69
HLADR
Supplementary Figure 6. Association between the expression of T-cell activation markers and cytokine production patterns after in vitro stimulation. IFNγ producing cells are shown in red both after in vitro stimulation with PMA+ionomycin (A) and the CMV lysate (B). IL4+IL5 and IL17 producing cells after in vitro stimulation with PMA+ionomycin are shown in blue (C) and purple (D), respectively. IL4+IL5 and IL17 producing cells after in vitro stimulation with the CMV lysate are not depicted as they were found at very low frequencies. Intracellular expression of CD154 and surface membrane levels of HLADR and CD69 were specifically evaluated for cytokine-producing cells.
#46525
