# Supplementary material for: Age Distribution of Multiple Functionally Relevant Subsets of CD4+ T Cells in Human Blood Using a Standardized and Validated 14-Color EuroFlow Immune Monitoring Tube
Source: Front Immunol. 2020 Feb 27;11:166. doi: 10.3389/fimmu.2020.00166 (PMC7056740; doi:10.3389/fimmu.2020.00166)
Supplement: Supplementary file 7 [file Presentation_7.PPTX]

## Slide 1
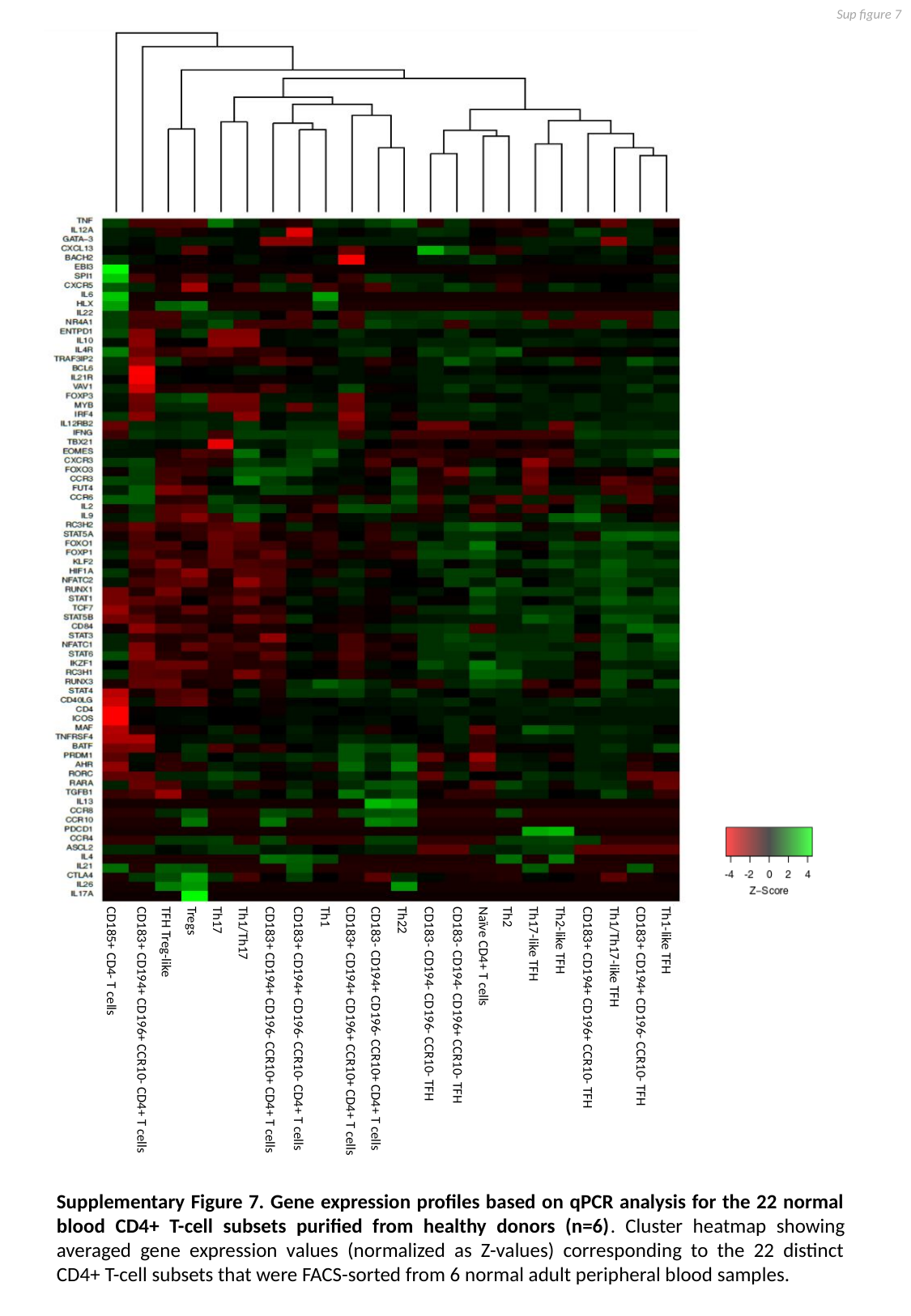

Sup figure 7
Th1-like TFH
CD185+ CD4- T cells
CD183+ CD194+ CD196+ CCR10- CD4+ T cells
TFH Treg-like
Th1/Th17
Th1
CD183+ CD194+ CD196+ CCR10+ CD4+ T cells
CD183- CD194+ CD196- CCR10+ CD4+ T cells
CD183- CD194- CD196+ CCR10- TFH
Naïve CD4+ T cells
Th2
Th2-like TFH
Th1/Th17-like TFH
CD183+ CD194+ CD196- CCR10- TFH
Tregs
Th17
CD183+ CD194+ CD196- CCR10+ CD4+ T cells
CD183+ CD194+ CD196- CCR10- CD4+ T cells
Th22
CD183- CD194- CD196- CCR10- TFH
Th17-like TFH
CD183+ CD194+ CD196+ CCR10- TFH
Supplementary Figure 7. Gene expression profiles based on qPCR analysis for the 22 normal blood CD4+ T-cell subsets purified from healthy donors (n=6). Cluster heatmap showing averaged gene expression values (normalized as Z-values) corresponding to the 22 distinct CD4+ T-cell subsets that were FACS-sorted from 6 normal adult peripheral blood samples.
