# Supplementary material for: Age Distribution of Multiple Functionally Relevant Subsets of CD4+ T Cells in Human Blood Using a Standardized and Validated 14-Color EuroFlow Immune Monitoring Tube
Source: Front Immunol. 2020 Feb 27;11:166. doi: 10.3389/fimmu.2020.00166 (PMC7056740; doi:10.3389/fimmu.2020.00166)
Supplement: Supplementary file 8 [file Presentation_8.pptx]

## Slide 1
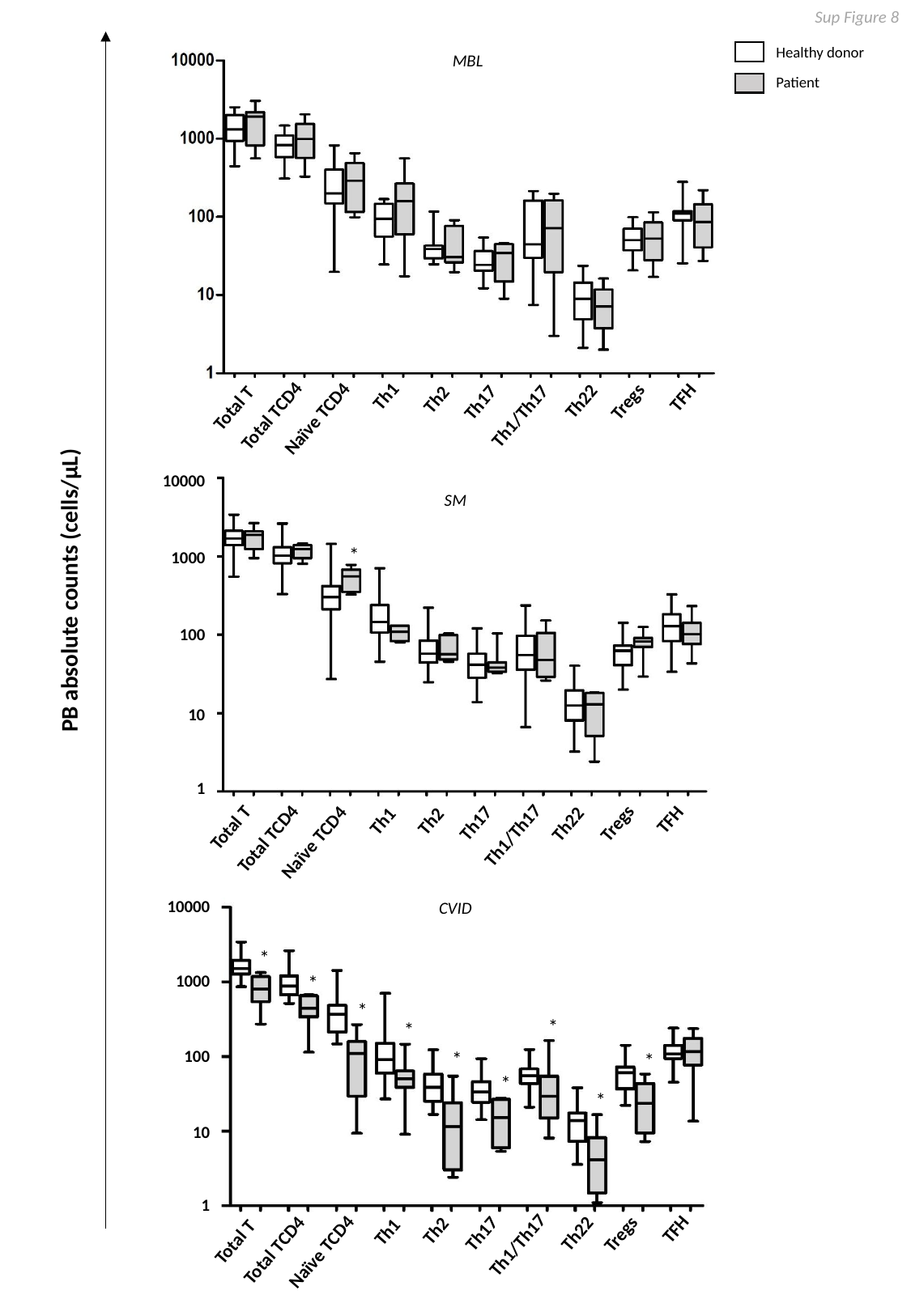

Sup Figure 8
Healthy donor
MBL
Patient
Th1
TFH
Th2
Th22
Tregs
Th17
Total T
Total TCD4
Th1/Th17
Naïve TCD4
10000
1000
100
10
1
SM
*
PB absolute counts (cells/µL)
TFH
Th2
Th1
Tregs
Th17
Th22
Total T
Th1/Th17
Total TCD4
Naïve TCD4
10000
1000
100
10
1
CVID
*
*
*
*
*
*
*
*
*
TFH
Th2
Th1
Th17
Th22
Tregs
Total T
Th1/Th17
Total TCD4
Naïve TCD4

## Slide 2
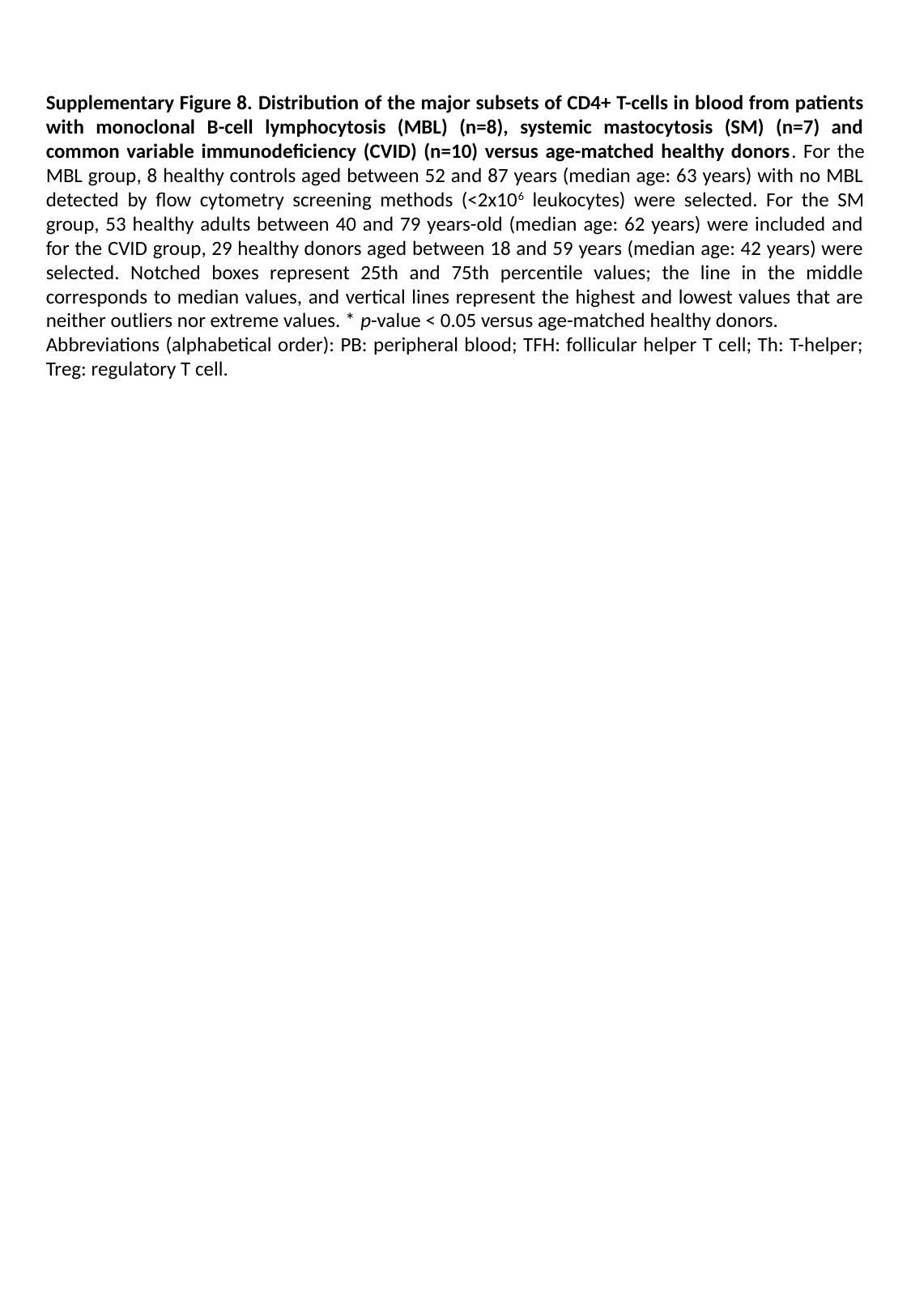

Supplementary Figure 8. Distribution of the major subsets of CD4+ T-cells in blood from patients with monoclonal B-cell lymphocytosis (MBL) (n=8), systemic mastocytosis (SM) (n=7) and common variable immunodeficiency (CVID) (n=10) versus age-matched healthy donors. For the MBL group, 8 healthy controls aged between 52 and 87 years (median age: 63 years) with no MBL detected by flow cytometry screening methods (<2x10^6^ leukocytes) were selected. For the SM group, 53 healthy adults between 40 and 79 years-old (median age: 62 years) were included and for the CVID group, 29 healthy donors aged between 18 and 59 years (median age: 42 years) were selected. Notched boxes represent 25th and 75th percentile values; the line in the middle corresponds to median values, and vertical lines represent the highest and lowest values that are neither outliers nor extreme values. * p-value < 0.05 versus age-matched healthy donors.
Abbreviations (alphabetical order): PB: peripheral blood; TFH: follicular helper T cell; Th: T-helper; Treg: regulatory T cell.
