# Supplementary material for: Age Distribution of Multiple Functionally Relevant Subsets of CD4+ T Cells in Human Blood Using a Standardized and Validated 14-Color EuroFlow Immune Monitoring Tube
Source: Front Immunol. 2020 Feb 27;11:166. doi: 10.3389/fimmu.2020.00166 (PMC7056740; doi:10.3389/fimmu.2020.00166)
Supplement: Supplementary file 9 [file Data_Sheet_1.docx]

**Supplementary Table 1 -** Monoclonal antibodies evaluated for the design and construction of the EuroFlow IMM TCD4 tube.

| **Marker** | **Clone** | **Fluorochrome** | **Source** |  | **Catalogue number** | **Aim** | **Marker** | **Clone** | **Fluorochrome** | **Source** | **Catalogue number** | **Aim** |
| --- | --- | --- | --- | --- | --- | --- | --- | --- | --- | --- | --- | --- |
| CD3 | SK7 | PerCPcy5.5 | BD |  | 332771 | GIT | CD183 | 1C6/CXCR3 | APC | BD | 561732 | Th |
|  | SK7 | BV786 | BD |  | 563800 |  |  | 1C6/CXCR3 | BV421 | BD | 562558 |  |
| CD4 | SK3 | BV510 | BD |  | 562970 | GIT |  | 1C6/CXCR3 | PE | BD | 557185 |  |
|  | SK3 | BV711 | BD |  | 563028 |  | CD185 | REA103 | APC | Miltenyi | 130-098-422 | TFH |
|  | SK3 | APCH7 | BD |  | 641398 |  |  | 51505 | APC | R&D Systems | FAB190A-100 |  |
|  |  |  |  |  |  |  |  | J252D4 | APC | Immunostep | custom |  |
|  |  |  |  |  |  |  |  | MU5UBEE | APC | eBioscience | 17-9185-41 |  |
| CD5 | L17F12 | PerCPcy5 | BD |  | 341089 | GIT | CD194 | L291H4 | PE-Cy7 | Biolegend | 359410 | Th |
| CD7 | M-T701 | BV510 | BD |  | 563650 | GIT | CD195 | 2D7/CCR5 | FITC | BD | 555992 | Th |
| CD8 | RPA-T8 | PE-CF594 | BD |  | 562282 | GIT | CD196 | 11A9 | PerCPcy5.5 | BD | 560621 | Th |
| CD10 | HI10a | PE-Cy7 | BD |  | 341092 | TFH |  | 11A9 | PE-CF594 | BD | 564816 |  |
| CD15s | CSLEX1 | AF647 | BD |  | 563526 | Tregs | CD197 | 150503 | FITC | R&D Systems | FAB197F | MM |
| CD25 | 2A3 | FITC | BD |  | 347643 | Tregs & AM |  | 150503 | PE | R&D Systems | FAB197P |  |
|  | 4E3 | VioBright-FITC | Miltenyi |  | 130-104-274 |  | CD272 | J168-540 | PE-CF594 | BD | 564801 | TFH |
|  | M-A251 | PE-Cy7 | BD |  | 557741 |  | CD278 | DX29 | BV650 | BD | 563832 | TFH |
| CD27 | M-T271 | BV421 | BD |  | 562513 | MM | CD279 | MIH4 | PE | BD | 557946 | TFH |
|  | O323 | BV510 | Biolegend |  | 302836 |  |  | EH12.1 | PE-Cy7 | BD | 561272 |  |
| CD28 | CD28.2 | PerCPcy5 | Biolegend |  | 302922 | MM | CD294 | BM16 | FITC | BD | 561659 | Th |
| CD31 | MEM-05 | PE | Exbio |  | 1P-273-T100 | MM | CCR10 | 1B5 | BV421 | BD | 564770 | Th |
| CD39 | TU66 | PE-CF594 | BD |  | 563678 | Tregs |  | 1B5 | PerCPcy5.5 | BD | 564772 |  |
|  | TU66 | PE | BD |  | 555464 |  | cyFoxP3ᵟ | 236A/E7 | PE | eBioscience | 12-4777 | Tregs |
| CD45 | HI30 | AF700 | BD |  | 560566 | LI | cyGATA3^δ^ | TWAJ | PE-Cy7 | eBioscience | 25-9966-41 | Th |
| CD45RA | HI100 | BV480 | BD |  | 100656 | MM | HLADR | L243 | FITC | BD | 347363 | AM |
|  | HI100 | BV510 | BD |  | 563031 |  |  | G46-6 | PE-CF594 | BD | 562304 |  |
| CD45RO | UCHL1 | APCH7 | BD |  | 561137 | MM |  | L243 | PerCPcy5.5 | BD | 339194 |  |
| CD57 | NK-1 | APC | BD |  | 560845 | MM | cyIFNγ | B27 | APC | BD | 554702 | Th |
|  | NK-1 | BV421 | BD |  | 563896 |  |  | B27 | AF700 | BD | 561024 |  |
|  | HNK1 | FITC | BD |  | 333169 |  | cyIL17A | N49-653 | AF647 | BD | 560490 | Th |
| CD62L | DREG-56 | BV605 | Biolegend |  | 304834 | MM |  | N49-653 | BV650 | BD | 563746 |  |
|  | DREG-56 | BV650 | Biolegend |  | 304832 |  | cyIL4 | MP4-25D2 | APC | BD | 554486 | Th |
| CD69 | FN50 | BV421 | Biolegend |  | 310929 | AM | cyIL5 | TRFK5 | APC | BD | 554396 | Th |
| CD84 | 2G7 | BV421 | BD |  | 566094 | TFH | cyRORγt^δ^ | AFKJS-9 | APC | eBioscience | 17-6988-80 | Th |
| CD95 | DX2 | APC | BD |  | 558814 | MM | cyTbet^δ Ψ^ | 4B10 | PE | eBioscience | 12-5825 | Th |
| CD127 | HIL-7R-M21 | BV421 | BD |  | 562436 | Tregs |  | 4B10 | PE | Biolegend | 644810 |  |
|  | HIL-7R-M21 | BV510 | BD |  | 563086 |  |  | 4B10 | BV421 | Biolegend | 644815 |  |
|  | HIL-7R-M21 | BV711 | BD |  | 563165 |  | cyTCL1 | eBio1-21 | APC | eBioscience | 17-6699-42 | MM |
| cyCD154 | 24-31 | BV605 | Biolegend |  | 310826 | AM | cyTCRγδ | 11F2 | PE-Cy7 | BD | 655410 | GIT |
| CD161 | HP3G10 | FITC | Biolegend |  | 339905 | Th |  |  |  |  |  |  |
|  | DX12 | APC | BD |  | 550968 |  |  |  |  |  |  |  |
|  | HP3G10 | BV510 | Biolegend |  | 339921 |  |  |  |  |  |  |  |

Abbreviations (alphabetical order): AM: activation marker; AF: Alexa Fluor; APC: Allophycocyanin; APCCy7: Allophycocyanin-Cyanin 7; APCH7: Allophycocyanin-Hilite 7; BD: Becton Dickinson Biosciences; BV: Brilliant Violet; cy: intracellular; FITC: Fluorescein isothiocyanate; GIT: general identification of T cells; LI: leukocyte identification; TFH: follicular helper T-cell marker; Treg: regulatory T-cell marker; Th: identification of T helper subsets; MM: maturation marker; PerCPCy5.5: Peridinin-chlorophyll protein-complex cyanin 5.5; PE: Phycoerythrin; PE-Cy7: Phycoerythrin-Cyanin 7. EuroFlow standard operating procedures ([www.euroflow.org](http://www.euroflow.org)) were used for staining of the surface membrane (Sm) markers only or for simultaneous staining of Sm and intracellular markers using the Fix&Perm reagent (Thermo Fisher Scientific, Waltham, MA). ^δ^FoxP3, GATA3, RORγt and Tbet were stained using the eBioscience intracellular fixation and permeabilization buffer. ^Ψ^Biolegend Intracellular staining/permeabilization wash buffer was also tested for Tbet staining.

**Supplementary Table 2 -** Panel of monoclonal antibodies used for the detection of intracellular cytokines in

*in vitro* stimulation assays.

| **Tube** |  |  |  |  | **Fluorochrome-conjugated markers** | | | | | | |  |  |  |
| --- | --- | --- | --- | --- | --- | --- | --- | --- | --- | --- | --- | --- | --- | --- |
| ***1*** | **BV421** | **BV510** | **BV605** | **BV650** | **BV711** | **BV786** | **FITC** | **PerCPcy5.5** | **PE** | **PE-CF594** | **PE-Cy7** | **APC** | **AF700** | **APCH7** |
|  | CD69 | CD45RA | cyCD154 | CD27 | CD127 | CD3 | CD25 | HLADR | CD183 | CD196 | CD194 | cyIL4+ cyIL5 | cyIFNγ | CD4 |
| ***2*** | **BV421** | **BV510** | **BV605** | **BV650** | **BV711** | **BV786** | **FITC** | **PerCPcy5.5** | **PE** | **PE-CF594** | **PE-Cy7** | **AF647** | **AF700** | **APCH7** |
|  | CD69 | CD45RA | cyCD154 | CD27 | CD127 | CD3 | CD25 | HLADR | CD183 | CD196 | CD194 | cyIL17A | cyIFNγ | CD4 |

Abbreviations (alphabetical order): AF: Alexa Fluor; APC: Allophycocyanin; APCCy7: Allophycocyanin-Cyanin 7; APCH7: Allophycocyanin-Hilite 7; BV: Brilliant Violet; cy: intracellular; FITC: Fluorescein isothiocyanate; PerCPCy5.5: Peridinin-chlorophyll protein-complex cyanin 5.5; PE: Phycoerythrin; PE-Cy7: Phycoerythrin-Cyanin 7.

**Supplementary Table 3** – Immunophenotypic profiles used for the identification of the 22 FACS-sorted populations of blood T-cells investigated for their gene expression profiling (GEP) by qPCR.

| **FACS-sorted T-cell populations** | CD3 | CD4 | CD25 | CD127 | CD183 | CD185 | CD194 | CD196 | CCR10 |
| --- | --- | --- | --- | --- | --- | --- | --- | --- | --- |
| CD4+ naïve T cells | + | + | - | + | - | - | - | - | - |
| CD4+ Th1 cells | + | + | -/+ | -/+ | **+** | **-** | **-** | **-** | **-** |
| CD4+ Th2 cells | + | + | lo/+ | -/+ | - | - | + | - | - |
| CD4+ Th17 cells | + | + | lo/+ | -/+ | - | - | + | + | - |
| CD4+ Th1/Th17 cells | + | + | lo/+ | -/+ | + | - | - | + | - |
| CD4+ Th22 cells | + | + | lo/+ | -/+ | - | - | + | + | + |
| CD4+ CD183+ CD194+ CD196+ CCR10+ | + | + | lo/+ | -/+ | + | - | + | + | + |
| CD4+ CD183+ CD194+ CD196+ CCR10- | + | + | lo/+ | -/+ | + | - | + | + | - |
| CD4+ CD183+ CD194+ CD196- CCR10+ | + | + | lo/+ | -/+ | + | - | + | - | + |
| CD4+ CD183+ CD194+ CD196- CCR10- | + | + | lo/+ | -/+ | + | - | + | - | - |
| CD4+ CD183- CD194+ CD196- CCR10+ | + | + | lo/+ | -/+ | - | - | + | - | + |
| CD4+ Tregs | + | + | +hi | +lo | -/+ | -/+ | -/+ | -/+ | -/+ |
| Treg TFH | + | + | -/lo | +lo | -/+ | + | -/+ | -/+ | - |
| Th1-like TFH | + | + | -/lo | + | + | + | - | - | - |
| Th2-like TFH | + | + | -/lo | + | - | + | + | - | - |
| Th17-like TFH | + | + | -/lo | + | - | + | + | + | - |
| Th1/Th17-like TFH | + | + | -/lo | + | + | + | - | + | - |
| CD183- CD194- CD196- CCR10- TFH | + | + | -/lo | + | - | + | - | - | - |
| CD183+ CD194+ CD196+ CCR10- TFH | + | + | -/lo | + | + | + | + | + | - |
| CD183- CD194- CD196+ CCR10- TFH | + | + | -/lo | + | - | + | + | - | + |
| CD183+ CD194+ CD196- CCR10- TFH | + | + | -/lo | + | + | + | + | - | + |
| CD185+ CD4- T cells | + | - | -/lo | + | -/+ | + | -/+ | - | - |

*Phenotypic profile*

Abbreviations (alphabetical order): lo: low expression; TFH: follicular helper T cell; Th: T helper; Tregs: regulatory T cells;.

**Supplementary Table 4 -** Panel of genes (n=85) evaluated for gene expression profiling (GEP) analyses of PB T-cells classified according to their major functional role.

| **Associated T-cell profile** | **Genes** | **Associated T-cell profile** | **Genes** |
| --- | --- | --- | --- |
| **Th1** | *CXCR3* | **TFH** | *ASCL2* |
|  | *EOMES* |  | *BCL6* |
|  | *HLX* |  | *CD40LG* |
|  | *IFNG* |  | *CD84* |
|  | *IL2* |  | *CXCL13* |
|  | *IL12RB2* |  | *CXCR5* |
|  | *RARA* |  | *ICOS* |
|  | *RARB* |  | *IKZF1* |
|  | *RUNX3* |  | *IL21* |
|  | *TBX21* |  | *IL21R* |
|  | *TNF* |  | *KLF2* |
| **Th2** | *CCR4* |  | *MAF* |
|  | *CCR8* |  | *MYB* |
|  | *GATA3* |  | *NFATC1* |
|  | *IL4* |  | *NFATC2* |
|  | *IL4R* |  | *PDCD1* |
|  | *IL5* |  | *RC3H1* |
|  | *IL10* |  | *RC3H2* |
|  | *IL13* |  | *RUNX1* |
| **Th17** | *CCR6* |  | *TNFRSF4* |
|  | *HIF1A* |  | *S1PR2* |
|  | *IL6* |  | *VAV1* |
|  | *IL17A* | **Tregs** | *CTLA4* |
|  | *IL17F* |  | *ENTPD1* |
|  | *IL26* |  | *FOXP3* |
|  | *RORC* |  | *FOXO1* |
| **Th9** | *BATF* |  | *FOXO3* |
|  | *CCR3* |  | *NR4A1* |
|  | *IL9* |  | *PRDM1* |
|  | *IRF4* |  | *TGFB1* |
|  | *SPI1* | **Other** | *BACH2* |
| **Th22** | *AHR* |  | *EBI3* |
|  | *CCL15* |  | *FOXP1* |
|  | *CCL17* |  | *FOXP2* |
|  | *CCR10* |  | *FUT4* |
|  | *IL22* |  | *IL12A* |
| **Th25** | *IL25* |  | *TCF7* |
|  | *TRAF3IP2* |  | *STAT1* |
| **Control genes** | *CD4* |  | *STAT3* |
|  | *CD8A* |  | *STAT4* |
|  | *GAPDHᶲ* |  | *STAT5A* |
|  | *KITᵟ* |  | *STAT5B* |
|  |  |  | *STAT6* |

Abbreviations (alphabetical order): TFH: follicular helper T cell; Th: T helper; Tregs: regulatory T cells; ^φ^Positive Control; ^δ^Negative Control.

**Supplementary Table 5 –** Staining profiles for the different clones of monoclonal antibodies direct against CD185 tested in three peripheral blood samples from healthy adults (A, B and C) and their corresponding stain index (SI).

|  |  | **Clon 51505** | **Clon REA103** | **Clon J252D4** | **Clon MU5UBEE** |
| --- | --- | --- | --- | --- | --- |
|  |  | *(source: RnD Systems)* | *(source:*  *Miltenyi)* | *(source: Immunostep)* | *(source: eBioscience)* |
| **Sample** | **Anticoagulant** | **SI** | **SI** | **SI** | **SI** |
| A | EDTA | 28.25 | 59.58 | 31.13 | 16.51 |
| B | EDTA | 35.59 | 74.61 | 35.59 | 20.46 |
| C | EDTA | 41.5 | 70.44 | 41.07 | 25.38 |
| **Mean SI** |  | **35.11** | **68.21** | **35.93** | **20.78** |
| **SD** |  | 6.64 | 7.76 | 4.98 | 4.44 |

Abbreviations (alphabetical order): SD: standard deviation; SI: stain index.

SI was calculated as: [median fluorescence intensity (MFI) of positive population – MFI of negative population]/ [2*SD negative population].

**Supplementary Table 6–** Phenotypic profile of distinct populations (n=89) of CD4+ T cells recurrently identified in blood with the EuroFlow-IMM TCD4 tube.

*Phenotypic profile*

| **Populations of blood CD3+ CD4+ CD45^hi^ T-cells** | CD25 | CD27 | CD45RA | CD62L | CD127 | CD154* | CD183 | CD185 | CD194 | CD196 | CCR10 |
| --- | --- | --- | --- | --- | --- | --- | --- | --- | --- | --- | --- |
| CD4+ naïve T cells | - | + | + | + | + | - | - | - | - | - | - |
| CD4+ Th1 cells | -/+ | -/+ | -/+ | -/+ | -/+ | - | **+** | **-** | **-** | **-** | **-** |
| Central memory Th1 | lo/+ | + | - | + | + | - | **+** | **-** | **-** | **-** | **-** |
| Transitional memory Th1 | lo/+ | + | - | - | + | - | **+** | **-** | **-** | **-** | **-** |
| Effector memory Th1 | - | - | - | -/+ | -/+ | - | **+** | **-** | **-** | **-** | **-** |
| Terminal effector Th1 | - | - | + | -/+ | -/+ | - | **+** | **-** | **-** | **-** | **-** |
| CD4+ Th2 cells | lo/+ | -/+ | -/+ | -/+ | -/+ | - | - | - | + | - | - |
| Central memory Th2 | lo/+ | + | - | + | + | - | - | - | + | - | - |
| Transitional memory Th2 | lo/+ | + | - | - | + | - | - | - | + | - | - |
| Effector memory Th2 | lo/+ | - | - | -/+ | -/+ | - | - | - | + | - | - |
| Terminal effector Th2 | lo/+ | - | + | -/+ | -/+ | - | - | - | + | - | - |
| CD4+ Th17 cells | lo/+ | -/+ | -/+ | -/+ | -/+ | - | - | - | + | + | - |
| Central memory Th17 | lo/+ | + | - | + | + | - | - | - | + | + | - |
| Transitional memory Th17 | lo/+ | + | - | - | + | - | - | - | + | + | - |
| Effector memory Th17 | lo/+ | - | - | -/+ | -/+ | - | - | - | + | + | - |
| Terminal effector Th17 | lo/+ | - | + | -/+ | -/+ | - | - | - | + | + | - |
| CD4+ Th1/Th17 cells | lo/+ | -/+ | -/+ | -/+ | -/+ | - | + | - | - | + | - |
| Central memory Th1/Th17 | lo/+ | + | - | + | + | - | + | - | - | + | - |
| Transitional memory Th1/Th17 | lo/+ | + | - | - | + | - | + | - | - | + | - |
| Effector memory Th1/Th17 | lo/+ | - | - | -/+ | -/+ | - | + | - | - | + | - |
| Terminal effector Th1/Th17 | lo/+ | - | + | -/+ | -/+ | - | + | - | - | + | - |
| CD4+ Th22 cells | lo/+ | -/+ | -/+ | -/+ | -/+ | - | - | - | + | + | + |
| Central memory Th22 | lo/+ | + | - | + | + | - | - | - | + | + | + |
| Transitional memory Th22 | lo/+ | + | - | - | + | - | - | - | + | + | + |
| Effector memory Th22 | lo/+ | - | - | -/+ | -/+ | - | - | - | + | + | + |
| Terminal effector Th22 | lo/+ | - | + | -/+ | -/+ | - | - | - | + | + | + |
| CD4+ CD183+ CD194+ CD196+ CCR10+ | lo/+ | -/+ | -/+ | -/+ | -/+ | - | + | - | + | + | + |
| CM CD4+ CD183+ CD194+ CD196+ CCR10+ | lo/+ | + | - | + | + | - | + | - | + | + | + |
| TM CD4+ CD183+ CD194+ CD196+ CCR10+ | lo/+ | + | - | - | + | - | + | - | + | + | + |
| EM CD4+ CD183+ CD194+ CD196+ CCR10+ | lo/+ | - | - | -/+ | -/+ | - | + | - | + | + | + |
| TE CD4+ CD183+ CD194+ CD196+ CCR10+ | lo/+ | - | + | -/+ | -/+ | - | + | - | + | + | + |
| CD4+ CD183+ CD194+ CD196+ CCR10- | lo/+ | -/+ | -/+ | -/+ | -/+ | - | + | - | + | + | - |
| CM CD4+ CD183+ CD194+ CD196+ CCR10- | lo/+ | + | - | + | + | - | + | - | + | + | - |
| TM CD4+ CD183+ CD194+ CD196+ CCR10- | lo/+ | + | - | - | + | - | + | - | + | + | - |
| EM CD4+ CD183+ CD194+ CD196+ CCR10- | lo/+ | - | - | -/+ | -/+ | - | + | - | + | + | - |
| TE CD4+ CD183+ CD194+ CD196+ CCR10- | lo/+ | - | + | -/+ | -/+ | - | + | - | + | + | - |

**Supplementary Table 6** *(Continued)*

*Phenotypic profile*

| **Populations of blood CD3+ CD4+ CD45^hi^ T cells** | CD25 | CD27 | CD45RA | CD62L | CD127 | CD154* | CD183 | CD185 | CD194 | CD196 | CCR10 |
| --- | --- | --- | --- | --- | --- | --- | --- | --- | --- | --- | --- |
| CD4+ CD183+ CD194+ CD196- CCR10+ | lo/+ | -/+ | -/+ | -/+ | -/+ | - | + | - | + | - | + |
| CM CD4+ CD183+ CD194+ CD196- CCR10+ | lo/+ | + | - | + | + | - | + | - | + | - | + |
| TM CD4+ CD183+ CD194+ CD196- CCR10+ | lo/+ | + | - | - | + | - | + | - | + | - | + |
| EM CD4+ CD183+ CD194+ CD196- CCR10+ | lo/+ | - | - | -/+ | -/+ | - | + | - | + | - | + |
| TE CD4+ CD183+ CD194+ CD196- CCR10+ | lo/+ | - | + | -/+ | -/+ | - | + | - | + | - | + |
| CD4+ CD183+ CD194+ CD196- CCR10- | lo/+ | -/+ | -/+ | -/+ | -/+ | - | + | - | + | - | - |
| CM CD4+ CD183+ CD194+ CD196- CCR10- | lo/+ | + | - | + | + | - | + | - | + | - | - |
| TM CD4+ CD183+ CD194+ CD196- CCR10- | lo/+ | + | - | - | + | - | + | - | + | - | - |
| EM CD4+ CD183+ CD194+ CD196- CCR10- | lo/+ | - | - | -/+ | -/+ | - | + | - | + | - | - |
| TE CD4+ CD183+ CD194+ CD196- CCR10- | lo/+ | - | + | -/+ | -/+ | - | + | - | + | - | - |
| CD4+ CD183+ CD194- CD196+ CCR10+ | lo/+ | -/+ | -/+ | -/+ | -/+ | - | + | - | - | + | + |
| CM CD4+ CD183+ CD194- CD196+ CCR10+ | lo/+ | + | - | + | + | - | + | - | - | + | + |
| TM CD4+ CD183+ CD194- CD196+ CCR10+ | lo/+ | + | - | - | + | - | + | - | - | + | + |
| EM CD4+ CD183+ CD194- CD196+ CCR10+ | lo/+ | - | - | -/+ | -/+ | - | + | - | - | + | + |
| TE CD4+ CD183+ CD194- CD196+ CCR10+ | lo/+ | - | + | -/+ | -/+ | - | + | - | - | + | + |
| CD4+ CD183+ CD194- CD196- CCR10+ | lo/+ | -/+ | -/+ | -/+ | -/+ | - | + | - | - | - | + |
| CM CD4+ CD183+ CD194- CD196- CCR10+ | lo/+ | + | - | + | + | - | + | - | - | - | + |
| TM CD4+ CD183+ CD194- CD196- CCR10+ | lo/+ | + | - | - | + | - | + | - | - | - | + |
| EM CD4+ CD183+ CD194- CD196- CCR10+ | lo/+ | - | - | -/+ | -/+ | - | + | - | - | - | + |
| TE CD4+ CD183+ CD194- CD196- CCR10+ | lo/+ | - | + | -/+ | -/+ | - | + | - | - | - | + |
| CD4+ CD183- CD194- CD196+ CCR10- | lo/+ | -/+ | -/+ | -/+ | -/+ | - | - | - | - | + | - |
| CM CD4+ CD183- CD194- CD196+ CCR10- | lo/+ | + | - | + | + | - | - | - | - | + | - |
| TM CD4+ CD183- CD194- CD196+ CCR10- | lo/+ | + | - | - | + | - | - | - | - | + | - |
| EM CD4+ CD183- CD194- CD196+ CCR10- | lo/+ | - | - | -/+ | -/+ | - | - | - | - | + | - |
| TE CD4+ CD183- CD194- CD196+ CCR10- | lo/+ | - | + | -/+ | -/+ | - | - | - | - | + | - |
| CD4+ CD183- CD194+ CD196- CCR10+ | lo/+ | -/+ | -/+ | -/+ | -/+ | - | - | - | + | - | + |
| CM CD4+ CD183- CD194+ CD196- CCR10+ | lo/+ | + | - | + | + | - | - | - | + | - | + |
| TM CD4+ CD183- CD194+ CD196- CCR10+ | lo/+ | + | - | - | + | - | - | - | + | - | + |
| EM CD4+ CD183- CD194+ CD196- CCR10+ | lo/+ | - | - | -/+ | -/+ | - | - | - | + | - | + |
| TE CD4+ CD183- CD194+ CD196- CCR10+ | lo/+ | - | + | -/+ | -/+ | - | - | - | + | - | + |
| Non-naïve CD4+ CD183- CD194- CD196- CCR10- | lo/+ | -/+ | - | -/+ |  | - | - | - | - | - | - |

**Supplementary Table 6** *(Continued)*

*Phenotypic profile*

| **Populations of blood CD3+ CD4+ CD45^hi^ T cells** | CD25 | CD27 | CD45RA | CD62L | CD127 | CD154* | CD183 | CD185 | CD194 | CD196 | CCR10 |
| --- | --- | --- | --- | --- | --- | --- | --- | --- | --- | --- | --- |
| CD4+ Tregs | +hi | -/+ | -/+ | -/+ | +lo | - | -/+ | -/+ | -/+ | -/+ | -/+ |
| Naïve Treg | +hi | + | + | + | +lo | - | - | - | - | - | - |
| Th1-like Treg | +hi | -/+ | -/+ | -/+ | +lo | - | + | - | - | - | - |
| Th2-like Treg | +hi | -/+ | -/+ | -/+ | +lo | - | - | - | + | - | - |
| Th17-like Treg | +hi | -/+ | -/+ | -/+ | +lo | - | - | - | + | + | - |
| Th22-like Treg | +hi | -/+ | -/+ | -/+ | +lo | - | - | - | + | + | + |
| CD183+ CD194+ CD196- CCR10+ Treg | +hi | -/+ | -/+ | -/+ | +lo | - | + | - | + | - | + |
| CD183+ CD194+ CD196- CCR10- Treg | +hi | -/+ | -/+ | -/+ | +lo | - | + | - | + | - | - |
| CD183+ CD194+ CD196+ CCR10- Treg | +hi | -/+ | -/+ | -/+ | +lo | - | + | - | + | + | - |
| CD183+ CD194+ CD196+ CCR10+ Treg | +hi | -/+ | -/+ | -/+ | +lo | - | + | - | + | + | + |
| CD183- CD194+ CD196- CCR10+ Treg | +hi | -/+ | -/+ | -/+ | +lo | - | - | - | + | - | + |
| CD4+ TFH cells | -/lo | -/+ | -/+ | -/+ | -/+ | - | -/+ | + | -/+ | -/+ | - |
| Treg TFH | -/lo | -/+ | -/+ | -/+ | +lo | - | -/+ | + | -/+ | -/+ | - |
| Th1-like TFH | -/lo | -/+ | -/+ | -/+ | + | - | + | + | - | - | - |
| Th2-like TFH | -/lo | -/+ | -/+ | -/+ | + | - | - | + | + | - | - |
| Th17-like TFH | -/lo | -/+ | -/+ | -/+ | + | - | - | + | + | + | - |
| Th1/Th17-like TFH | -/lo | -/+ | -/+ | -/+ | + | - | + | + | - | + | - |
| CD183+ CD194+ CD196- CCR10- TFH | -/lo | -/+ | -/+ | -/+ | + | - | + | + | + | - | - |
| CD183+ CD194+ CD196+ CCR10- TFH | -/lo | -/+ | -/+ | -/+ | + | - | + | + | + | + | - |
| CD183- CD194- CD196+ CCR10- TFH | -/lo | -/+ | -/+ | -/+ | + | - | - | + | - | + | - |
| CD183- CD194- CD196- CCR10- TFH | -/lo | -/+ | -/+ | -/+ | + | - | - | + | - | - | - |
| CD4+ CD185+ CD27+ CD45RA+ CD62L+ T cells | -/lo | + | + | + | + | - | - | + | - | - | - |

**CD154 was only expressed by (in vitro) activated T cells while systematically negative in fresh blood CD4+ T-cells.* Abbreviations (alphabetical order): CM: central memory; EM: effector memory; het: heterogeneous expression; hi: high expression; lo: low expression; TE: terminal effector; TM: transitional memory.

**Supplementary Table 7 –** Relative expression levels for all 85 genes studied in all different FACS-sorted blood T-cell subsets (n=22) obtained from 6 healthy donors.

*T-cell subsets*

| **GENES** | **CD4+ naïve T cells** | **CD4+ Th1 cells** | **CD4+ Th2 cells** | **CD4+ Th17 cells** | **CD4+ Th1/Th17 cells** | **CD4+ Th22 cells** | **CD4+ CD183+ CD194+ CD196+ CCR10+** | **CD4+ CD183+ CD194+ CD196+ CCR10-** | **CD4+ CD183+ CD194+ CD196- CCR10+** | **CD4+ CD183+ CD194+ CD196- CCR10-** | **CD4+ CD183- CD194+ CD196- CCR10+** | **CD4+ Tregs** | **Treg TFH** | **Th1-like TFH** | **Th2-like TFH** | **Th17-like TFH** | **Th1/Th17-like TFH** | **CD183- CD194- CD196- CCR10- TFH** | **CD183+ CD194+ CD196+ CCR10- TFH** | **CD183- CD194- CD196+ CCR10- TFH** | **CD183+ CD194+ CD196- CCR10- TFH** | **CD185+ CD4- T cells** |
| --- | --- | --- | --- | --- | --- | --- | --- | --- | --- | --- | --- | --- | --- | --- | --- | --- | --- | --- | --- | --- | --- | --- |
| AHR | -4.44 | -3.74 | -3.01 | -2.92 | -3.15 | -1.85 | -2.12 | -3.27 | -3.47 | -3.09 | -2.93 | -3.73 | -3.94 | -3.26 | -3.34 | -3.07 | -3.09 | -3.71 | -2.97 | -3.11 | -3.90 | -5.04 |
| ASCL2 | -7.24 | -10.23 | -7.48 | -5.00 | -5.13 | -7.27 | -8.62 | -6.76 | -8.13 | -7.61 | -6.82 | -7.78 | -9.51 | NA | -5.40 | -5.55 | NA | NA | NA | NA | NA | -6.33 |
| BACH2 | -2.90 | -4.95 | -5.04 | -5.83 | -4.91 | -6.11 | NA | -5.14 | -6.07 | -5.71 | -6.01 | -6.62 | -5.75 | -4.59 | -5.58 | -6.12 | -4.72 | -4.91 | -5.03 | -4.84 | -5.68 | -3.05 |
| BATF | -5.04 | -4.33 | -4.60 | -5.41 | -4.73 | -3.48 | -3.53 | -5.96 | -5.05 | -4.52 | -3.75 | -3.95 | -4.41 | -3.62 | -4.57 | -4.57 | -3.96 | -4.84 | -4.14 | -4.35 | -4.27 | -5.87 |
| BCL6 | -6.24 | -5.92 | -7.38 | -6.96 | -6.11 | -6.74 | -6.49 | NA | -6.35 | -7.27 | -7.72 | -7.22 | -7.39 | -6.83 | -6.83 | -7.36 | -6.32 | -6.14 | -7.03 | -6.87 | -6.18 | -5.53 |
| CCL15 | NA | NA | NA | NA | NA | NA | NA | NA | NA | NA | NA | NA | NA | NA | NA | NA | NA | NA | NA | NA | NA | NA |
| CCL17 | NA | NA | NA | NA | NA | NA | NA | NA | NA | NA | NA | NA | NA | NA | NA | NA | NA | NA | NA | NA | NA | NA |
| CCR10 | NA | NA | NA | NA | NA | -7.63 | NA | NA | -7.43 | NA | -6.57 | -7.23 | NA | NA | NA | NA | NA | NA | NA | NA | NA | NA |
| CCR3 | -3.25 | -3.54 | -3.49 | -3.63 | -2.01 | -2.54 | -3.41 | -2.78 | -2.79 | -3.35 | -3.61 | -3.89 | -4.50 | -4.02 | -3.55 | -5.07 | -4.78 | -4.05 | -3.85 | -4.19 | -4.33 | -2.68 |
| CCR4 | NA | NA | 2.28 | 2.72 | NA | 2.29 | NA | NA | 3.23 | NA | 2.52 | 1.65 | 0.79 | NA | 3.41 | 2.13 | NA | NA | 2.87 | NA | NA | NA |
| CCR6 | -3.69 | -3.63 | -4.31 | -2.51 | -2.96 | -2.45 | -2.88 | -2.28 | -3.53 | -3.51 | -3.74 | -3.87 | -3.75 | -3.65 | -3.99 | -2.92 | -2.93 | -4.17 | -2.65 | -2.76 | -4.26 | -2.32 |
| CCR8 | NA | NA | -5.94 | NA | NA | -4.19 | -6.25 | NA | -5.77 | -6.96 | -4.69 | -4.72 | -9.15 | NA | NA | NA | NA | NA | NA | NA | NA | NA |
| CD4 | -2.69 | -3.23 | -2.82 | -3.69 | -3.71 | -2.98 | -3.72 | -4.17 | -4.68 | -3.01 | -3.37 | -3.37 | -3.33 | -2.64 | -2.62 | -2.38 | -2.50 | -2.60 | -3.14 | -2.86 | -2.56 | NA |
| CD40LG | -2.01 | -2.18 | -1.53 | -2.47 | -1.78 | -1.61 | -1.64 | -1.73 | -2.61 | -2.03 | -1.86 | -4.58 | -4.03 | -1.51 | -1.38 | -1.69 | -1.48 | -1.50 | -0.83 | -1.22 | -1.39 | -7.39 |
| CD84 | -5.00 | -4.46 | -3.85 | -4.33 | -4.52 | -3.67 | -4.54 | -5.73 | -4.45 | -4.21 | -3.91 | -4.83 | -5.15 | -3.16 | -3.55 | -3.74 | -3.53 | -3.58 | -4.03 | -3.46 | -2.96 | -4.33 |
| CD8A | NA | NA | NA | NA | NA | NA | NA | NA | NA | NA | NA | NA | NA | NA | NA | NA | NA | NA | NA | NA | NA | NA |
| CTLA4 | -2.76 | -2.55 | -2.82 | -2.21 | -3.02 | -2.23 | -2.75 | -2.14 | -2.61 | -2.11 | -3.20 | -1.37 | -2.00 | -2.64 | -2.53 | -2.51 | -3.19 | -2.51 | -2.69 | -2.70 | -2.73 | -2.75 |
| CXCL13 | -5.59 | -5.13 | -5.85 | -5.10 | -4.92 | -5.11 | -6.80 | -5.11 | -5.23 | -5.16 | -5.10 | -6.58 | -4.63 | -5.58 | -4.84 | -4.53 | -4.42 | -2.20 | -4.94 | -3.63 | -4.89 | -4.90 |
| CXCR3 | -3.46 | -2.98 | -3.63 | -4.05 | -2.97 | -3.87 | -3.54 | -2.84 | -3.14 | -2.81 | -4.59 | -4.27 | -4.14 | -3.45 | -4.33 | -5.41 | -3.46 | -4.61 | -3.14 | -4.00 | -3.11 | -3.00 |

**Supplementary Table 7** *(Continued)*

*T-cell subsets*

| **GENES** | **CD4+ naïve T cells** | **CD4+ Th1 cells** | **CD4+ Th2 cells** | **CD4+ Th17 cells** | **CD4+ Th1/Th17 cells** | **CD4+ Th22 cells** | **CD4+ CD183+ CD194+ CD196+ CCR10+** | **CD4+ CD183+ CD194+ CD196+ CCR10-** | **CD4+ CD183+ CD194+ CD196- CCR10+** | **CD4+ CD183+ CD194+ CD196- CCR10-** | **CD4+ CD183- CD194+ CD196- CCR10+** | **CD4+ Tregs** | **Treg TFH** | **Th1-like TFH** | **Th2-like TFH** | **Th17-like TFH** | **Th1/Th17-like TFH** | **CD183- CD194- CD196- CCR10- TFH** | **CD183+ CD194+ CD196+ CCR10- TFH** | **CD183- CD194- CD196+ CCR10- TFH** | **CD183+ CD194+ CD196- CCR10- TFH** | **CD185+ CD4- T cells** |
| --- | --- | --- | --- | --- | --- | --- | --- | --- | --- | --- | --- | --- | --- | --- | --- | --- | --- | --- | --- | --- | --- | --- |
| CXCR5 | -2.88 | -3.98 | -3.93 | -3.32 | -4.11 | -2.76 | -3.59 | -2.90 | -2.64 | -2.94 | -4.17 | -5.22 | -3.59 | -3.07 | -2.74 | -3.08 | -3.28 | -2.95 | -2.96 | -2.57 | -3.13 | -2.22 |
| EBI3 | NA | NA | NA | NA | NA | NA | NA | NA | NA | NA | NA | NA | NA | NA | NA | NA | NA | NA | NA | NA | NA | -7.06 |
| ENTPD1 | -9.07 | -8.28 | -7.44 | NA | NA | -7.65 | -5.67 | NA | -8.38 | -7.46 | -7.90 | -4.51 | -7.04 | -8.43 | -7.46 | -7.59 | -7.48 | -7.95 | -6.88 | -5.59 | -7.97 | -3.32 |
| EOMES | -7.94 | -4.54 | -8.41 | -8.94 | -4.04 | -8.84 | -7.08 | -6.78 | -6.99 | -5.40 | -8.47 | -9.33 | -8.15 | -4.30 | -9.12 | -8.70 | -6.28 | -8.68 | -6.67 | -8.29 | -5.40 | -6.85 |
| FOXO1 | -0.15 | -1.37 | -1.07 | -1.74 | -1.55 | -1.25 | -0.80 | -1.51 | -1.48 | -1.18 | -1.28 | -1.05 | -1.57 | -0.64 | -0.50 | -1.14 | -0.51 | -0.54 | -0.62 | -0.67 | -0.82 | -0.87 |
| FOXO3 | -4.11 | -4.82 | -4.77 | -4.63 | -3.92 | -4.15 | -4.90 | -4.24 | -3.91 | -4.06 | -5.37 | -5.59 | -5.65 | -5.64 | -4.95 | -6.10 | -5.21 | -5.29 | -5.08 | -6.23 | -4.95 | -5.14 |
| FOXP1 | -0.31 | -1.37 | -0.71 | -1.96 | -1.52 | -1.60 | -1.26 | -1.87 | -1.92 | -0.96 | -1.35 | -1.46 | -1.50 | -0.82 | -0.62 | -1.09 | -0.53 | -0.44 | -0.77 | -0.49 | -0.57 | -0.71 |
| FOXP2 | NA | NA | NA | NA | NA | NA | NA | NA | NA | NA | NA | NA | NA | NA | NA | NA | NA | NA | NA | NA | NA | NA |
| FOXP3 | -9.54 | -7.28 | -7.83 | NA | NA | -8.29 | NA | NA | -7.11 | -6.86 | -7.86 | -1.86 | -3.45 | -5.43 | -6.70 | -6.35 | -6.84 | -7.01 | -5.98 | -6.22 | -6.82 | -6.94 |
| FUT4 | -2.96 | -3.14 | -3.00 | -3.23 | -3.18 | -2.80 | -3.68 | -2.32 | -2.59 | -2.64 | -3.27 | -4.53 | -4.86 | -3.06 | -3.06 | -4.31 | -4.16 | -3.82 | -3.06 | -3.21 | -4.26 | -3.09 |
| GAPDH | 0.00 | 0.00 | 0.00 | 0.00 | 0.00 | 0.00 | 0.00 | 0.00 | 0.00 | 0.00 | 0.00 | 0.00 | 0.00 | 0.00 | 0.00 | 0.00 | 0.00 | 0.00 | 0.00 | 0.00 | 0.00 | 0.00 |
| GATA-3 | -8.38 | -6.39 | -6.45 | -6.00 | -7.33 | -6.17 | -5.97 | -7.36 | NA | NA | -5.80 | -5.74 | -6.63 | -6.73 | -5.36 | -5.87 | NA | -6.97 | -5.32 | -6.75 | -5.68 | -6.09 |
| HIF1A | -2.59 | -3.33 | -3.19 | -3.31 | -3.72 | -3.09 | -2.76 | -3.32 | -3.70 | -3.04 | -3.41 | -4.16 | -3.66 | -2.73 | -2.83 | -2.96 | -2.41 | -2.80 | -2.57 | -2.58 | -2.73 | -2.63 |
| HLX | NA | -10.49 | NA | NA | NA | NA | NA | NA | NA | NA | NA | -8.76 | -10.36 | NA | NA | NA | NA | NA | NA | NA | NA | -6.19 |
| ICOS | -3.21 | -3.58 | -2.71 | -3.55 | -3.83 | -3.60 | -3.50 | -3.88 | -3.54 | -3.06 | -3.12 | -2.92 | -3.51 | -2.58 | -2.62 | -2.93 | -2.51 | -2.65 | -2.50 | -2.00 | -2.61 | NA |
| IFNG | NA | -5.40 | NA | NA | -4.65 | NA | NA | -5.62 | -5.11 | -5.78 | -8.29 | -6.52 | -7.36 | -5.58 | NA | NA | -6.50 | NA | -4.75 | NA | -6.42 | NA |
| IKZF1 | -1.12 | -1.94 | -1.60 | -2.23 | -2.19 | -1.93 | -2.31 | -2.51 | -2.21 | -1.89 | -1.83 | -2.60 | -2.62 | -1.71 | -1.77 | -1.76 | -1.71 | -1.43 | -2.11 | -1.61 | -1.78 | -1.94 |
| IL10 | -4.64 | -6.03 | -6.55 | NA | NA | -6.72 | -5.45 | NA | -7.23 | -6.83 | -5.98 | -7.75 | -8.11 | -5.21 | -5.70 | -5.40 | -5.20 | -5.17 | -6.00 | -5.16 | -6.20 | -6.50 |
| IL12A | -7.11 | -6.36 | -8.01 | -6.67 | -6.55 | -5.65 | -7.22 | -5.78 | -5.69 | NA | -6.40 | -7.81 | -9.81 | -6.84 | -5.88 | -8.86 | -5.64 | -4.61 | -3.89 | -5.07 | -5.17 | -5.75 |
| IL12RB2 | -9.63 | -6.41 | -8.84 | -7.77 | -5.35 | -10.01 | NA | -6.33 | -9.18 | -6.61 | -9.13 | -5.81 | -7.01 | -7.85 | NA | -7.25 | -7.52 | NA | -6.22 | NA | -7.22 | NA |
| IL13 | NA | NA | NA | NA | NA | -8.85 | NA | NA | NA | NA | -7.77 | NA | NA | NA | NA | NA | NA | NA | NA | NA | NA | NA |

**Supplementary Table 7** *(Continued)*

*T-cell subsets*

| **GENES** | **CD4+ naïve T cells** | **CD4+ Th1 cells** | **CD4+ Th2 cells** | **CD4+ Th17 cells** | **CD4+ Th1/Th17 cells** | **CD4+ Th22 cells** | **CD4+ CD183+ CD194+ CD196+ CCR10+** | **CD4+ CD183+ CD194+ CD196+ CCR10-** | **CD4+ CD183+ CD194+ CD196- CCR10+** | **CD4+ CD183+ CD194+ CD196- CCR10-** | **CD4+ CD183- CD194+ CD196- CCR10+** | **CD4+ Tregs** | **Treg TFH** | **Th1-like TFH** | **Th2-like TFH** | **Th17-like TFH** | **Th1/Th17-like TFH** | **CD183- CD194- CD196- CCR10- TFH** | **CD183+ CD194+ CD196+ CCR10- TFH** | **CD183- CD194- CD196+ CCR10- TFH** | **CD183+ CD194+ CD196- CCR10- TFH** | **CD185+ CD4- T cells** |
| --- | --- | --- | --- | --- | --- | --- | --- | --- | --- | --- | --- | --- | --- | --- | --- | --- | --- | --- | --- | --- | --- | --- |
| IL17A | NA | NA | NA | NA | NA | NA | NA | NA | NA | NA | NA | ### | NA | NA | NA | NA | NA | NA | NA | NA | NA | NA |
| IL17F | NA | NA | NA | NA | NA | NA | NA | NA | NA | NA | NA | NA | NA | NA | NA | NA | NA | NA | NA | NA | NA | NA |
| IL2 | -7.38 | -7.20 | -5.07 | -4.31 | -3.86 | -4.57 | -3.90 | -4.55 | -4.32 | -5.90 | -4.08 | -7.45 | -7.29 | -6.05 | -5.47 | -7.16 | -6.24 | -6.35 | -4.19 | -5.25 | -5.35 | -5.92 |
| IL21 | NA | NA | NA | NA | NA | NA | NA | NA | NA | -8.84 | NA | -9.03 | -8.36 | NA | NA | -8.62 | NA | NA | NA | NA | -8.70 | -7.07 |
| IL21R | -4.63 | -5.92 | -5.34 | -6.15 | -6.95 | -5.79 | -5.63 | NA | -6.84 | -6.51 | -6.02 | -6.21 | -6.04 | -4.39 | -5.37 | -5.47 | -4.90 | -4.93 | -5.33 | -4.90 | -5.34 | -5.83 |
| IL22 | -3.15 | -9.12 | -4.49 | -3.84 | -5.69 | -4.76 | NA | NA | -9.89 | NA | -3.87 | -2.97 | NA | -2.89 | -4.92 | NA | NA | -3.47 | NA | -1.73 | NA | -2.25 |
| IL25 | NA | NA | NA | NA | NA | NA | NA | NA | NA | NA | NA | NA | NA | NA | NA | NA | NA | NA | NA | NA | NA | NA |
| IL26 | NA | NA | NA | NA | NA | -8.58 | NA | NA | NA | NA | NA | -8.54 | -9.86 | NA | NA | NA | NA | NA | NA | NA | NA | NA |
| IL4 | NA | -11.69 | -8.94 | NA | NA | NA | NA | NA | -8.89 | -9.74 | NA | NA | NA | NA | -8.33 | NA | NA | NA | NA | NA | NA | NA |
| IL4R | -4.01 | -5.13 | -3.56 | -6.36 | -5.60 | -5.10 | -4.35 | -6.70 | -5.69 | -5.10 | -4.19 | -6.19 | -5.69 | -4.94 | -5.15 | -5.15 | -4.87 | -4.02 | -5.11 | -4.32 | -5.22 | -3.07 |
| IL5 | NA | NA | NA | NA | NA | NA | NA | NA | NA | NA | NA | NA | NA | NA | NA | NA | NA | NA | NA | NA | NA | NA |
| IL6 | NA | -8.67 | NA | NA | NA | NA | NA | NA | NA | NA | NA | NA | NA | NA | NA | NA | NA | NA | NA | NA | NA | -6.60 |
| IL9 | -3.79 | -3.53 | -3.68 | -3.99 | -2.42 | -3.62 | -3.20 | -2.78 | -3.26 | -3.84 | -3.05 | -4.72 | -4.21 | -3.73 | -2.38 | -3.16 | -3.01 | -3.74 | -2.18 | -3.09 | -3.40 | -3.15 |
| IRF4 | -5.86 | -6.63 | -7.27 | -6.48 | NA | -10.94 | NA | NA | -7.79 | -6.89 | -7.50 | -6.17 | -6.61 | -6.43 | -6.47 | -6.89 | -6.32 | -6.31 | -7.24 | -6.34 | -6.41 | -4.31 |
| KIT | NA | NA | NA | NA | NA | NA | NA | NA | NA | NA | NA | NA | NA | NA | NA | NA | NA | NA | NA | NA | NA | NA |
| KLF2 | 0.05 | -0.44 | -0.19 | -1.09 | -1.02 | -0.37 | -0.15 | -0.84 | -0.89 | -0.69 | -0.27 | -0.77 | -1.35 | 0.25 | 0.35 | 0.15 | 0.50 | 0.11 | 0.06 | -0.39 | 0.17 | -0.26 |
| MAF | -4.52 | -2.81 | -2.78 | -2.38 | -3.49 | -2.92 | -2.64 | -3.52 | -2.93 | -2.49 | -3.50 | -3.07 | -3.09 | -3.05 | -1.96 | -1.49 | -2.27 | -2.82 | -2.48 | -2.38 | -2.52 | -5.67 |
| MYB | -5.79 | -7.61 | -8.04 | NA | NA | -10.39 | NA | NA | -8.12 | NA | -8.40 | -7.12 | -6.71 | -6.63 | -8.08 | -8.47 | -8.31 | -7.30 | -7.25 | -7.62 | -7.43 | -9.04 |
| NFATC1 | -4.19 | -5.03 | -4.49 | -5.99 | -6.24 | -5.24 | -6.20 | -7.12 | -5.87 | -5.11 | -5.69 | -6.59 | -5.54 | -3.97 | -4.65 | -4.46 | -4.02 | -4.39 | -4.60 | -4.26 | -4.42 | -4.62 |
| NFATC2 | -2.17 | -2.21 | -1.88 | -2.56 | -3.49 | -2.38 | -2.25 | -2.93 | -2.97 | -2.28 | -2.33 | -3.14 | -3.02 | -2.15 | -2.16 | -2.42 | -1.87 | -2.44 | -2.04 | -1.91 | -1.94 | -2.26 |

**Supplementary Table 7** *(Continued)*

*T-cell subsets*

| **GENES** | **CD4+ naïve T cells** | **CD4+ Th1 cells** | **CD4+ Th2 cells** | **CD4+ Th17 cells** | **CD4+ Th1/Th17 cells** | **CD4+ Th22 cells** | **CD4+ CD183+ CD194+ CD196+ CCR10+** | **CD4+ CD183+ CD194+ CD196+ CCR10-** | **CD4+ CD183+ CD194+ CD196- CCR10+** | **CD4+ CD183+ CD194+ CD196- CCR10-** | **CD4+ CD183- CD194+ CD196- CCR10+** | **CD4+ Tregs** | **Treg TFH** | **Th1-like TFH** | **Th2-like TFH** | **Th17-like TFH** | **Th1/Th17-like TFH** | **CD183- CD194- CD196- CCR10- TFH** | **CD183+ CD194+ CD196+ CCR10- TFH** | **CD183- CD194- CD196+ CCR10- TFH** | **CD183+ CD194+ CD196- CCR10- TFH** | **CD185+ CD4- T cells** |
| --- | --- | --- | --- | --- | --- | --- | --- | --- | --- | --- | --- | --- | --- | --- | --- | --- | --- | --- | --- | --- | --- | --- |
| NR4A1 | -7.26 | -5.80 | -8.22 | -3.42 | NA | -6.40 | NA | NA | NA | NA | -4.52 | -7.68 | NA | -6.17 | NA | -6.84 | -6.06 | -7.56 | NA | NA | NA | -5.44 |
| PDCD1 | NA | NA | NA | NA | NA | NA | NA | NA | NA | NA | NA | NA | NA | NA | -3.49 | -4.20 | NA | NA | NA | NA | NA | NA |
| PRDM1 | -5.65 | -3.53 | -3.67 | -3.87 | -3.46 | -3.01 | -2.96 | -3.72 | -3.54 | -3.29 | -3.55 | -3.38 | -4.40 | -4.05 | -4.28 | -3.70 | -3.58 | -5.12 | -3.60 | -4.02 | -4.19 | -4.96 |
| RARA | -4.79 | -4.27 | -3.61 | -3.82 | -4.43 | -3.45 | -3.84 | -4.92 | -4.35 | -3.90 | -3.50 | -3.97 | -4.69 | -4.90 | -4.27 | -3.89 | -3.81 | -4.29 | -3.84 | -4.21 | -4.41 | -3.87 |
| RARB | NA | NA | NA | NA | NA | NA | NA | NA | NA | NA | NA | NA | NA | NA | NA | NA | NA | NA | NA | NA | NA | NA |
| RC3H1 | -3.11 | -4.11 | -3.15 | -4.39 | -5.15 | -3.99 | -4.48 | -4.86 | -4.57 | -4.05 | -4.14 | -4.44 | -4.79 | -3.89 | -3.80 | -3.77 | -3.40 | -3.75 | -4.24 | -3.80 | -3.50 | -3.62 |
| RC3H2 | -3.65 | -4.79 | -3.89 | -5.35 | -5.51 | -4.34 | -4.56 | -5.56 | -4.83 | -4.22 | -4.98 | -5.09 | -5.04 | -4.24 | -4.37 | -4.78 | -4.16 | -4.42 | -4.23 | -3.93 | -4.16 | -5.12 |
| RORC | -9.93 | -8.64 | -10.50 | -4.56 | -5.61 | -6.24 | -5.37 | NA | -10.37 | -9.62 | -9.68 | -7.35 | -7.40 | NA | -7.86 | -6.32 | -7.60 | NA | -6.49 | -6.40 | NA | NA |
| RUNX1 | -2.24 | -3.33 | -2.65 | -3.41 | -3.77 | -3.00 | -2.79 | -3.43 | -3.62 | -3.06 | -3.26 | -3.33 | -4.19 | -2.46 | -2.57 | -2.60 | -2.36 | -2.90 | -2.83 | -3.13 | -2.95 | -4.22 |
| RUNX3 | -3.16 | -2.93 | -3.29 | -4.04 | -3.67 | -3.98 | -3.13 | -4.56 | -4.06 | -3.36 | -3.47 | -3.95 | -4.65 | -3.11 | -3.79 | -4.31 | -3.17 | -4.20 | -4.29 | -3.45 | -3.88 | -3.98 |
| S1PR2 | NA | NA | NA | NA | NA | NA | NA | NA | NA | NA | NA | NA | NA | NA | NA | NA | NA | NA | NA | NA | NA | NA |
| SPI1 | -6.69 | -6.36 | -6.87 | -5.90 | -6.51 | -5.54 | -7.34 | -7.54 | -5.72 | -7.90 | -5.24 | -7.72 | -6.59 | -5.56 | -6.42 | -6.66 | -6.26 | -5.56 | -6.42 | -6.14 | -6.31 | -2.46 |
| STAT1 | -2.19 | -2.70 | -2.43 | -3.36 | -3.90 | -2.53 | -2.55 | -3.77 | -2.97 | -2.43 | -2.91 | -2.81 | -3.58 | -2.02 | -2.60 | -2.82 | -1.85 | -2.88 | -2.20 | -2.86 | -2.14 | -4.04 |
| STAT3 | -3.61 | -4.05 | -3.66 | -4.90 | -4.47 | -4.43 | -4.94 | -4.78 | -5.79 | -4.00 | -4.31 | -4.57 | -4.42 | -2.97 | -3.55 | -3.67 | -3.42 | -3.60 | -4.75 | -3.37 | -3.94 | -3.74 |
| STAT4 | -4.62 | -4.41 | -4.68 | -5.26 | -4.45 | -4.28 | -4.18 | -5.51 | -5.60 | -4.35 | -4.81 | -6.47 | -6.26 | -4.71 | -5.80 | -5.41 | -4.34 | -4.89 | -5.42 | -5.12 | -4.70 | -8.24 |
| STAT5A | -4.59 | -5.37 | -4.78 | -5.88 | -5.70 | -4.66 | -4.96 | -5.61 | -5.09 | -5.30 | -5.38 | -5.36 | -5.10 | -4.24 | -4.67 | -4.75 | -4.18 | -4.93 | -5.27 | -4.57 | -4.10 | -5.13 |
| STAT5B | -3.05 | -3.38 | -3.04 | -3.55 | -4.34 | -3.14 | -3.50 | -4.20 | -4.01 | -2.80 | -3.35 | -3.66 | -3.57 | -3.04 | -2.68 | -2.41 | -2.44 | -3.09 | -3.28 | -3.00 | -2.53 | -4.49 |
| STAT6 | -3.23 | -3.91 | -2.73 | -4.15 | -3.96 | -3.97 | -4.52 | -5.31 | -4.78 | -3.12 | -3.81 | -3.92 | -4.06 | -2.58 | -2.76 | -2.81 | -2.46 | -2.77 | -3.68 | -2.76 | -2.77 | -2.44 |
| TBX21 | -8.47 | -4.02 | -7.12 | NA | -5.22 | -7.83 | -5.02 | -6.19 | -5.64 | -4.48 | -6.70 | -6.16 | -6.23 | -4.99 | -7.40 | -7.89 | -5.27 | -8.27 | -5.05 | -6.77 | -5.09 | -5.52 |
| TCF7 | -0.25 | -1.45 | -0.93 | -2.05 | -2.24 | -1.95 | -1.83 | -2.40 | -2.27 | -1.42 | -1.59 | -3.17 | -2.14 | -0.64 | -0.55 | -0.60 | -0.55 | -0.83 | -1.17 | -0.88 | -0.56 | -4.06 |
| TGFB1 | -2.25 | -1.88 | -1.57 | -1.76 | -1.56 | -1.08 | -0.74 | -2.42 | -1.78 | -1.28 | -1.43 | -2.06 | -3.18 | -2.27 | -1.78 | -1.55 | -1.15 | -1.86 | -1.74 | -2.21 | -1.60 | -2.29 |
| TNF | -5.49 | -4.89 | -5.65 | -4.22 | -4.96 | -4.43 | -5.02 | -5.91 | -5.42 | -5.58 | -4.70 | -5.93 | -5.88 | -5.66 | -4.89 | -5.36 | -6.14 | -5.63 | -5.43 | -4.80 | -4.99 | -4.72 |
| TNFRSF4 | ### | -6.84 | -6.49 | -6.33 | -5.73 | -6.54 | -4.90 | NA | -7.36 | -6.20 | -5.84 | -6.84 | -7.08 | -6.74 | -6.40 | -5.84 | -5.81 | -7.48 | -6.08 | -5.61 | -6.17 | NA |
| TRAF3IP2 | -5.37 | -5.90 | -5.66 | -5.99 | -6.07 | -5.89 | -5.63 | -7.26 | -6.61 | -6.38 | -6.26 | -6.11 | -5.25 | -5.32 | -5.25 | -5.52 | -5.56 | -5.49 | -6.37 | -4.86 | -4.95 | -5.31 |
| VAV1 | -4.46 | -4.67 | -4.16 | -5.28 | -5.06 | -4.94 | -3.77 | -7.78 | -5.90 | -4.58 | -5.01 | -5.37 | -5.23 | -4.56 | -4.67 | -4.42 | -4.47 | -4.42 | -4.66 | -3.98 | -3.95 | -4.16 |

Expression values for each gene are represented as Z-scores, obtained from the mean of all the technical and biological replicates normalized by GAPDH expression**.** Abbreviations (alphabetical order): NA: no amplification; TFH: follicular helper T cell; Th: T-helper; Treg: regulatory T cell.

| -11.0 |  |  |  |  |  |  |  |  |  |  | 4.0 |
| --- | --- | --- | --- | --- | --- | --- | --- | --- | --- | --- | --- |

*Z-score (color code)*

**Supplementary Table 8 –** Reproducibility of conventional manual analysis and database-guided automated analysis for the identification of cell populations in EDTA-anticoagulated PB samples (n=5) stained with the final version of the EuroFlow-IMM T CD4 tube.

|  |  | E1 (1st round) vs E2 (1st round) | | E1 (1st round) vs E1 (2nd round) | | E1 (1st round) vs DB (1st round) | |
| --- | --- | --- | --- | --- | --- | --- | --- |
| **Major cell populations identified** | Median %* | r^2^ | MNB (%) | r^2^ | MNB (%) | r^2^ | MNB (%) |
| Nucleated cells | 100 | 0.998 | -1 | 0.999 | -1 | 1 | 0.2 |
| Total Lymphocytes | 42 (31-53) | 1 | 0.01 | 0.999 | -1 | 1 | 3 |
| T Lymphocytes | 24 (21-40) | 0.999 | 1 | 0.999 | -1 | 1 | 2 |
| CD4+ T cells | 15 (13-24) | 0.998 | 2 | 0.998 | -0.5 | 0.998 | 3 |
| CD4+ naïve T cells | 6.0 (2.5-7.3) | 0.988 | 11 | 0.974 | 2 | 0.97 | 14 |
| CD4+ Th1 cells | 1.8 (0.7-4.3) | 0.978 | 22 | 0.997 | -0.1 | 0.987 | 9 |
| CD4+ Th2 cells | 0.7 (0.4-1.1) | 0.951 | -5 | 0.083 | -24 | 0.085 | 5 |
| CD4+ Th17 cells | 0.6 (0.3-0.9) | 0.842 | 13 | 0.961 | -2 | 0.796 | 22 |
| CD4+ Th1/Th17 cells | 0.7 (0.5-2.4) | 0.987 | 12 | 0.977 | -4 | 0.988 | 2 |
| CD4+ Th22 cells | 0.2 (0.1-0.3) | 0.945 | 27 | 0.987 | 3 | 0.983 | 17 |
| CD4+ Tregs | 1.0 (0.5-1.2) | 0.897 | 6 | 0.967 | 6 | 0.99 | 3 |
| CD4+ TFH cells | 3.0 (2.0-5.0) | 0.991 | -10 | 0.994 | -14 | 0.998 | 9 |
| CD4- T cells | 9.7 (6.0-16) | 0.999 | 2 | 1 | -0.05 | 1 | 2 |
| % of populations with r^2^ ≥ 0.9 and p ≤ 0.05  or -15% < MNB < +15%: |  | **11/13 (85%)** | **11/13 (85%)** | **12/13 (92%)** | **12/13 (92%)** | **11/13 (85%)** | **11/13 (85%)** |
|  |  |  |  |  |  |  |  |
|  |  |  |  |  |  |  |  |
|  |  | E1 (1st round) vs E2 (1st round) | | E1 (1st round) vs E1 (2nd round) | | E1 (1st round) vs DB (1st round) | |
| **Classical CD4+ Th cell subsets identified** | Median %* | r^2^ | MNB (%) | r^2^ | MNB (%) | r^2^ | MNB (%) |
| CD4+ Th1 cells | 1.8 (0.7-4.3) | 0.978 | 22 | 0.997 | -0.1 | 0.987 | 9 |
| Central memory Th1 | 0.9 (0.6-1.8) | 0.992 | 27 | 0.973 | -1.3 | 0.97 | 6 |
| Transitional memory Th1 | 0.2 (0.1-0.4) | 0.997 | 36 | 0.985 | -4 | 0.948 | 12 |
| Effector memory Th1 | 0.2 (0.01-0.5) | 0.968 | 41 | 0.993 | -0.3 | 0.1 | -42 |
| Terminal effector Th1 | 0.03 (<0.01-2.6) | ND | ND | ND | ND | ND | ND |

**Supplementary Table 8** *(Continued)*

|  |  | E1 (1st round) vs E2 (1st round) | | E1 (1st round) vs E1 (2nd round) | | E1 (1st round) vs DB (1st round) | |
| --- | --- | --- | --- | --- | --- | --- | --- |
| **Classical CD4+ Th cell subsets identified** | Median %* | r^2^ | MNB (%) | r^2^ | MNB (%) | r^2^ | MNB (%) |
| CD4+ Th2 cells | 0.7 (0.4-1.1) | 0.951 | -5 | 0.083 | -24 | 0.085 | -5 |
| Central memory Th2 | 0.7 (0.4-1.0) | 0.959 | -7 | 0.093 | -24 | 0.153 | -9 |
| Transitional memory Th2 | 0.01 (<0.01-0.01) | 0.502 | 43 | 0.431 | -23 | 0.37 | 18 |
| Effector memory Th2 | 0.05 (0.02-0.06) | 0.963 | 20 | 0.948 | -10 | 0.647 | 9 |
| Terminal effector Th2 | <0.01 (<0.01-0.01) | ND | ND | ND | ND | ND | ND |
| CD4+ Th17 cells | 0.6 (0.3-0.9) | 0.842 | 13 | 0.961 | -2 | 0.796 | 22 |
| Central memory Th17 | 0.4 (0.2-0.7) | 0.454 | 14 | 0.961 | -2 | 0.855 | 22 |
| Transitional memory Th17 | 0.1 (0.04-0.1) | 0.532 | 16 | 0.875 | -0.4 | 0.454 | 40 |
| Effector memory Th17 | 0.04 (0.02-0.06) | 0.252 | 15 | 0.893 | -3 | 0.293 | 2 |
| Terminal effector Th17 | <0.01 | ND | ND | ND | ND | ND | ND |
| CD4+ Th1/Th17 cells | 0.7 (0.5-2.4) | 0.987 | 12 | 0.977 | -4 | 0.988 | 2 |
| Central memory Th1/Th17 | 0.3 (0.3-1.4) | 0.985 | 18 | 0.865 | -15 | 0.917 | -15 |
| Transitional memory Th1/Th17 | 0.3 (0.2-0.9) | 0.99 | 5 | 0.884 | -22 | 0.998 | 21 |
| Effector memory Th1/Th17 | 0.1 (0.01-0.2) | 0.946 | 25 | 0.576 | -12 | 0.972 | 41 |
| Terminal effector Th1/Th17 | <0.01 | ND | ND | ND | ND | ND | ND |
| CD4+ Th22 cells | 0.2 (0.07-0.3) | 0.945 | 27 | 0.987 | -3 | 0.983 | 17 |
| Central memory Th22 | 0.05 (0.03-0.1) | 0.927 | 20 | 0.923 | -2 | 0.912 | 6 |
| Transitional memory Th22 | 0.07 (0.02-0.1) | 0.889 | 28 | 0.944 | -0.5 | 0.953 | -8 |
| Effector memory Th22 | 0.08 (0.02-0.1) | 0.986 | 35 | 0.98 | -8 | 0.99 | 47 |
| Terminal effector Th22 | <0.01 | ND | ND | ND | ND | ND | ND |
| % of populations with r^2^ ≥ 0.9 and p ≤ 0.05 |  | **19/25 (76%)** | **11/25 (44%)** | **17/25 (68%)** | **20/25 (80%)** | **16/25 (64%)** | **15/25 (60%)** |
| or -15% < MNB < +15%: |  |  |  |  |  |  |  |

**Supplementary Table 8** *(Continued)*

|  |  | E1 (1st round) vs E2 (1st round) | | E1 (1st round) vs E1 (2nd round) | | E1 (1st round) vs DB (1st round) | |
| --- | --- | --- | --- | --- | --- | --- | --- |
| **Other Th subsets:** | Median %* | r^2^ | MNB (%) | r^2^ | MNB (%) | r^2^ | MNB (%) |
| CD4+ CD183+ CD194+ CD196+ CCR10+ | 0.2 (0.2-0.6) | 0.989 | -11 | 0.986 | 6 | 0.979 | -6 |
| CM CD4+ CD183+ CD194+ CD196+ CCR10+ | 0.06 (0.05-0.3) | 0.994 | -11 | 0.996 | 5 | 0.911 | -37 |
| TM CD4+ CD183+ CD194+ CD196+ CCR10+ | 0.08 (0.07-0.3) | 0.994 | -10 | 0.987 | 4 | 0.865 | 21 |
| EM CD4+ CD183+ CD194+ CD196+ CCR10+ | 0.06 (0.05-0.08) | 0.852 | -10 | 0.81 | 7 | 0.451 | -22 |
| TE CD4+ CD183+ CD194+ CD196+ CCR10+ | <0.01 | ND | ND | ND | ND | ND | ND |
| CD4+ CD183+ CD194+ CD196+ CCR10- | 0.8 (0.4-1.8) | 0.968 | -27 | 0.982 | 7 | 0.986 | -3 |
| CM CD4+ CD183+ CD194+ CD196+ CCR10- | 0.5 (0.2-1.2) | 0.963 | -26 | 0.99 | 8 | 0.975 | 2 |
| TM CD4+ CD183+ CD194+ CD196+ CCR10- | 0.2 (0.1-0.5) | 0.991 | -31 | 0.974 | 6 | 0.825 | -10 |
| EM CD4+ CD183+ CD194+ CD196+ CCR10- | 0.1 (0.07-0.2) | 0.496 | -27 | 0.45 | 4 | 0.625 | -18 |
| TE CD4+ CD183+ CD194+ CD196+ CCR10- | <0.01 | ND | ND | ND | ND | ND | ND |
| CD4+ CD183+ CD194+ CD196- CCR10+ | 0.08 (0.07-0.3) | 0.978 | -11 | 0.993 | -4 | 0.955 | -77 |
| CM CD4+ CD183+ CD194+ CD196- CCR10+ | 0.06 (0.04-0.2) | 0.989 | -14 | 0.996 | -4 | 0.914 | -71 |
| TM CD4+ CD183+ CD194+ CD196- CCR10+ | 0.02 (0.01-0.07) | 0.943 | -0.5 | 0.992 | -6 | 0.93 | -89 |
| EM CD4+ CD183+ CD194+ CD196- CCR10+ | 0.01 (<0.01-0.03) | 0.949 | 12 | 0.966 | -3 | 0.727 | -77 |
| TE CD4+ CD183+ CD194+ CD196- CCR10+ | <0.01 | ND | ND | ND | ND | ND | ND |
| CD4+ CD183+ CD194+ CD196- CCR10- | 1.1 (0.3-1.5) | 0.986 | -14 | 0.72 | 9 | 0.94 | 33 |
| CM CD4+ CD183+ CD194+ CD196- CCR10- | 0.6 (0.2-1.2) | 0.96 | -11 | 0.857 | 10 | 0.967 | 39 |
| TM CD4+ CD183+ CD194+ CD196- CCR10- | 0.2 (0.07-0.3) | 0.989 | -18 | 0.847 | 5 | 0.648 | 20 |
| EM CD4+ CD183+ CD194+ CD196- CCR10- | 0.09 (0.04-0.2) | 0.879 | -17 | 0.655 | 6 | 0.011 | 10 |
| TE CD4+ CD183+ CD194+ CD196- CCR10- | 0.01 (<0.01-0.09) | ND | ND | ND | ND | ND | ND |
| CD4+ CD183+ CD194- CD196+ CCR10+ | 0.01 (<0.01-0.02) | ND | ND | ND | ND | ND | ND |
| CM CD4+ CD183+ CD194- CD196+ CCR10+ | <0.01 | ND | ND | ND | ND | ND | ND |
| TM CD4+ CD183+ CD194- CD196+ CCR10+ | <0.01 | ND | ND | ND | ND | ND | ND |
| EM CD4+ CD183+ CD194- CD196+ CCR10+ | <0.01 | ND | ND | ND | ND | ND | ND |
| TE CD4+ CD183+ CD194- CD196+ CCR10+ | <0.01 | ND | ND | ND | ND | ND | ND |

**Supplementary Table 8** *(Continued)*

|  |  | E1 (1st round) vs E2 (1st round) | | E1 (1st round) vs E1 (2nd round) | | E1 (1st round) vs DB (1st round) | |
| --- | --- | --- | --- | --- | --- | --- | --- |
| **Other Th subsets:** | Median %* | r^2^ | MNB (%) | r^2^ | MNB (%) | r^2^ | MNB (%) |
| CD4+ CD183+ CD194- CD196- CCR10+ | 0.02 (<0.01-0.07) | 0.936 | 15 | 0.99 | 43 | 0.871 | -59 |
| CM CD4+ CD183+ CD194- CD196- CCR10+ | <0.01 (<0.01-0.04) | 0.972 | 37 | 0.949 | 113 | 0.982 | -73 |
| TM CD4+ CD183+ CD194- CD196- CCR10+ | <0.01 (<0.01-0.01) | ND | ND | ND | ND | ND | ND |
| EM CD4+ CD183+ CD194- CD196- CCR10+ | <0.01 (<0.01-0.02) | 0.917 | 5 | 0.997 | -25 | 0.81 | -36 |
| TE CD4+ CD183+ CD194- CD196- CCR10+ | <0.01 | ND | ND | ND | ND | ND | ND |
| CD4+ CD183- CD194- CD196+ CCR10- | 0.1 (0.04-0.1) | 0.363 | 135 | 0.378 | 55 | 0.28 | -48 |
| CM CD4+ CD183- CD194- CD196+ CCR10- | 0.09 (0.04-0.1) | 0.284 | 119 | 0.281 | 59 | 0.214 | -42 |
| TM CD4+ CD183- CD194- CD196+ CCR10- | 0.01 (<0.01-0.02) | 0.792 | 335 | 0.849 | 44 | 0.541 | -75 |
| EM CD4+ CD183- CD194- CD196+ CCR10- | 0.01 (<0.01-0.01) | ND | ND | ND | ND | ND | ND |
| TE CD4+ CD183- CD194- CD196+ CCR10- | <0.01 | ND | ND | ND | ND | ND | ND |
| CD4+ CD183- CD194+ CD196- CCR10+ | 0.07 (0.06-0.1) | 0.979 | 20 | 0.932 | 10 | 0.945 | 5 |
| CM CD4+ CD183- CD194+ CD196- CCR10+ | 0.06 (0.05-0.09) | 0.968 | 18 | 0.89 | 11 | 0.857 | 31 |
| TM CD4+ CD183- CD194+ CD196- CCR10+ | <0.01 | 0.477 | 102 | 0.249 | 25 | ND | ND |
| EM CD4+ CD183- CD194+ CD196- CCR10+ | 0.01 (<0.01-0.05) | 0.992 | 20 | 0.983 | 3 | 0.93 | -80 |
| TE CD4+ CD183- CD194+ CD196- CCR10+ | <0.01 | ND | ND | ND | ND | ND | ND |
| non-naïve CD4+ CD183- CD194- CD196-CCR10- | 0.1 (0.02-0.4) | 0 | 86 | 0.237 | 208 | ND | ND |
| % of populations with r^2^ ≥ 0.9 and p ≤ 0.05 |  | **34/41 (83%)** | **25/41 (61%)** | **29/41 (71%)** | **33/41 (80%)** | **28/41 (68%)** | **22/41 (54%)** |
| or -15% < MNB < +15% |  |  |  |  |  |  |  |
|  |  | E1 (1st round) vs E2 (1st round) | | E1 (1st round) vs E1 (2nd round) | | E1 (1st round) vs DB (1st round) | |
| **Treg and TFH subsets:** | Median %* | r^2^ | MNB (%) | r^2^ | MNB (%) | r^2^ | MNB (%) |
| CD4+ Tregs | 0.9 (0.5-1.2) | 0.897 | 6 | 0.967 | 6 | 0.99 | 3 |
| Naïve Treg | 0.2 (0.03-0.4) | 0.933 | 30 | 0.902 | 19 | 0.98 | 4 |
| Th1-like Treg | 0.03 (0.01-0.05) | 0.78 | 62 | 0.813 | 25 | 0.745 | 91 |
| Th2-like Treg | 0.02 (0.02-0.05) | 0.111 | 71 | 0.69 | 33 | 0.206 | -50 |
| Th17-like Treg | 0.1 (0.09-0.1) | 0.507 | 26 | 0.947 | -0.2 | 0.761 | 27 |
| Th22-like Treg | 0.1 (0.05-0.2) | 0.959 | 3 | 0.988 | 1 | 0.881 | -17 |

**Supplementary Table 8** *(Continued)*

|  |  | E1 (1st round) vs E2 (1st round) | | E1 (1st round) vs E1 (2nd round) | | E1 (1st round) vs DB (1st round) | |
| --- | --- | --- | --- | --- | --- | --- | --- |
| **Treg and TFH subsets:** | Median %* | r^2^ | MNB (%) | r^2^ | MNB (%) | r^2^ | MNB (%) |
| CD183+ CD194+ CD196- CCR10+ Treg | 0.02 (0.01-0.04) | 0.953 | -19 | 0.939 | -17 | 0.517 | -87 |
| CD183+ CD194+ CD196- CCR10- Treg | 0.04 (0.03-0.06) | 0.501 | 25 | 0.892 | 1 | 0.709 | -40 |
| CD183+ CD194+ CD196+ CCR10- Treg | 0.1 (0.09-0.2) | 0.857 | -3 | 0.971 | 1 | 0.829 | 7 |
| CD183+ CD194+ CD196+ CCR10+ Treg | 0.1 (0.06-0.2) | 0.587 | -24 | 0.307 | 2 | 0.015 | 3 |
| CD183- CD194+ CD196- CCR10+ Treg | 0.01 (<0.01-0.03) | 0.848 | -3 | 0.88 | -16 | 0.807 | -85 |
| CD4+ TFH cells | 3.0 (1.7-4.9) | 0.991 | -10 | 0.996 | -14 | 0.998 | -9 |
| Treg TFH | 0.2 (0.1-0.3) | 0.77 | -10 | 0.86 | -32 | 0.428 | -68 |
| Th1-like TFH | 0.3 (0.2-1.0) | 0.994 | 10 | 0.987 | -30 | 0.95 | -2 |
| Th2-like TFH | 0.2 (0.1-0.3) | 0.967 | -16 | 0.945 | -30 | 0.789 | -20 |
| Th17-like TFH | 0.8 (0.3-0.9) | 0.992 | -11 | 0.984 | -11 | 0.917 | 20 |
| Th1/Th17-like TFH | 0.2 (0.08-0.3) | 0.965 | 1 | 0.915 | -17 | 0.267 | -3 |
| CD183+ CD194+ CD196- CCR10- TFH | 0.4 (0.2-0.7) | 0.973 | -19 | 0.992 | -12 | 0.898 | 25 |
| CD183+ CD194+ CD196+ CCR10- TFH | 0.3 (0.2-0.6) | 0.935 | -40 | 0.981 | 3 | 0.811 | -53 |
| CD183- CD194- CD196+ CCR10- TFH | 0.2 (0.1-0.3) | 0.864 | 27 | 0.801 | 10 | 0.55 | 22 |
| CD183- CD194- CD196- CCR10- TFH | 0.2 (0.07-0.3) | 0.073 | -91 | 0.906 | 22 | 0.581 | -3 |
| CD4+ CD185+ CD27+ CD45RA+ CD62L+ T cells | 0.1 (0.1-0.3) | 0.882 | 99 | 0.898 | -52 | 0.523 | -42 |
| % of populations with r^2^ ≥ 0.9 and p ≤ 0.05 |  | **10/22 (45%)** | **9/22 (41%)** | **14/22 (64%)** | **11/22 (50%)** | **6/22 (27%)** | **8/22 (36%)** |
| or -15% < MNB < +15% |  |  |  |  |  |  |  |

For comparisons between database-guided automated analyses in two rounds (e.g. performed at different time-points), high correlation (r^2^ ≥ 0.9; *p*-value ≥ 0.05) and high agreement (-15% < mean normalized bias (MNB) < +15%) were obtained for 100% of the cell populations identified. Bias was normalized with the number of events (cells) obtained by E1 and MNB results represented as percentages of (degree of) agreement between manual and automated analysis when compared to E1 (1^st^ round) (MNB was considered acceptable when its value was ±15% the number of events identified by E1 in the 1^st^ round). Squared Pearson correlation coefficients (r^2^) were calculated with SPSS (v.23.0; SPSS-IBM, Armonk, NY) and the Bland and Altman method (XLSTAT 2019, Addinsoft, Paris, France) was used to calculate MNB. Values marked in red represent r^2^ < 0.9 or -15% > MNB > +15%. *Median % of cells within nucleated cells as identified by E1 (1^st^ round). **Abbreviations** (alphabetical order): DB, database-guided automated analysis; E1, experienced cytometrist 1; E2, begginer cytometrist 2; MNB, mean normalized bias (mean % of events underestimated (-)/overestimated (+) by other methods/operators compared to E1 results in the first round of analysis); ND: no data (population was not identified by one or the two methods).

**Supplementary Table 9 -** Percentage of blood samples in which the distinct CD4+ T-cell populations identified were detected and their corresponding absolute counts per age group, as identified with the EuroFlow-IMM TCD4 tube (version 3).

|  | **CB (n=15)** | **<2 y (n=2)** | **2-4 y (n=6)** | **5-9 y (n=12)** | **10-17 y (n=13)** | **18-39 y (n=12)** | **40-59 y (n=17)** | **60-79 y (n=22)** | **>80 y (n=14)** |
| --- | --- | --- | --- | --- | --- | --- | --- | --- | --- |
| **Total T cells** |  |  |  |  |  |  |  |  |  |
| % blood samples | 100% | 100% | 100% | 100% | 100% | 100% | 100% | 100% | 100% |
| Cells/μL | 3325 (1744-4375) | 4263 (4248-4279) | 1996 (1069-4596) | 2499 (1296-4743) | 1677 (1221-2194) | 1430 (863-2160) | 1756 (978-3414) | 1839 (1043-3418) | 1531 (551-3379) |
| **CD4+ T cells** |  |  |  |  |  |  |  |  |  |
| % blood samples | 100% | 100% | 100% | 100% | 100% | 100% | 100% | 100% | 100% |
| Cells/μL | 2155 (1209-2692) | 2720 (2193-3248) | 936 (531-3503) | 1474 (711-2238) | 973 (573-1308) | 847 (515-1203) | 1119 (582-2628) | 1134 (684-2587) | 963 (330-1719) |
| **CD4+ naïve T cells** |  |  |  |  |  |  |  |  |  |
| % blood samples | 100% | 100% | 100% | 100% | 100% | 100% | 100% | 100% | 100% |
| Cells/μL | 1873 (1089-2424) | 1973 (1399-2548) | 543 (275-2712) | 977 (365-1612) | 566 (234-871) | 380 (149-690) | 343 (147-1432) | 347 (46-923) | 271 (27-589) |
| **CD4+ Th1 cells** |  |  |  |  |  |  |  |  |  |
| % blood samples | 100% | 100% | 100% | 100% | 100% | 100% | 100% | 100% | 100% |
| Cells/μL | 19 (9.8-36) | 141 (139-142) | 67 (31-134) | 62 (41-176) | 39 (32-149) | 59 (27-116) | 129 (53-704) | 142 (45-587) | 177 (88-703) |
| **CD4+ Th2 cells** |  |  |  |  |  |  |  |  |  |
| % blood samples | 100% | 100% | 100% | 100% | 100% | 100% | 100% | 100% | 100% |
| Cells/μL | 47 (26-119) | 70 (60-80) | 24 (11-52) | 30 (15-85) | 26 (18-39) | 26 (16-70) | 49 (24-123) | 80 (25-220) | 57 (29-87) |
| **CD4+ Th17 cells** |  |  |  |  |  |  |  |  |  |
| % blood samples | 100% | 100% | 100% | 100% | 100% | 100% | 100% | 100% | 100% |
| Cells/μL | 4.6 (2.4-15) | 40 (33-46) | 23 (7.5-46) | 29 (14-58) | 24 (10-36) | 31 (14-45) | 37 (14-93) | 44 (21-120) | 39 (13-86) |
| **CD4+ Th1/17 cells** |  |  |  |  |  |  |  |  |  |
| % blood samples | 6.7% | 100% | 100% | 100% | 100% | 100% | 100% | 100% | 100% |
| Cells/μL | <0.01 (<0.01-0.8) | 6.6 (6.4-6.7) | 9.2 (6.3-58) | 28 (14-110) | 33 (14-71) | 52 (23-115) | 60 (20-124) | 58 (12-236) | 38 (6-121) |
| **CD4+ Th22 cells** |  |  |  |  |  |  |  |  |  |
| % blood samples | 0% | 100% | 100% | 100% | 100% | 100% | 100% | 100% | 100% |
| Cells/μL | <0.01 | 1.5 (0.9-2.2) | 2.6 (0.5-7.9) | 4.0 (2.5-14) | 6.3 (1.0-17) | 11 (6.96-22) | 15 (3-38) | 12 (3-40) | 9 (3-23) |
| **CD4+ Tregs** |  |  |  |  |  |  |  |  |  |
| % blood samples | 100% | 100% | 100% | 100% | 100% | 100% | 100% | 100% | 100% |
| Cells/μL | 133 (66-213) | 242 (188-297) | 78 (48-290) | 120 (49-192) | 67 (33-97) | 50 (30-95) | 62 (22-141) | 61 (31-132) | 50 (19-115) |
| **CD4+ TFH cells** |  |  |  |  |  |  |  |  |  |
| % blood samples | 33.3% | 100% | 100% | 100% | 100% | 100% | 100% | 100% | 100% |
| Cells/μL | <0.01 (<0.01-41) | 101 (23-179) | 78 (42-173) | 119 (72-154) | 76 (42-127) | 100 (55-150) | 111 (45-240) | 163 (68-325) | 113 (33-206) |
| **CD4- T cells** |  |  |  |  |  |  |  |  |  |
| % blood samples | 100% | 100% | 100% | 100% | 100% | 100% | 100% | 100% | 100% |
| Cells/μL | 981 (536-1684) | 1543 (1000-2086) | 933 (538-1113) | 979 (584-2627) | 788 (486-967) | 499 (348-1136) | 644 (367-1413) | 666 (263-1975) | 620 (221-2408) |
|  | | | | | | | | | |

**Supplementary Table 9** *(Continued)*

|  | **CB (n=15)** | **<2 y (n=2)** | **2-4 y (n=6)** | **5-9 y (n=12)** | **10-17 y (n=13)** | **18-39 y (n=12)** | **40-59 y (n=17)** | **60-79 y (n=22)** | **>80 y (n=14)** |
| --- | --- | --- | --- | --- | --- | --- | --- | --- | --- |
| **CD4+ Th1 cells** |  |  |  |  |  |  |  |  |  |
| % blood samples | 100% | 100% | 100% | 100% | 100% | 100% | 100% | 100% | 100% |
| Cells/μL | 19 (9.8-36) | 141 (139-143) | 67 (31-134) | 62 (41-176) | 39 (32-149) | 59 (27-116) | 129 (53-704) | 142 (45-587) | 177 (88-703) |
| **Central memory Th1** |  |  |  |  |  |  |  |  |  |
| % blood samples | 100% | 100% | 100% | 100% | 100% | 100% | 100% | 100% | 100% |
| Cells/μL | 16 (6.7-32) | 114 (93-136) | 54 (22-98) | 48 (36-94) | 30 (23-58) | 35 (15-72) | 48 (12-277) | 64 (24-120) | 55 (13-199) |
| **Transitional memory Th1** |  |  |  |  |  |  |  |  |  |
| % blood samples | 100% | 100% | 100% | 100% | 100% | 100% | 100% | 100% | 100% |
| Cells/μL | 3.2 (2.1-7.4) | 11 (6.1-17) | 6.0 (5.0-27) | 6.8 (3.4-17) | 6.7 (3.9-17) | 11 (3.5-16) | 19 (3.6-81) | 11 (4.1-53) | 6.9 (1.8-50) |
| **Effector memory Th1** |  |  |  |  |  |  |  |  |  |
| % blood samples | 0% | 100% | 100% | 100% | 100% | 100% | 100% | 100% | 100% |
| Cells/μL | <0.01* | 14 (0.2-27) | 4.2 (0.6-8.9) | 2.8 (0.3-71) | 3.0 (0.2-100) | 4.7 (0.7-22) | 21 (2.6-428) | 34 (0.3-184) | 50 (11-343) |
| **Terminal effector Th1** |  |  |  |  |  |  |  |  |  |
| % blood samples | 0% | 0% | 16.7% | 0% | 7.7% | 100% | 100% | 100% | 92.9% |
| Cells/μL | <0.01* | <0.01 | 0.0 (<0.01-2.2) | <0.01 | <0.01 (<0.01-4.2) | 1.1 (0.02-26) | 5.9 (0.1-178) | 11 (0.1-414) | 36 (<0.01-255) |
| **CD4+ Th2 cells** |  |  |  |  |  |  |  |  |  |
| % blood samples | 100% | 100% | 100% | 100% | 100% | 100% | 100% | 100% | 100% |
| Cells/μL | 47 (26-119) | 71 (60-81) | 24 (11-52) | 30 (15-85) | 26 (18-39) | 26 (16-70) | 49 (24-123) | 80 (25-220) | 57 (29-87) |
| **Central memory Th2** |  |  |  |  |  |  |  |  |  |
| % blood samples | 100% | 100% | 100% | 100% | 100% | 100% | 100% | 100% | 100% |
| Cells/μL | 41 (26-105) | 63 (57-69) | 19 (9.3-45) | 26 (12-58) | 22 (12-26) | 23 (12-64) | 39 (9.4-106) | 70 (22-215) | 50 (20-76) |
| **Transitional memory Th2** |  |  |  |  |  |  |  |  |  |
| % blood samples | 80% | 100% | 100% | 100% | 100% | 100% | 100% | 100% | 100% |
| Cells/μL | 6.2 (<0.01-18) | 2.9 (2.9-3.0) | 2.0 (0.5-5.5) | 1.2 (0.5-6.0) | 1.1 (0.3-5.7) | 1.1 (0.2-3.5) | 2.7 (0.4-38) | 1.8 (0.6-5.3) | 1.0 (0.4-20) |
| **Effector memory Th2** |  |  |  |  |  |  |  |  |  |
| % blood samples | 0% | 50% | 100% | 100% | 100% | 100% | 100% | 100% | 100% |
| Cells/μL | <0.01 | 1.8 (<0.01-3.6) | 2.4 (1.2-3.8) | 3.1 (0.2-22) | 4.1 (0.9-14) | 2.6 (0.9-3.7) | 4.1 (1.4-14) | 4.5 (1.1-13) | 4.8 (1.3-10) |
| **Terminal effector Th2** |  |  |  |  |  |  |  |  |  |
| % blood samples | 0% | 50% | 0% | 0% | 0% | 77.7% | 64.7% | 86.4% | 71.4% |
| Cells/μL | <0.01 | 2.2 (<0.01-4.5) | <0.01 | <0.01 | <0.01 | 0.2 (<0.01-2.6) | 0.08 (<0.01-5.3) | 0.5 (<0.01-7.0) | 0.1 (<0.01-1.7) |
| **CD4+ Th17 cells** |  |  |  |  |  |  |  |  |  |
| % blood samples | 100% | 100% | 100% | 100% | 100% | 100% | 100% | 100% | 100% |
| Cells/μL | 4.6 (2.4-15) | 39 (33-46) | 23 (7.5-46) | 29 (14-58) | 24 (10-36) | 31 (14-45) | 37 (14-93) | 44 (21-120) | 39 (13-86) |
| **Central memory Th17** |  |  |  |  |  |  |  |  |  |
| % blood samples | 100% | 100% | 100% | 100% | 100% | 100% | 100% | 100% | 100% |
| Cells/μL | 3.0 (0.9-7.7) | 33 (27-39) | 15 (5.7-32) | 19 (9.6-40.6) | 16 (6.7-23) | 19 (10-28) | 27 (2.1-61) | 35 (14-94) | 30 (8.9-65) |
| **Transitional memory Th17** |  |  |  |  |  |  |  |  |  |
| % blood samples | 100% | 100% | 100% | 100% | 100% | 100% | 100% | 100% | 100% |
| Cells/μL | 2.5 (0.8-8.3) | 4.9 (3.6-6.2) | 4.7 (1.3-12.6) | 4.4 (2.4-10.7) | 4.0 (1.9-7.5) | 5.5 (2.6-14) | 8.6 (2.0-26) | 7.2 (1.6-21) | 5.9 (2.5-19) |

**Supplementary Table 9** *(Continued)*

|  | **CB (n=15)** | **<2 y (n=2)** | **2-4 y (n=6)** | **5-9 y (n=12)** | **10-17 y (n=13)** | **18-39 y (n=12)** | **40-59 y (n=17)** | **60-79 y (n=22)** | **>80 y (n=14)** |
| --- | --- | --- | --- | --- | --- | --- | --- | --- | --- |
| **Effector memory Th17** |  |  |  |  |  |  |  |  |  |
| % blood samples | 0% | 100% | 100% | 100% | 100% | 100% | 100% | 100% | 100% |
| Cells/μL | <0.01 | 1.7 (1.0-2.4) | 1.9 (0.5-2.9) | 4.0 (2.0-7.0) | 4.2 (1.3-7.4) | 3.2 (1.4-5.7) | 2.8 (1.4-8.4) | 2.9 (0.8-8.4) | 2.7 (0.9-12) |
| **Terminal effector Th17** |  |  |  |  |  |  |  |  |  |
| % blood samples | 0% | 0% | 0% | 0% | 0% | 58.3% | 35.3% | 72.7% | 14.3% |
| Cells/μL | <0.01 | <0.01 | <0.01 | <0.01 | <0.01 | 0.02 (<0.01-1.8) | <0.01 (<0.01-0.4) | 0.1 (<0.01-1.2) | <0.01 (<0.01-0.06) |
| **CD4+ Th1/Th17 cells** |  |  |  |  |  |  |  |  |  |
| % blood samples | 6.7% | 100% | 100% | 100% | 100% | 100% | 100% | 100% | 100% |
| Cells/μL | <0.01 (<0.01-0.8) | 6.6 (6.4-6.7) | 9.2 (6.3-58) | 28 (14-110) | 33 (14-71) | 52 (23-115) | 60 (20-124) | 58 (12-236) | 38 (6.6-121) |
| **Central memory Th1/Th17** |  |  |  |  |  |  |  |  |  |
| % blood samples | 6.7% | 100% | 100% | 100% | 100% | 100% | 100% | 100% | 100% |
| Cells/μL | <0.01 (<0.01-0.8) | 5.2 (4.5-5.9) | 6.1 (4.1-31) | 17 (5.4-70) | 18 (6.9-38) | 22 (11-43) | 23 (5.0-71) | 25 (4.5-75) | 15 (1.2-59) |
| **Transitional memory Th1/Th17** |  |  |  |  |  |  |  |  |  |
| % blood samples | 0% | 100% | 100% | 100% | 100% | 100% | 100% | 100% | 100% |
| Cells/μL | <0.01 | 1.2 (0.3-2.0) | 2.2 (1.1-19.4) | 7.5 (4.2-28) | 10 (5.4-30) | 21 (9.0-59) | 29 (6.2-61) | 22 (5.0-106) | 13 (2.5-49) |
| **Effector memory Th1/Th17** |  |  |  |  |  |  |  |  |  |
| % blood samples | 0% | 50% | 100% | 100% | 100% | 100% | 100% | 100% | 100% |
| Cells/μL | <0.01 | 0.05 (<0.01-0.09) | 0.8 (0.4-6.8) | 3.4 (1.4-12) | 5.3 (1.0-17) | 5.1 (0.7-11) | 7.0 (2.1-16) | 8.0 (1.1-148) | 4.2 (0.7-41) |
| **Terminal effector Th1/Th17** |  |  |  |  |  |  |  |  |  |
| % blood samples | 0% | 0% | 0% | 0% | 0% | 91.7% | 64.7% | 86.4% | 78.6% |
| Cells/μL | <0.01 | <0.01 | <0.01 | <0.01 | <0.01 | 0.4 (<0.01-1.6) | 0.08 (<0.01-1.0) | 0.3 (<0.01-4.5) | 0.1 (<0.01-0.5) |
| **CD4+ Th22 cells** |  |  |  |  |  |  |  |  |  |
| % blood samples | 0% | 100% | 100% | 100% | 100% | 100% | 100% | 100% | 100% |
| Cells/μL | <0.01 | 1.5 (0.9-2.2) | 2.6 (0.5-7.9) | 4.0 (2.5-14) | 6.3 (1.0-17) | 11 (7.0-22) | 15 (3.6-38) | 12 (3.3-40) | 9.9 (3.2-23) |
| **Central memory Th22** |  |  |  |  |  |  |  |  |  |
| % blood samples | 0% | 100% | 100% | 100% | 100% | 100% | 100% | 100% | 100% |
| Cells/μL | <0.01 | 1.3 (0.7-1.9) | 1.0 (0.2-3.8) | 1.4 (0.8-3.7) | 1.7 (0.3-7.2) | 3.3 (1.6-7.2) | 4.6 (0.3-12) | 4.0 (1.4-15) | 2.9 (1.2-9.1) |
| **Transitional memory Th22** |  |  |  |  |  |  |  |  |  |
| % blood samples | 0% | 50% | 100% | 100% | 100% | 100% | 100% | 100% | 100% |
| Cells/μL | <0.01 | 0.2 (<0.01-0.3) | 0.8 (0.2-3.0) | 1.3 (0.4-5.1) | 1.7 (0.5-5.6) | 3.6 (1.9-7.9) | 4.8 (1.2-14) | 4.7 (1.2-14) | 3.5 (1.3-6.6) |
| **Effector memory Th22** |  |  |  |  |  |  |  |  |  |
| % blood samples | 0% | 0% | 100% | 100% | 100% | 100% | 100% | 100% | 100% |
| Cells/μL | <0.01 | <0.01 | 0.7 (0.1-1.6) | 1.2 (0.7-4.5) | 2.3 (0.3-7.3) | 3.4 (1.6-7.2) | 5.1 (1.3-9.1) | 3.5 (0.4-12) | 2.7 (0.7-8.5) |
| **Terminal effector Th22** |  |  |  |  |  |  |  |  |  |
| % blood samples | 0% | 0% | 0% | 0% | 0% | 100% | 64.7% | 86.4% | 78.6% |
| Cells/μL | <0.01 | <0.01 | <0.01 | <0.01 | <0.01 | 0.5 (0.1-2.6) | 0.2 (<0.01-2.1) | 0.3 (<0.01-1.4) | 0.2 (<0.01-1.1) |

**Supplementary Table 9** *(Continued)*

|  | **CB (n=15)** | **<2 y (n=2)** | **2-4 y (n=6)** | **5-9 y (n=12)** | **10-17 y (n=13)** | **18-39 y (n=12)** | **40-59 y (n=17)** | **60-79 y (n=22)** | **>80 y (n=14)** |
| --- | --- | --- | --- | --- | --- | --- | --- | --- | --- |
| **CD4+ CD183+ CD194+ CD196+ CCR10+** |  |  |  |  |  |  |  |  |  |
| % blood samples | 0% | 50% | 100% | 100% | 100% | 100% | 100% | 100% | 100% |
| Cells/μL | <0.01 | 0.2 (<0.01-0.4) | 2.0 (0.2-7.8) | 3.5 (1.6-15) | 5.4 (1.8-10) | 7.8 (3.5-16) | 9.6 (2.8-35) | 8.0 (1.3-39) | 5.7 (3.2-25) |
| **CM CD4+ CD183+ CD194+ CD196+ CCR10+** |  |  |  |  |  |  |  |  |  |
| % blood samples | 0% | 50% | 83.30% | 100% | 100% | 100% | 100% | 100% | 100% |
| Cells/μL | <0.01 | 0.2 (<0.01-0.3) | 0.4 (<0.01-3.2) | 1.4 (0.5-4.5) | 1.9 (0.4-3.9) | 2.3 (1.4-5.0) | 2.9 (0.6-11) | 3.8 (0.5-17) | 2.7 (1.0-10) |
| **TM CD4+ CD183+ CD194+ CD196+ CCR10+** |  |  |  |  |  |  |  |  |  |
| % blood samples | 0% | 50% | 83.30% | 100% | 100% | 100% | 100% | 100% | 100% |
| Cells/μL | <0.01 | 0.05 (<0.01-0.1) | 0.9 (<0.01-3.5) | 1.1 (0.5-6.0) | 1.8 (0.7-3.7) | 2.5 (1.1-8.1) | 3.8 (0.8-15) | 3.4 (0.6-13) | 1.8 (0.9-11) |
| **EM CD4+ CD183+ CD194+ CD196+ CCR10+** |  |  |  |  |  |  |  |  |  |
| % blood samples | 0% | 0% | 66.70% | 100% | 100% | 100% | 100% | 100% | 100% |
| Cells/μL | <0.01 | <0.01 | 0.5 (<0.01-1.2) | 1.0 (0.4-4.5) | 1.2 (0.6-2.9) | 2.5 (0.7-4.5) | 2.3 (0.9-9.5) | 1.5 (0.1-8.6) | 1.4 (0.6-7.5) |
| **TE CD4+ CD183+ CD194+ CD196+ CCR10+** |  |  |  |  |  |  |  |  |  |
| % blood samples | 0% | 0% | 0% | 0% | 0% | 100% | 52.9% | 68.2% | 35.7% |
| Cells/μL | <0.01 | <0.01 | <0.01 | <0.01 | <0.01 | 0.2 (0.01-0.7) | 0.02 (<0.01-0.6) | 0.2 (<0.01-1.2) | <0.01 (<0.01-0.4) |
| **CD4+ CD183+ CD194+ CD196+ CCR10-** |  |  |  |  |  |  |  |  |  |
| % blood samples | 100% | 100% | 100% | 100% | 100% | 100% | 100% | 100% | 100% |
| Cells/μL | 4.1 (1.4-9.3) | 29 (21-37) | 18 (5.3-52) | 33 (9.0-59) | 24 (17-42) | 35 (17-65) | 51 (17-168) | 44 (12-122) | 37 (9.0-122) |
| **CM CD4+ CD183+ CD194+ CD196+ CCR10-** |  |  |  |  |  |  |  |  |  |
| % blood samples | 100% | 100% | 100% | 100% | 100% | 100% | 100% | 100% | 100% |
| Cells/μL | 2.4 (0.7-5.6) | 23 (15-31) | 10.7 (3.5-26) | 20 (5.2-35) | 14 (10-26) | 20 (8.7-38) | 30 (2.8-85) | 25 (7.9-68) | 22 (4.9-74) |
| **TM CD4+ CD183+ CD194+ CD196+ CCR10-** |  |  |  |  |  |  |  |  |  |
| % blood samples | 100% | 100% | 100% | 100% | 100% | 100% | 100% | 100% | 100% |
| Cells/μL | 1.8 (0.7-3.7) | 4.2 (4.0-4.4) | 5.6 (1.3-20) | 6.0 (2.0-15) | 5.3 (3.2-11) | 13 (5.1-19) | 17 (6.5-61) | 10 (2.2-35) | 8.3 (2.3-37) |
| **EM CD4+ CD183+ CD194+ CD196+ CCR10-** |  |  |  |  |  |  |  |  |  |
| % blood samples | 0% | 100% | 100% | 100% | 100% | 100% | 100% | 100% | 100% |
| Cells/μL | <0.01 | 1.3 (1.1-1.5) | 1.4 (0.6-5.9) | 3.6 (1.9-8.7) | 3.6 (2.0-9.0) | 4.5 (1.8-14) | 6.8 (1.4-21) | 5.2 (1.1-22) | 5.7 (0.8-14) |
| **TE CD4+ CD183+ CD194+ CD196+ CCR10-** |  |  |  |  |  |  |  |  |  |
| % blood samples | 0% | 0% | 0% | 0% | 0% | 83.3% | 52.9% | 81.8% | 50% |
| Cells/μL | <0.01 | <0.01 | <0.01 | <0.01 | <0.01 | 0.2 (<0.01-1.4) | 0.03 (<0.01-0.9) | 0.1 (<0.01-0.9) | 0.01 (<0.01-0.6) |
| **CD4+ CD183+ CD194+ CD196- CCR10+** |  |  |  |  |  |  |  |  |  |
| % blood samples | 40% | 100% | 100% | 100% | 100% | 100% | 100% | 100% | 100% |
| Cells/μL | <0.01(<0.01-3.7) | 1.5 (0.9-2.1) | 2.6 (0.3-6.3) | 2.9 (2.0-15) | 3.5 (0.7-8.7) | 4.1 (2.0-6.7) | 6.4 (2.2-20) | 5.5 (1.3-13) | 7.2 (2.6-22) |
| **CM CD4+ CD183+ CD194+ CD196- CCR10+** |  |  |  |  |  |  |  |  |  |
| % blood samples | 40% | 100% | 100% | 100% | 100% | 100% | 100% | 100% | 100% |
| Cells/μL | <0.01(<0.01-3.7) | 1.2 (0.7-1.7) | 1.8 (0.2-3.3) | 2.0 (1.2-9.3) | 2.0 (0.3-5.7) | 2.3 (1.1-4.2) | 3.9 (0.7-13) | 3.8 (0.8-10) | 4.8 (2.1-18) |
| **TM CD4+ CD183+ CD194+ CD196- CCR10+** |  |  |  |  |  |  |  |  |  |
| % blood samples | 20% | 100% | 83,3% | 100% | 100% | 100% | 100% | 100% | 100% |
| Cells/μL | <0.01(<0.01-1.3) | 0.3 (0.1-0.4) | 0.4 (<0.01-1.5) | 0.8 (0.3-3.5) | 0.6 (0.2-1.8) | 0.7 (0.5-2.8) | 1.6 (0.6-4.2) | 0.9 (0.3-3.3) | 1.1 (0.2-4.8) |

**Supplementary Table 9** *(Continued)*

|  | **CB (n=15)** | **<2 y (n=2)** | **2-4 y (n=6)** | **5-9 y (n=12)** | **10-17 y (n=13)** | **18-39 y (n=12)** | **40-59 y (n=17)** | **60-79 y (n=22)** | **>80 y (n=14)** |
| --- | --- | --- | --- | --- | --- | --- | --- | --- | --- |
| **EM CD4+ CD183+ CD194+ CD196- CCR10+** |  |  |  |  |  |  |  |  |  |
| % blood samples | 0% | 0% | 66,7% | 100% | 84.60% | 100% | 100% | 100% | 100% |
| Cells/μL | <0.01 | <0.01 | 0.3 (<0.01-1.4) | 0.4 (0.3-2.5) | 0.5 (<0.01-2.4) | 0.5 (0.1-0.7) | 1.0 (0.1-3.5) | 0.7 (0.04-1.8) | 0.9 (0.2-2.0) |
| **TE CD4+ CD183+ CD194+ CD196- CCR10+** |  |  |  |  |  |  |  |  |  |
| % blood samples | 0% | 0% | 0% | 0% | 0% | 66.7% | 23.5% | 68.2% | 35.7% |
| Cells/μL | <0.01 | <0.01 | <0.01 | <0.01 | <0.01 | 0.05 (<0.01-0.3) | <0.01 (<0.01-0.3) | 0.04 (<0.01-0.6) | <0.01 (<0.01-0.1) |
| **CD4+ CD183+ CD194+ CD196- CCR10-** |  |  |  |  |  |  |  |  |  |
| % blood samples | 100% | 100% | 100% | 100% | 100% | 100% | 100% | 100% | 100% |
| Cells/μL | 22 (7.1-66) | 75 (74-76) | 36 (11-114) | 39 (18-106) | <0.01 | 32 (14-53) | 66 (34-115) | 52 (20-123) | 53 (22-182) |
| **CM CD4+ CD183+ CD194+ CD196- CCR10-** |  |  |  |  |  |  |  |  |  |
| % blood samples | 100% | 100% | 100% | 100% | 100% | 100% | 100% | 100% | 100% |
| Cells/μL | 15 (4.4-51) | 66 (61-72) | 28 (9-74) | 31 (13-74) | 19 (12-37) | 23 (9.6-39) | 35 (7.2-87) | 37 (15-90) | 29 (15-132) |
| **TM CD4+ CD183+ CD194+ CD196- CCR10-** |  |  |  |  |  |  |  |  |  |
| % blood samples | 100% | 100% | 100% | 100% | 100% | 100% | 100% | 100% | 100% |
| Cells/μL | 6.9 (2.6-23) | 7.2 (4.5-9.8) | 5.3 (1.0-31) | 6.3 (1.8-19) | 4.6 (3.2-7.7) | 7.6 (3.0-12) | 17 (4.8-43) | 7.1 (2.4-26) | 7.4 (2.5-36) |
| **EM CD4+ CD183+ CD194+ CD196- CCR10-** |  |  |  |  |  |  |  |  |  |
| % blood samples | 0% | 50% | 100% | 100% | 100% | 100% | 100% | 100% | 100% |
| Cells/μL | <0.01 | 1.6 (<0.01-3.3) | 1.8 (0.5-9.1) | 2.7 (1.1-13) | 2.8 (1.2-6.8) | 2.5 (1.4-7.9) | 7.1 (3.7-22) | 6.0 (0.5-18) | 6.2 (2.5-29) |
| **TE CD4+ CD183+ CD194+ CD196- CCR10-** |  |  |  |  |  |  |  |  |  |
| % blood samples | 0% | 0% | 0% | 0% | 0% | 100% | 88.2% | 86.4% | 85.7% |
| Cells/μL | <0.01 | <0.01 | <0.01 | <0.01 | <0.01 | 0.4 (0.1-3.0) | 0.4 (<0.01-3.5) | 0.9 (<0.01-8.0) | 0.7 (<0.01-3.4) |
| **CD4+ CD183+ CD194- CD196+ CCR10+** |  |  |  |  |  |  |  |  |  |
| % blood samples | 0% | 0% | 0% | 16.70% | 23.10% | 91.7% | 88.2% | 90.9% | 71.4% |
| Cells/μL | <0.01 | <0.01 | <0.01 | <0.01(<0.01-0.1) | <0.01 | 0.2 (<0.01-4.2) | 0.4 (<0.01-3.5) | 0.3 (<0.01-2.2) | 0.5 (<0.01-3.3) |
| **CM CD4+ CD183+ CD194- CD196+ CCR10+** |  |  |  |  |  |  |  |  |  |
| % blood samples | 0% | 0% | 0% | 16.70% | 23.10% | 91.7% | 70.6% | 81.8% | 64.3% |
| Cells/μL | <0.01 | <0.01 | <0.01 | <0.01(<0.01-0.1) | <0.01 | 0.05 (<0.01-1.2) | 0.1 (<0.01-0.8) | 0.09 (<0.01-0.9) | 0.2 (<0.01-0.8) |
| **TM CD4+ CD183+ CD194- CD196+ CCR10+** |  |  |  |  |  |  |  |  |  |
| % blood samples | 0% | 0% | 0% | 0% | 7.70% | 91.7% | 76.5% | 72.7% | 71.4% |
| Cells/μL | <0.01 | <0.01 | <0.01 | <0.01 | <0.01 | 0.1 (<0.01-2.2) | 0.1 (<0.01-2.0) | 0.1 (<0.01-1.0) | 0.2 (<0.01-1.4) |
| **EM CD4+ CD183+ CD194- CD196+ CCR10+** |  |  |  |  |  |  |  |  |  |
| % blood samples | 0% | 0% | 0% | 0% | 0% | 58.3% | 82.4% | 59.1% | 57.1% |
| Cells/μL | <0.01 | <0.01 | <0.01 | <0.01 | <0.01 | 0.02 (<0.01-0.6) | 0.04 (<0.01-0.7) | 0.03 (<0.01-0.4) | 0.07 (<0.01-1.0) |
| **TE CD4+ CD183+ CD194- CD196+ CCR10+** |  |  |  |  |  |  |  |  |  |
| % blood samples | 0% | 0% | 0% | 0% | 0% | 25% | 0% | 22.7% | 21.4% |
| Cells/μL | <0.01 | <0.01 | <0.01 | <0.01 | <0.01 | <0.01 (<0.01-0.1) | <0.01 | <0.01 (<0.01-0.1) | <0.01 (<0.01-0.1) |

**Supplementary Table 9** *(Continued)*

|  | **CB (n=15)** | **<2 y (n=2)** | **2-4 y (n=6)** | **5-9 y (n=12)** | **10-17 y (n=13)** | **18-39 y (n=12)** | **40-59 y (n=17)** | **60-79 y (n=22)** | **>80 y (n=14)** |
| --- | --- | --- | --- | --- | --- | --- | --- | --- | --- |
| **CD4+ CD183+ CD194- CD196- CCR10+** |  |  |  |  |  |  |  |  |  |
| % blood samples | 6.70% | 50% | 0% | 83.30% | 76.90% | 100% | 88.2% | 90.1% | 78.6% |
| Cells/μL | <0.01(<0.01-0.06) | 0.08 (<0.01-0.2) | <0.01 | 0.2 (<0.01-1.7) | 0.2 (<0.01-1.2) | 0.2 (0.05-0.8) | 0.5 (<0.01-3.4) | 0.4 (<0.01-2.2) | 0.3 (<0.01-1.8) |
| **CM CD4+ CD183+ CD194- CD196- CCR10+** |  |  |  |  |  |  |  |  |  |
| % blood samples | 6.70% | 50% | 0% | 66.70% | 61.50% | 91.7% | 76.5% | 86.4% | 78.6% |
| Cells/μL | <0.01(<0.01-0.06) | 0.08 (<0.01-0.2) | <0.01 | 0.1 (<0.01-1.1) | 0.07 (<0.01-0.4) | 0.1 (<0.01-0.5) | 0.2 (<0.01-2.0) | 0.2 (<0.01-1.4) | 0.1 (<0.01-0.8) |
| **TM CD4+ CD183+ CD194- CD196- CCR10+** |  |  |  |  |  |  |  |  |  |
| % blood samples | 0% | 0% | 0% | 33.30% | 38.50% | 83.3% | 58.8% | 77.3% | 57.1% |
| Cells/μL | <0.01 | <0.01 | <0.01 | <0.01 (<0.01-0.2) | <0.01 (<0.01-0.4) | 0.04 (<0.01-0.3) | 0.03 (<0.01-0.8) | 0.06 (<0.01-0.4) | 0.04 (<0.01-0.4) |
| **EM CD4+ CD183+ CD194- CD196- CCR10+** |  |  |  |  |  |  |  |  |  |
| % blood samples | 0% | 0% | 0% | 8.30% | 30.80% | 58.3% | 52.9% | 50.0% | 64.3% |
| Cells/μL | <0.01 | <0.01 | <0.01 | <0.01 (<0.01-0.4) | <0.01 (<0.01-0.7) | 0.02 (<0.01-0.3) | 0.04 (<0.01-0.6) | <0.01 (<0.01-0.3) | 0.05 (<0.01-0.3) |
| **TE CD4+ CD183+ CD194- CD196- CCR10+** |  |  |  |  |  |  |  |  |  |
| % blood samples | 0% | 0% | 0% | 0% | 0% | 41.7% | 29.4% | 50.0% | 42.9% |
| Cells/μL | <0.01 | <0.01 | <0.01 | <0.01 | <0.01 | <0.01 (<0.01-0.1) | <0.01 (<0.01-0.1) | 0.01 (<0.01-0.4) | <0.01 (<0.01-0.3) |
| **CD4+ CD183- CD194- CD196+ CCR10-** |  |  |  |  |  |  |  |  |  |
| % blood samples | 60% | 50% | 100% | 100% | 100% | 100% | 100% | 100% | 100% |
| Cells/μL | 0.6(<0.01-6.3) | 4.4 (4.4-4.5) | 3.3 (1.5-6.9) | 4.7 (1.1-9.1) | 3.4 (2.0-9.0) | 3.9 (1.5-7.2) | 6.6 (1.8-15) | 7.8 (2.0-31) | 7.1 (0.8-10) |
| **CM CD4+ CD183- CD194- CD196+ CCR10-** |  |  |  |  |  |  |  |  |  |
| % blood samples | 60% | 50% | 100% | 100% | 100% | 100% | 100% | 100% | 100% |
| Cells/μL | 0.6(<0.01-6.3) | 3.5 (3.2-3.7) | 2.0 (1.0-3.9) | 3.5 (0.9-6.6) | 2.0 (1.2-6.2) | 3.2 (1.0-4.6) | 3.6 (0.5-14) | 5.9 (1.3-28) | 4.7 (0.6-8.7) |
| **TM CD4+ CD183- CD194- CD196+ CCR10-** |  |  |  |  |  |  |  |  |  |
| % blood samples | 6.70% | 50% | 100% | 100% | 100% | 100% | 100% | 100% | 100% |
| Cells/μL | <0.01(<0.01-0.6) | 0.8 (0.6-1.0) | 0.6 (0.3-2.6) | 0.6 (0.1-2.5) | 0.6 (0.3-1.8) | 0.7 (0.1-1.5) | 0.9 (0.04-5.4) | 0.7 (0.2-2.7) | 0.7 (0.05-4.5) |
| **EM CD4+ CD183- CD194- CD196+ CCR10-** |  |  |  |  |  |  |  |  |  |
| % blood samples | 0% | 50% | 100% | 91.70% | 92.30% | 83.3% | 94.1% | 100.0% | 85.7% |
| Cells/μL | <0.01 | 0.2 (0.1-0.2) | 0.2 (0.1-0.7) | 0.3 (<0.01-2.3) | 0.5 (<0.01-1.6) | 0.1 (<0.01-1.5) | 0.3 (<0.01-0.5) | 0.2 (0.02-1.2) | 0.3 (<0.01-1.2) |
| **TE CD4+ CD183- CD194- CD196+ CCR10-** |  |  |  |  |  |  |  |  |  |
| % blood samples | 0% | 0% | 0% | 0% | 0% | 66.7% | 29.4% | 59.1% | 21.4% |
| Cells/μL | <0.01 | <0.01 | <0.01 | <0.01 | <0.01 | 0.03 (<0.01-0.2) | <0.01 (<0.01-0.2) | 0.04 (<0.01-0.9) | <0.01 (<0.01-0.3) |

**Supplementary Table 9** *(Continued)*

|  | **CB (n=15)** | **<2 y (n=2)** | **2-4 y (n=6)** | **5-9 y (n=12)** | **10-17 y (n=13)** | **18-39 y (n=12)** | **40-59 y (n=17)** | **60-79 y (n=22)** | **>80 y (n=14)** |
| --- | --- | --- | --- | --- | --- | --- | --- | --- | --- |
| **CD4+ CD183- CD194+ CD196- CCR10+** |  |  |  |  |  |  |  |  |  |
| % blood samples | 73.30% | 50% | 100% | 100% | 100% | 100% | 100% | 100% | 100% |
| Cells/μL | 0.7 (<0.01-3.7) | 0.9 (0.7-1.0) | 2.4 (0.8-3.4) | 2.7 (1.1-6.5) | 2.0 (0.9-6.5) | 3.9 (2.0-15) | 7.2 (2.6-18) | 7.0 (1.3-55) | 6.4 (3.2-13) |
| **CM CD4+ CD183- CD194+ CD196- CCR10+** |  |  |  |  |  |  |  |  |  |
| % blood samples | 73.30% | 50% | 100% | 100% | 100% | 100% | 100% | 100% | 100% |
| Cells/μL | 0.6 (<0.01-3.7) | 0.8 (0.7-0.9) | 2.0 (0.6-2.9) | 1.9 (0.6-4.1) | 1.5 (0.4-4.0) | 3.0 (1.4-12) | 4.0 (0.1-16) | 5.2 (1.0-52) | 5.0 (2.3-9.2) |
| **TM CD4+ CD183- CD194+ CD196- CCR10+** |  |  |  |  |  |  |  |  |  |
| % blood samples | 26.70% | 0% | 83.30% | 83.30% | 61.50% | 91.70% | 91.70% | 95.50% | 100% |
| Cells/μL | <0.01 (<0.01-1.2) | <0.01 | 0.2 (<0.01-0.4) | 0.1 (<0.01-2.4) | 0.09 (<0.01-0.8) | 0.2 (<0.01-1.7) | 0.5 (<0.01-3.9) | 0.3 (<0.01-1.1) | 0.4 (0.1-1.9) |
| **EM CD4+ CD183- CD194+ CD196- CCR10+** |  |  |  |  |  |  |  |  |  |
| % blood samples | 0% | 0% | 66.70% | 91.70% | 84.60% | 100% | 100% | 100% | 100% |
| Cells/μL | <0.01 | <0.01 | 0.1 (<0.01-0.4) | 0.4 (<0.01-1.0) | 0.4 (<0.01-2.2) | 0.8 (0.2-1.4) | 0.6 (0.04-2.4) | 0.8 (0.2-2.4) | 0.9 (0.07-4.3) |
| **TE CD4+ CD183- CD194+ CD196- CCR10+** |  |  |  |  |  |  |  |  |  |
| % blood samples | 0% | 0% | 0% | 0% | 0% | 66.70% | 52.90% | 68.20% | 42.90% |
| Cells/μL | <0.01 | <0.01 | <0.01 | <0.01 | <0.01 | 0.03 (<0.01-0.9) | 0.03 (<0.01-0.5) | 0.07 (<0.01-0.5) | <0.01 (<0.01-0.5) |
| **Non-naïve CD4+ CD183- CD194- CD196- CCR10-** |  |  |  |  |  |  |  |  |  |
| % blood samples | 26.70% | 50% | 100% | 100% | 92.30% | 100% | 100% | 100% | 93% |
| Cells/μL | <0.01(<0.01-6.1) | 31 (<0.01-63) | 10.2 (3.9-49) | 6.7 (2.6-33) | 4.7 (<0.01-27) | 2.1 (0.4-9.9) | 5.9 (1.1-231) | 12 (0.8-1287) | 2.4 (<0.01-313) |
| **CD4+ Tregs** |  |  |  |  |  |  |  |  |  |
| % blood samples | 100% | 100% | 100% | 100% | 100% | 100% | 100% | 100% | 100% |
| Cells/μL | 133 (66-213) | 242 (188-297) | 78 (48-290) | 120 (49-192) | 67 (33-97) | 50 (30-95) | 62 (22-141) | 61 (31-132) | 50 (19-115) |
| **Naïve Treg** |  |  |  |  |  |  |  |  |  |
| % blood samples | 100% | 100% | 100% | 100% | 100% | 100% | 100% | 100% | 100% |
| Cells/μL | 93 (45-130) | 133 (77-189) | 36 (16-220) | 51 (14-132) | 25 (9.7-54) | 13 (9.2-37) | 5.6 (0.5-33) | 6.0 (1.1-17) | 4 (1.2-14) |
| **Th1-like Treg** |  |  |  |  |  |  |  |  |  |
| % blood samples | 93.3% | 100% | 100% | 100% | 100% | 100% | 100% | 100% | 100% |
| Cells/μL | 3.1 (<0.01-5.1) | 8.1 (4.5-11.6) | 8.2 (3.9-12) | 4.8 (1.8-21) | 3.8 (1.6-7.0) | 2.6 (0.9-5.9) | 3.1 (1.0-15) | 3.2 (0.9-9.7) | 2.7 (0.4-12) |
| **Th2-like Treg** |  |  |  |  |  |  |  |  |  |
| % blood samples | 100% | 100% | 100% | 100% | 100% | 100% | 100% | 100% | 100% |
| Cells/μL | 15 (3.1-28) | 41 (39-43) | 10 (4.7-28) | 9.3 (3.0-17) | 4.8 (2.2-10) | 3.5 (1.2-8.2) | 3.9 (1.4-11) | 4.9 (1.4-17) | 3.9 (1.6-10) |
| **Th17-like Treg** |  |  |  |  |  |  |  |  |  |
| % blood samples | 100% | 100% | 100% | 100% | 100% | 100% | 100% | 100% | 100% |
| Cells/μL | 6.8 (3.9-20) | 23 (22-24) | 6.4 (3.5-11) | 8.9 (4.3-16) | 6.8 (3.8-12) | 8.0 (3.1-14) | 10 (2.0-23) | 10 (5.9-28) | 10 (3.9-21) |

**Supplementary Table 9** *(Continued)*

|  | **CB (n=15)** | **<2 y (n=2)** | **2-4 y (n=6)** | **5-9 y (n=12)** | **10-17 y (n=13)** | **18-39 y (n=12)** | **40-59 y (n=17)** | **60-79 y (n=22)** | **>80 y (n=14)** |
| --- | --- | --- | --- | --- | --- | --- | --- | --- | --- |
| **Th1/Th17-like Treg** |  |  |  |  |  |  |  |  |  |
| % blood samples | 6.7% | 100% | 100% | 91.7% | 100% | 100% | 100% | 100% | 92.9% |
| Cells/μL | <0.01 (<0.01-0.4) | 0.5 (0.1-0.9) | 0.3 (0.2-2.1) | 0.8 (<0.01-1.4) | 0.6 (0.08-1.0) | 0.4 (0.07-1.3) | 0.9 (0.1-2.0) | 0.8 (0.2-4.0) | 0.7 (<0.01-2.0) |
| **Th22-like Treg** |  |  |  |  |  |  |  |  |  |
| % blood samples | 46.7% | 100% | 100% | 100% | 100% | 100% | 100% | 100% | 100% |
| Cells/μL | <0.01 (<0.01-1.9) | 3.6 (3.3-3.9) | 3.8 (1.8-9.8) | 6.2 (2.4-15) | 3.9 (1.6-11) | 6.3 (1.8-12) | 7.2 (2.0-15) | 7.4 (1.6-17) | 6.5 (3.0-11) |
| **CD183+ CD194+ CD196- CCR10+ Treg** |  |  |  |  |  |  |  |  |  |
| % blood samples | 46.7% | 100% | 100% | 100% | 100% | 100% | 100% | 100% | 100% |
| Cells/μL | <0.01 (<0.01-2.1) | 0.9 (0.3-1.5) | 1.1 (0.8-3.1) | 1.8 (0.5-4.9) | 1.0 (0.4-5.3) | 0.7 (0.3-1.2) | 1.0 (0.4-4.6) | 1.1 (0.5-3.7) | 1.1 (0.4-7.3) |
| **CD183+ CD194+ CD196- CCR10- Treg** |  |  |  |  |  |  |  |  |  |
| % blood samples | 100% | 100% | 100% | 100% | 100% | 100% | 100% | 100% | 100% |
| Cells/μL | 10 (4.2-17) | 18 (17-19) | 7.3 (4.0-14) | 8.1 (2.0-17) | 4.8 (2.4-8.0) | 3.0 (1.4-5.7) | 4.7 (2.0-15) | 4.7 (2.5-15) | 4.1 (1.7-15) |
| **CD183+ CD194+ CD196+ CCR10- Treg** |  |  |  |  |  |  |  |  |  |
| % blood samples | 100% | 100% | 100% | 100% | 100% | 100% | 100% | 100% | 100% |
| Cells/μL | 5.4 (2.4-11.2) | 11 (10-12) | 3.7 (0.7-9.3) | 6.0 (1.8-12) | 5.1 (2.8-7.1) | 5.4 (2.2-11) | 8.6 (2.1-25) | 9.8 (3.4-21) | 10 (2.8-20) |
| **CD183+ CD194+ CD196+ CCR10+ Treg** |  |  |  |  |  |  |  |  |  |
| % blood samples | 33.3% | 100% | 83.3% | 100% | 100% | 100% | 100% | 100% | 100% |
| Cells/μL | <0.01 (<0.01-0.7) | 0.5 (0.3-0.7) | 0.4 (<0.01-2.3) | 1.3 (0.09-3.1) | 1.1 (0.09-4.4) | 2.5 (0.7-5.9) | 2.8 (1.0-16) | 3.8 (0.8-12) | 4.6 (1.5-11) |
| **CD183- CD194+ CD196- CCR10+ Treg** |  |  |  |  |  |  |  |  |  |
| % blood samples | 33.3% | 100% | 100% | 100% | 100% | 100% | 100% | 100% | 100% |
| Cells/μL | <0.01 (<0.01-1.0) | 0.8 (0.6-0.9) | 1.3 (0.8-3.7) | 1.8 (0.8-4.6) | 1.1 (0.4-2.9) | 1.0 (0.4-2.6) | 1.3 (0.3-4.9) | 1.2 (0.5-3.6) | 1.0 (0.2-1.8) |
| **CD4+ TFH cells** |  |  |  |  |  |  |  |  |  |
| % blood samples | 33.3% | 100% | 100% | 100% | 100% | 100% | 100% | 100% | 100% |
| Cells/μL | <0.01 (<0.01-41) | 101 (23-179) | 78 (42-173) | 119 (72-154) | 76 (42-127) | 100 (55-150) | 111 (45-240) | 163 (68-325) | 113 (33-206) |
| **Treg TFH** |  |  |  |  |  |  |  |  |  |
| % blood samples | 33.3% | 100% | 100% | 100% | 100% | 100% | 100% | 100% | 100% |
| Cells/μL | <0.01 (<0.01-3.3) | 4.4 (1.8-7.0) | 5.1 (2.0-7.2) | 9.6 (2.5-21) | 7.6 (1.7-23) | 6.4 (2.8-10) | 6.4 (0.3-21) | 12 (5.3-53) | 10 (2.2-31) |
| **Th1-like TFH** |  |  |  |  |  |  |  |  |  |
| % blood samples | 0% | 100% | 100% | 100% | 100% | 100% | 100% | 100% | 100% |
| Cells/μL | <0.01 | 13 (2.0-24) | 13 (8.3-25) | 19 (8.8-29) | 11 (2.0-23) | 15 (9.4-30) | 13 (3.2-52) | 19 (5.2-50) | 13 (2.5-33) |
| **Th2-like TFH** |  |  |  |  |  |  |  |  |  |
| % blood samples | 0% | 100% | 100% | 100% | 100% | 100% | 100% | 100% | 100% |
| Cells/μL | <0.01 | 23 (4.7-41) | 13 (6.8-35) | 16 (8.7-24) | 8.9 (2.9-15) | 9.1 (3.1-19) | 10 (3.6-15) | 10 (4.2-37) | 7.4 (5.0-15) |

**Supplementary Table 9** *(Continued)*

|  | **CB (n=15)** | **<2 y (n=2)** | **2-4 y (n=6)** | **5-9 y (n=12)** | **10-17 y (n=13)** | **18-39 y (n=12)** | **40-59 y (n=17)** | **60-79 y (n=22)** | **>80 y (n=14)** |
| --- | --- | --- | --- | --- | --- | --- | --- | --- | --- |
| **Th17-like TFH** |  |  |  |  |  |  |  |  |  |
| % blood samples | 0% | 100% | 100% | 100% | 100% | 100% | 100% | 100% | 100% |
| Cells/μL | <0.01 | 15 (5.8-24) | 12 (5.4-25) | 19 (15-27) | 12 (3.9-27) | 20 (11-34) | 22 (8.9-48) | 31 (12-72) | 23 (9.3-41) |
| **Th1/Th17-like TFH** |  |  |  |  |  |  |  |  |  |
| % blood samples | 0% | 100% | 100% | 100% | 100% | 100% | 100% | 100% | 100% |
| Cells/μL | <0.01 | 0.8 (0.4-1.1) | 0.9 (0.5-5.0) | 2.4 (1.0-4.1) | 1.7 (0.3-4.6) | 5.4 (1.8-10) | 5.3 (0.6-18) | 7.0 (1.3-40) | 4.3 (0.7-17) |
| **CD183+ CD194+ CD196- CCR10- TFH** |  |  |  |  |  |  |  |  |  |
| % blood samples | 6.7% | 100% | 100% | 100% | 100% | 100% | 100% | 100% | 100% |
| Cells/μL | <0.01 (<0.01-3.0) | 23 (2.2-43) | 16 (5.5-29) | 17 (11-30) | 9.3 (2.7-17) | 12 (4.9-18) | 11 (6.1-29) | 14 (5.4-57) | 11 (4.3-25) |
| **CD183+ CD194+ CD196+ CCR10- TFH** |  |  |  |  |  |  |  |  |  |
| % blood samples | 0% | 100% | 100% | 100% | 100% | 100% | 100% | 100% | 100% |
| Cells/μL | <0.01 | 4.3 (2.4-6.2) | 4.8 (1.3-14) | 7.4 (2.6-11) | 5.7 (0.5-9.2) | 8.6 (4.0-21) | 11 (3.5-26) | 10 (3.3-30) | 9.7 (1.8-27) |
| **CD183- CD194- CD196+ CCR10- TFH** |  |  |  |  |  |  |  |  |  |
| % blood samples | 0% | 100% | 100% | 100% | 100% | 100% | 100% | 100% | 100% |
| Cells/μL | <0.01 | 1.8 (0.9-2.7) | 1.8 (1.3-5.1) | 4.0 (1.9-8.4) | 2.8 (0.6-4.8) | 6.7 (2.7-11) | 11 (1.8-21) | 13 (3.1-48) | 8.2 (1.5-21) |
| **CD183- CD194- CD196- CCR10- TFH** |  |  |  |  |  |  |  |  |  |
| % blood samples | 0% | 100% | 100% | 91.7% | 92.3% | 100% | 100% | 100% | 85.7% |
| Cells/μL | <0.01 | 0.09 (<0.01-0.2) | 5.9 (2.6-16) | 4.8 (<0.01-12) | 2.6 (<0.01-4.3) | 0.6 (0.1-2.3) | 1.1 (0.2-34) | 1.4 (0.1-6.3) | 0.7 (<0.01-19) |
| **CD4+ CD185+ CD27+ CD45RA+ CD62L+ T cells** |  |  |  |  |  |  |  |  |  |
| % blood samples | 33.3% | 100% | 100% | 100% | 100% | 100% | 100% | 100% | 100% |
| Cells/μL | <0.01 (<0.01-38) | 16 (2.8-29) | 7.3 (3.7-43) | 11 (2.7-36) | 9.5 (1.6-66) | 12 (6.6-33) | 10 (2.9-56) | 17 (7.5-42) | 9.3 (3.2-25) |

*Results expressed as percentage of blood samples with detectable cells, and median absolute numbers of cells per microliter (range).*

*<0.01 cells/μL: sensitivity level for cell detection with the flowcytometric method used.*
