# Supplementary material for: Age Distribution of Multiple Functionally Relevant Subsets of CD4+ T Cells in Human Blood Using a Standardized and Validated 14-Color EuroFlow Immune Monitoring Tube
Source: Front Immunol. 2020 Feb 27;11:166. doi: 10.3389/fimmu.2020.00166 (PMC7056740; doi:10.3389/fimmu.2020.00166)
Supplement: Supplementary file 10 [file Data_Sheet_2.docx]

**Supplementary Methods (Botafogo *et al*):**

***Design of the EuroFlow-IMM TCD4 antibody combination.*** For every flow cytometric assay performed, EuroFlow standard operating procedures (SOPs) for sample preparation and staining were used. Staining was performed in 100μL of peripheral blood (PB) to which the appropriate volume of fluorochrome-conjugated antibody reagents directed against cell surface markers was added and then, sample incubation was performed for 30 minutes at room temperature (RT). When only staining for cell surface markers was performed, 2 mL of FACS lysing solution -Becton/Dickinson Biosciences (BD), San Jose, CA- diluted 1:10 (vol/vol) in distilled water was subsequently added, followed by an incubation for another 10 minutes at RT, and a washing step, after which cells were resuspended in 500 μL of phosphate buffered saline containing 0.2% Bovine Serum Albumin (PBS-BSA). In turn, when surface membrane plus cytoplasmic staining was performed, after surface membrane staining, the cell suspension was washed and incubated with solution A of the Fix & Perm Reagent Kit (Nordic-MUbio, Susteren, The Netherlands) for 15 minutes at RT. Afterward, washed cells were incubated for another 15 minutes at RT with solution B of the Fix & Perm Reagent Kit and antibodies against the intracellular markers. Stained cells were washed once and resuspended in 500 μL of PBS-BSA. For both protocols, cells were acquired immediately after sample preparation was completed or stored at 4°C for a maximum of 1 hour until acquisition in the flow cytometer. All EuroFlow SOPs are freely available in full at www.EuroFlow.org.

***In vitro T-cell stimulation assays.*** For direct correlation between specific CD4+ Th-cell phenotypes and intracellular cytokine production, and to select for an optimal activation T-cell marker, short-term *in vitro* cell culture assays were performed. Briefly, 2 aliquots of whole PB containing 10μg/mL of Brefeldin A were stimulated *in vitro* for 4h and 6h with either 0.025μg/mL of phorbol 12-myristate 13 acetate (PMA) (Sigma-Aldrich Inc, Darmstadt, Germany) plus 1μg/mL of ionomycin (Sigma-Aldrich Inc) or 5μg/mL of a whole cytomegalovirus (CMV) lysate (Advanced Biotechnologies Inc, Columbia, MD), respectively. Stimulated cells were subsequently stained with a panel of monoclonal antibodies (Mabs) (Supplementary Table 2), following the EuroFlow standard operating procedure (SOP) for simultaneous staining of surface membrane (Sm) and cytoplasmic (Cy) markers ([www.EuroFlow.org](http://www.EuroFlow.org)) described above.

***Gene expression profiling (GEP) assays.*** Twenty-two different CD4+ T-cell populations from 6 healthy adult peripheral blood (PB) samples were sorted from Biocoll-enriched mononuclear cells in a FACSAria III flow cytometer (BD) using the following 9-color monoclonal antibody (Mab) combination: CCR10 brilliant violet (BV)421, CD127 BV510, CD25 VioBright-fluorescein isothiocyanate (FITC), CD3 peridinin chlorophyll protein (PerCP)/Cy5.5, CD183 phycoerythrin (PE), CD196 PE-CF594, CD194 PE-Cyanin 7 (PE-Cy7), CD185 allophycocyanin (APC) and CD4 APC-hilite 7 (APC-H7). The phenotypic profiles used to identify and sort each of the 22 CD4+ T-cell subsets, as well as the precise gating strategy applied for their identification and sorting are described and illustrated in Supplementary Table 3 and in Supplementary Figure 1, respectively; of note, naïve CD4+ T-cells were identified for this assay based on their lack of expression of all chemokine receptors tested (i.e. CD183, CD185, CD194, CD196 and CCR10), together with a SSC^lo^ CD25^-^ CD127^+^naïve-associated phenotypic profile. A minimum of 1x10^4^ cells per cell population was FACS-sorted (purity >97%) and placed in RA1 buffer (Macherey-Nagel, Düren, Germany) at identical concentrations per cell population. Total RNA was extracted from each sorted CD4+ T-cell subset using the NucleoSpin® RNA XS kit (Macherey-Nagel, Düren, Germany), and cDNAs generated from total RNA using the Fluidigm Reverse Transcription Master Mix (Fluidigm, San Francisco, CA) following the instructions of the manufacturer. Afterward, cDNA specific target preamplification (STA reaction) was performed using the PreAmp Master Mix (Fluidigm), according to the recommendations of the manufacturer. Briefly, 1.25μL of each cDNA was mixed with 0.5μL of pooled DeltaGene primers (500nM) and the PreAmp Master Mix. Subsequently, each sample was treated with exonuclease I to eliminate the unbound primers. Preamplified cDNA was then used for quantitative real-time polymerase chain reaction (qPCR) measurements of each gene using the BioMark HD system, as per the manufacturer’s instructions. Briefly, 2.25 μL of each amplified cDNA was mixed with 2.5 μL of 2X SsoFast EvaGreen Supermix with Low ROX (Bio-Rad, Hercules, CA) and 0.25 μL of 20X DNA Binding Dye Sample Loading Reagent (Fluidigm). Individual qPCR primer pairs (100 mM) were diluted 1:10 (v/v) with Tris-EDTA (for a 2.5 μL total volume), and mixed with 2.5 μL Assay Loading Reagent (Fluidigm). Each sample mix and assay mix was individually pipetted into one sample or assay inlet in a Dynamic Array IFC chip (Fluidigm), respectively. Subsequent sample/assay loading was performed with an IFC Controller HX (Fluidigm), and a qPCR performed in the BioMark HD qPCR reader (Fluidigm) following the manufacturer's instructions based on standard fast cycling conditions and melt-curve analysis; an amplification curve was generated for each gene of interest for each individual sample. Data were analyzed using the qPCR analysis software (Fluidigm) with the following settings: curve quality threshold set at 0.65, linear derivative baseline correction, automatic thresholding by assay, and manual melt curve exclusion. PCR results were analyzed as a qPCR and melting curve analysis (MCA) assay to avoid false positive and false negative results due to analyses of PCR curves only. The melting temperature (Tm) peak detected within the Tm detection range (78ºC-85ºC) validated the amplification curve of the PCR cycle. If no Tm peak was detected, any potential amplification was considered invalid and the quality of the cycle threshold (Ct) was set to zero. In this study, cycle 27 was set as a limit to define whether a gene was expressed or not. Ct values for each reaction were then exported for further analysis.

For data analysis, GEPs raw data was normalized by dividing each gene expression value by their corresponding GAPDH expression value (positive control) in all technical replicates (n=5) obtained for each sample (n=6 PB samples). Average expression values for each of the 85 genes investigated in the technical replicates measured for each sample was calculated, and data was analyzed and represented in a heatmap graphic with the corresponding hierarchical clustering diagram. R-package gplot (R Development Core Team, Vienna, Austria) was used to generate the cluster heatmap (Figure 2) in which averaged gene expression values, standardized as Z-values, are shown in a color code; one-tailed p-values were calculated using multiscale bootstrap resampling. Hierarchical trees were generated with the R-package pvclust algorithm (R Development Core Team v.3.2.3, Vienna, Austria).

***Database construction and inclusion criteria for FCS files.*** The actual criteria used for the selection of appropriate FCS datafiles for the construction of the database were the following: a) minimum number of acquired events of 30x10^3^ T-cell events; b) percentage of debris/doublets <25%; c) homogeneous acquisition vs. time; d) FSC (forward scatter) detector values to reference target of 55,000 (range: 50,000 to 60,000); e) SSC (side scatter) detector values to reference target of 13,000 (range: 11,000 to 15,000); f) acquisition with the appropriate and specific fluorochrome compensation matrix in the flow-cytometer; and g) correct staining, based on external and internal negative and positive controls. In general, samples were excluded when they fulfilled more than one deviation, but in case of critical deviations, only one deviation was required for their exclusion from the database.
